# Supplementary material for: A Robust Metatranscriptomic Technology for Population-Scale Studies of Diet, Gut Microbiome, and Human Health
Source: Int J Genomics. 2019 Oct 1;2019:1718741. doi: 10.1155/2019/1718741 (PMC6791206; doi:10.1155/2019/1718741)
Supplement: Supplementary Materials — Supplementary Table S1: data for the percent ribosomal RNA in stool samples that are processed through Viomega without the custom rRNA depletion method. Supplementary Tables S2–S4: all taxa identified by Viomega in 10,000 human stool samples (strains, species, and genera, respectively) are shown. Supplementary Table S5: the top 100 KEGG functions identified by Viomega in 10,000 human stool samples are shown. [file 1718741.f1.pdf]

Table S1: Percent of sequencing reads assigned to rRNA for stool samples processed through Viomega without the custom rRNA depletion method. Average percent rRNA = 95.7 +/- 1.8%.

**Sample**	**Human Stool RNA pool**	**# of stool RNA samples in pool**	**rRNA, %**
1	A	37	97.26
2			97.51
3			97.31
4	B	42	95.3
5			94.29
6			94.4
7	C	38	93.22
8			92.76
9			93.41
10	D	12	98.09
11			97.85
12			97.89
13	E	12	96.97
14			96.3
15			96.69
16	F	12	94.71
17			94.26
18			93.97

Table S2: All strains identified in 10,000 human stool samples

	Taxonomy ID	Strain name	SuperKingdom	Prevalence in 10,000 samples, %
1	742768	Eggerthella lenta 1_1_60AFAA	Bacteria	97.08
2	411469	[Eubacterium] hallii DSM 3353	Bacteria	93.93
3	546273	Veillonella dispar ATCC 17748	Bacteria	92.34
4	445972	Anaerotruncus colihominis DSM 17241	Bacteria	91.44
5	1650661.1	Clostridium phoceensis strain GD3	Bacteria	90.83
6	411459	Blautia obeum ATCC 29174	Bacteria	89.67
7	39485.2	[Eubacterium] eligens strain 2789STDY5834875	Bacteria	88.93
8	748224	Faecalibacterium cf. prausnitzii KLE1255	Bacteria	88.31
9	411483	Faecalibacterium prausnitzii A2-165	Bacteria	86.05
10	585394	Roseburia hominis A2-183	Bacteria	83.35
11	445970	Alistipes putredinis DSM 17216	Bacteria	79.92
12	1408428	Bilophila wadsworthia ATCC 49260	Bacteria	79.64
13	1737424.1	Blautia massiliensis sp. GD8	Bacteria	78.91
14	709991	Odoribacter splanchnicus DSM 20712	Bacteria	78.15
15	411485	Faecalibacterium prausnitzii M21/2	Bacteria	77.79
16	853.1	Faecalibacterium prausnitzii strain 2789STDY5834970	Bacteria	76.83
17	1715004.1	Clostridiales bacterium KLE1615	Bacteria	76.44
18	717959	Alistipes shahii WAL 8301	Bacteria	76.40
19	1519439.1	Oscillibacter sp. ER4	Bacteria	73.98
20	39491.1	[Eubacterium] rectale strain T1-815	Bacteria	73.62
21	39488.1	[Eubacterium] hallii strain 2789STDY5834835	Bacteria	73.28
22	411477	Parabacteroides merdae ATCC 43184	Bacteria	73.10
23	411471	Subdoligranulum variabile DSM 15176	Bacteria	72.31
24	39490.1	Eubacterium ramulus strain 2789STDY5608891	Bacteria	71.84
25	1384484	Adlercreutzia equolifaciens DSM 19450	Bacteria	70.35
26	999413	[Clostridium] innocuum 2959	Bacteria	70.25
27	428125	[Clostridium] leptum DSM 753	Bacteria	70.16
28	657308	Gordonibacter pamelaeae 7-10-1-b	Bacteria	64.96
29	1697794.1	Clostridia bacterium UC5.1-1D1	Bacteria	63.20
30	1310949	Acinetobacter baumannii 24975_5	Bacteria	62.98
31	1118061.1	Alistipes obesi	Bacteria	62.42
32	1871035.1	Ruminococcus sp. Marseille-P3213 sp. Marseille-P3213	Bacteria	61.52
33	214856.1	Alistipes finegoldii strain 2789STDY5608890	Bacteria	61.03
34	853.3	Faecalibacterium prausnitzii strain 2789STDY5608869	Bacteria	59.87
35	1033732	Alistipes senegalensis JC50	Bacteria	59.30
36	1561.2	Clostridium baratii strain 2789STDY5834907	Bacteria	57.98
37	1203611	Alistipes onderdonkii WAL 8169 = DSM 19147	Bacteria	57.58
38	1121130	Butyricimonas virosa DSM 23226	Bacteria	57.46
39	679935	Alistipes finegoldii DSM 17242	Bacteria	56.98
40	166486.1	Roseburia intestinalis strain 2789STDY5834960	Bacteria	56.66
41	411460	Ruminococcus torques ATCC 27756	Bacteria	55.74

42	1917876.1	Blautia sp. Marseille-P3087 sp. Marseille-P3087	Bacteria	54.52
43	1673721.1	Intestinimonas massiliensis sp. GD2	Bacteria	54.41
44	552398.1	Ruminococcaceae bacterium D16	Bacteria	53.64
45	1841867.1	Phocea massiliensis strain Marseille-P2769	Bacteria	53.62
46	999420	Parabacteroides merdae CL03T12C32	Bacteria	53.57
47	360807.3	Roseburia inulinivorans strain 2789STDY5608887	Bacteria	53.37
48	742725	Alistipes indistinctus YIT 12060	Bacteria	52.74
49	1232459.1	Oscillospiraceae bacterium VE202-24	Bacteria	52.59
50	1160721.1	Ruminococcus bicirculans	Bacteria	52.44
51	411903	Collinsella aerofaciens ATCC 25986	Bacteria	52.05
52	88431.4	Dorea longicatena strain 2789STDY5834914	Bacteria	52.01
53	742726	Barnesiella intestinihominis YIT 11860	Bacteria	50.96
54	46506.1	Bacteroides stercoris strain CL09T03C01	Bacteria	50.29
55	469610.1	Burkholderiales bacterium 1_1_47	Bacteria	50.25
56	301302.1	Roseburia faecis	Bacteria	50.11
57	1121098	Bacteroides massiliensis B84634 = Timone 84634 = DSM 17679 = JCM 13223	Bacteria	49.60
58	1339345	Parabacteroides distasonis str. 3999B T(B) 6	Bacteria	49.53
59	428126	[Clostridium] spiroforme DSM 1552	Bacteria	49.02
60	1235786	Bacteroides vulgatus dnLKV7	Bacteria	48.68
61	411463	Eubacterium ventriosum ATCC 27560	Bacteria	47.90
62	762966	Parasutterella excrementihominis YIT 11859	Bacteria	47.89
63	1095771.1	Ruminococcus sp. JC304	Bacteria	47.14
64	1235787	Bacteroides uniformis dnLKV2	Bacteria	46.81
65	536231	Roseburia intestinalis L1-82	Bacteria	46.72
66	665949.1	Tannerella sp. 6_1_58FAA_CT1	Bacteria	45.53
67	301302.2	Roseburia faecis strain 2789STDY5608863	Bacteria	45.20
68	411470	Ruminococcus gnavus ATCC 29149	Bacteria	44.60
69	411461	Dorea formicigenerans ATCC 27755	Bacteria	43.73
70	853.2	Faecalibacterium prausnitzii strain 2789STDY5834930	Bacteria	43.69
71	997877	Bacteroides dorei CL03T12C01	Bacteria	43.66
72	665956.1	Subdoligranulum sp. 4_3_54A2FAA	Bacteria	43.50
73	1211813	Alistipes ihumii AP11	Bacteria	43.27
74	1703332.1	Lachnospiraceae bacterium TF01-11	Bacteria	43.12
75	39492.1	[Eubacterium] siraeum strain 2789STDY5834928	Bacteria	42.96
76	908612.1	Alistipes sp. HGB5	Bacteria	42.46
77	1504823.1	bacterium LF-3	Bacteria	42.43
78	411467	Pseudoflavonifractor capillosus ATCC 29799	Bacteria	41.18
79	1073351	Bacteroides stercoris CC31F	Bacteria	40.91
80	214856.2	Alistipes finegoldii strain 2789STDY5834947	Bacteria	40.89
81	1150298.2	Fusicatenibacter saccharivorans strain 2789STDY5834885	Bacteria	40.87
82	1150298.1	Fusicatenibacter saccharivorans strain 2789STDY5608849	Bacteria	40.83
83	450746.1	Coprobacillus sp. 8_1_38FAA	Bacteria	40.46
84	471875	Ruminococcus lactaris ATCC 29176	Bacteria	40.21
85	39485.1	[Eubacterium] eligens strain 2789STDY5834878	Bacteria	39.69

86	658087.1	Lachnospiraceae bacterium 7_1_58FAA	Bacteria	39.14
87	39491.2	[Eubacterium] rectale strain 2789STDY5834968	Bacteria	39.06
88	483215	Bacteroides finegoldii DSM 17565	Bacteria	38.99
89	1297617.1	Intestinimonas butyriciproducens strain AF211	Bacteria	38.89
90	545696	Holdemania filiformis DSM 12042	Bacteria	38.37
91	1720200.1	Anaerotruncus rubiinfantis sp. MT15	Bacteria	38.13
92	562.665	Escherichia coli isolate 15	Bacteria	37.84
93	411486.1	Clostridium sp. M62/1	Bacteria	37.66
94	1499682.1	Alistipes sp. AL-1	Bacteria	37.07
95	585543.1	Bacteroides sp. D20	Bacteria	36.99
96	820.3	Bacteroides uniformis strain 2789STDY5834847	Bacteria	36.89
97	997891	Bacteroides vulgatus CL09T03C04	Bacteria	36.83
98	1232439.1	Clostridiales bacterium VE202-03	Bacteria	36.69
99	820.5	Bacteroides uniformis strain 2789STDY5608791	Bacteria	36.47
100	1121115	Blautia wexlerae DSM 19850	Bacteria	36.26
101	1150298.3	Fusicatenibacter saccharivorans strain 2789STDY5834923	Bacteria	36.13
102	1720194.1	Clostridium sp. AT4 sp. AT5	Bacteria	35.76
103	47678.2	Bacteroides caccae strain 2789STDY5834880	Bacteria	35.52
104	820.9	Bacteroides uniformis strain KLE1607	Bacteria	35.44
105	515619	[Eubacterium rectale] ATCC 33656	Bacteria	34.82
106	435590	Bacteroides vulgatus ATCC 8482	Bacteria	34.72
107	418240.2	Blautia wexlerae strain 2789STDY5834911	Bacteria	34.25
108	1750560.1	Parabacteroides sp. SN4 strain SN4, sp. SB4	Bacteria	34.16
109	1739298.1	Bacteroides sp. HMSC067B03	Bacteria	33.55
110	1232453.1	Clostridiales bacterium VE202-21	Bacteria	33.12
111	428128	[Eubacterium] siraeum DSM 15702	Bacteria	33.09
112	1232438.1	Clostridiales bacterium VE202-01	Bacteria	33.00
113	649724.1	Clostridium sp. ATCC BAA-442	Bacteria	32.75
114	762984	Bacteroides clarus YIT 12056	Bacteria	32.70
115	329854.2	Bacteroides intestinalis strain KLE1704	Bacteria	32.24
116	411901	Bacteroides caccae ATCC 43185	Bacteria	32.09
117	820.1	Bacteroides uniformis	Bacteria	31.96
118	411462	Dorea longicatena DSM 13814	Bacteria	31.84
119	1697793.1	Clostridia bacterium UC5.1-1E11	Bacteria	31.65
120	742722.1	Collinsella sp. 4_8_47FAA	Bacteria	31.64
121	821.2	Bacteroides vulgatus strain 2789STDY5834842	Bacteria	31.63
122	40520.1	Blautia obeum strain 2789STDY5834921	Bacteria	31.62
123	1432052.6	Eisenbergiella tayi strain NML150140-1	Bacteria	31.17
124	537012	Bacteroides cellulosilyticus DSM 14838	Bacteria	30.84
125	562983	Gemella sanguinis M325	Bacteria	30.79
126	1280698	Dorea longicatena AGR2136	Bacteria	30.28
127	457412.1	Ruminococcus sp. 5_1_39BFAA	Bacteria	29.93
128	762968	Paraprevotella clara YIT 11840	Bacteria	29.81
129	39488.2	[Eubacterium] hallii strain 2789STDY5834966	Bacteria	29.13
130	470146	Coprococcus comes ATCC 27758	Bacteria	28.79
131	702450	Turicibacter sanguinis PC909	Bacteria	28.77

132	1871020.1	Clostridium sp. Marseille-P3244 sp. Marseille-P3244	Bacteria	28.52
133	515620	[Eubacterium] eligens ATCC 27750	Bacteria	28.51
134	1871018.1	Angelakisella massiliensis strain Marseille-P3217	Bacteria	28.49
135	1852384.1	Ruminococcaceae bacterium Marseille-P2963	Bacteria	28.31
136	742821	Sutterella wadsworthensis 3_1_45B	Bacteria	27.91
137	40520.5	Blautia obeum strain 2789STDY5834957	Bacteria	27.87
138	762982	Paraprevotella xylaniphila YIT 11841	Bacteria	27.66
139	1776382.1	Neglecta timonensis strain SN17	Bacteria	27.37
140	622312	Roseburia inulinivorans DSM 16841	Bacteria	27.10
141	997873	Bacteroides caccae CL03T12C61	Bacteria	27.05
142	411473	Ruminococcus callidus ATCC 27760	Bacteria	27.03
143	33039.3	[Ruminococcus] torques strain 2789STDY5608867	Bacteria	26.72
144	1211417.1	uncultured phage crAssphage	Viruses	26.64
145	360807.2	Roseburia inulinivorans strain 2789STDY5608835	Bacteria	26.64
146	1310661	Acinetobacter baumannii 855125	Bacteria	26.25
147	1073376	Ruminococcus lactaris CC59_002D	Bacteria	26.25
148	483217	Bacteroides dorei DSM 17855	Bacteria	26.24
149	33043.4	Coprococcus eutactus strain 2789STDY5608829	Bacteria	26.20
150	1870991.1	Massilioclostridium coli strain Marseille-P2976	Bacteria	26.16
151	33035.1	Blautia producta strain ER3	Bacteria	26.09
152	1776384.1	Emergencia timonensis strain SN18	Bacteria	26.09
153	1203465	Bacteroides timonensis AP1	Bacteria	25.91
154	445971	Anaerofustis stercorihominis DSM 17244	Bacteria	25.70
155	1352.143	Enterococcus faecium isolate Hp_74-d6	Bacteria	25.50
156	537006	Parabacteroides johnsonii DSM 18315	Bacteria	25.49
157	457389.1	Bacteroides sp. 3_1_13	Bacteria	25.47
158	1816676.1	Alistipes sp. Marseille-P2431 sp. Marseille-P2431	Bacteria	25.09
159	1163670.1	Bacteroides sp. 14(A)	Bacteria	25.06
160	39491.3	[Eubacterium] rectale strain 2789STDY5608860	Bacteria	25.02
161	1627893.1	Ruminococcaceae bacterium cv2	Bacteria	24.98
162	470145	Bacteroides coprocola DSM 17136	Bacteria	24.89
163	469592.1	Bacteroides sp. 3_1_19	Bacteria	24.80
164	821.3	Bacteroides vulgatus strain 2789STDY5834944	Bacteria	24.79
165	1408437	Butyricicoccus desmolans ATCC 43058	Bacteria	24.66
166	100886.1	Catenibacterium mitsuokai strain 2789STDY5608825	Bacteria	24.64
167	360807.1	Roseburia inulinivorans	Bacteria	24.53
168	537011	Prevotella copri DSM 18205	Bacteria	24.34
169	658662.1	Parabacteroides sp. D26	Bacteria	24.33
170	665953	Bacteroides eggerthii 1_2_48FAA	Bacteria	24.29
171	999421	Parabacteroides merdae CL09T00C40	Bacteria	24.19
172	997888	Bacteroides finegoldii CL09T03C10	Bacteria	24.06
173	511680	Butyrivibrio crossotus DSM 2876	Bacteria	23.93
174	563193.1	Parabacteroides sp. D13	Bacteria	23.81
175	411468	[Clostridium] scindens ATCC 35704	Bacteria	23.75
176	818.7	Bacteroides thetaiotaomicron strain 14-106904-2	Bacteria	23.72

177	649756.2	Anaerostipes hadrus strain 2789STDY5608830	Bacteria	23.70
178	1852366.1	Holdemania sp. Marseille-P2844 sp. Marseille-P2844	Bacteria	23.65
179	1697787.1	Clostridia bacterium UC5.1-1D10	Bacteria	23.58
180	626939	Phascolarctobacterium succinatutens YIT 12067	Bacteria	23.55
181	1352.158	Enterococcus faecium isolate Hp_23-14	Bacteria	23.48
182	1121323	[Clostridium] lactatifermentans DSM 14214	Bacteria	23.19
183	74426.1	Collinsella aerofaciens strain 2789STDY5834902	Bacteria	23.12
184	329854.1	Bacteroides intestinalis	Bacteria	22.63
185	1122155	Lactonifactor longoviformis DSM 17459	Bacteria	22.62
186	820.2	Bacteroides uniformis strain 2789STDY5834942	Bacteria	22.50
187	471870	Bacteroides intestinalis DSM 17393	Bacteria	22.48
188	1203554	Sutterella wadsworthensis HGA0223	Bacteria	21.95
189	518637	Holdemanella biformis DSM 3989	Bacteria	21.77
190	1339352	Bacteroides vulgatus str. 3975 RP4	Bacteria	21.76
191	410072.2	Coprococcus comes strain 2789STDY5834962	Bacteria	21.75
192	556260	Bacteroides dorei 5_1_36/D4	Bacteria	21.34
193	556259.1	Bacteroides sp. D2	Bacteria	21.34
194	40520.2	Blautia obeum strain 2789STDY5834861	Bacteria	21.30
195	821.5	Bacteroides vulgatus strain mpk	Bacteria	21.03
196	936548.1	Actinomyces sp. ICM47	Bacteria	20.98
197	820.6	Bacteroides uniformis strain 2789STDY5834898	Bacteria	20.79
198	28052.2	Lachnospira pectinoschiza strain 2789STDY5834836	Bacteria	20.70
199	1339335	Bacteroides fragilis str. 3-F-2 #6	Bacteria	20.57
200	1226324.1	Blautia sp. KLE 1732	Bacteria	20.41
201	566550	Hungatella hathewayi DSM 13479	Bacteria	20.30
202	338188.1	Bacteroides finegoldii strain 2789STDY5608840	Bacteria	20.26
203	457393.1	Bacteroides sp. 4_1_36	Bacteria	20.16
204	592028	Dialister invisus DSM 15470	Bacteria	20.07
205	40520.4	Blautia obeum strain 2789STDY5608837	Bacteria	19.99
206	821.1	Bacteroides vulgatus strain 2789STDY5834897	Bacteria	19.95
207	1034345	Senegalimassilia anaerobia JC110	Bacteria	19.53
208	239935.2	Akkermansia muciniphila strain YL44	Bacteria	19.20
209	823.1	Parabacteroides distasonis strain 2789STDY5608872	Bacteria	19.04
210	457395.1	Bacteroides sp. 9_1_42FAA	Bacteria	19.00
211	28052.1	Lachnospira pectinoschiza strain 2789STDY5834886	Bacteria	18.95
212	1211819	Holdemania massiliensis AP2	Bacteria	18.88
213	1033731	Alistipes timonensis JC136	Bacteria	18.86
214	411489.1	Clostridium sp. L2-50	Bacteria	18.85
215	999419	Parabacteroides johnsonii CL02T12C29	Bacteria	18.81
216	742823	Sutterella wadsworthensis 2_1_59BFAA	Bacteria	18.73
217	40520.3	Blautia obeum strain 2789STDY5608838	Bacteria	18.70
218	33039.4	[Ruminococcus] torques strain 2789STDY5834841	Bacteria	18.67
219	74426.3	Collinsella aerofaciens strain 2789STDY5608823	Bacteria	18.63

220	702443	Bacteroides ovatus SD CMC 3f	Bacteria	18.43
221	1550024.2	Ruthenibacterium lactatiformans strain 585-1	Bacteria	18.36
222	1720300.1	Ruminococcus sp. AT10	Bacteria	18.33
223	1121129	Butyricimonas synergistica DSM 23225	Bacteria	18.19
224	457394.1	Bacteroides sp. 4_3_47FAA	Bacteria	18.14
225	823.4	Parabacteroides distasonis strain 2789STDY5834901	Bacteria	17.92
226	693988.1	Bilophila sp. 4_1_30	Bacteria	17.92
227	1280691	Blautia wexlerae AGR2146	Bacteria	17.69
228	1226325.1	Clostridium sp. KLE 1755	Bacteria	17.61
229	39491.4	[Eubacterium] rectale strain 2789STDY5834884	Bacteria	17.59
230	1121096	Bacteroides gallinarum DSM 18171 = JCM 13658	Bacteria	17.53
231	537013	[Clostridium] methylpentosum DSM 5476	Bacteria	17.24
232	1574262.1	Sutterella sp. KLE1602	Bacteria	17.22
233	820.7	Bacteroides uniformis strain 2789STDY5608864	Bacteria	17.11
234	411490	Anaerostipes caccae DSM 14662	Bacteria	17.09
235	1870993.1	Tyzzerella sp. Marseille-P3062 sp. Marseille-P3062	Bacteria	16.92
236	483216	Bacteroides eggerthii DSM 20697	Bacteria	16.85
237	1739319.1	Bacteroides sp. HMSC068A09	Bacteria	16.67
238	563192	Bilophila wadsworthia 3_1_6	Bacteria	16.53
239	1123075	Ruminococcus gauvreauii DSM 19829	Bacteria	16.49
240	1078089.1	Bacteroides sp. HPS0048	Bacteria	16.46
241	1280669.1	Dorea sp. AGR2135	Bacteria	16.34
242	1203608	Bacteroides nordii WAL 11050 = JCM 12987	Bacteria	16.27
243	371601.1	Bacteroides xylanisolvens strain 2789STDY5608839	Bacteria	16.25
244	665954	Bacteroides ovatus 3_8_47FAA	Bacteria	15.92
245	33039.2	[Ruminococcus] torques strain 2789STDY5608833	Bacteria	15.88
246	742765	Dorea formicigenerans 4_6_53AFAA	Bacteria	15.76
247	457390.1	Bacteroides sp. 3_1_23	Bacteria	15.65
248	418240.1	Blautia wexlerae strain 2789STDY5834863	Bacteria	15.65
249	47678.1	Bacteroides caccae strain 2789STDY5834946	Bacteria	15.60
250	1432052.1	Eisenbergiella tayi strain NML	Bacteria	15.53
251	1232443.1	Clostridiales bacterium VE202-13	Bacteria	15.53
252	1232457.1	Clostridiales bacterium VE202-27	Bacteria	15.39
253	1653435.1	Clostridium sp. BR31	Bacteria	15.29
254	818.2	Bacteroides thetaiotaomicron strain 19_BTHE	Bacteria	15.29
255	1256908	Eubacterium ramulus ATCC 29099	Bacteria	15.25
256	823.3	Parabacteroides distasonis strain 2789STDY5608822	Bacteria	15.17
257	1235785	Bacteroides thetaiotaomicron dnLKV9	Bacteria	15.12
258	411474	Coprococcus eutactus ATCC 27759	Bacteria	15.07
259	457391.1	Bacteroides sp. 3_1_33FAA	Bacteria	14.96
260	823.2	Parabacteroides distasonis strain 2789STDY5834948	Bacteria	14.87
261	457415.1	Synergistes sp. 3_1_syn1	Bacteria	14.80

262	349741	Akkermansia muciniphila ATCC BAA-835	Bacteria	14.79
263	1235788	Bacteroides massiliensis dnLKV3	Bacteria	14.78
264	997892	Bacteroides xylanisolvens CL03T12C04	Bacteria	14.76
265	818.5	Bacteroides thetaiotaomicron strain 2789STDY5834945	Bacteria	14.69
266	1339314	Bacteroides fragilis str. 3976T8	Bacteria	14.56
267	246787.1	Bacteroides cellulosilyticus strain CL09T06C25	Bacteria	14.53
268	500632	Tyzzerella nexilis DSM 1787	Bacteria	14.51
269	911128	Methanobrevibacter smithii TS94C	Archaea	14.47
270	658655.1	Lachnospiraceae bacterium 1_4_56FAA	Bacteria	14.41
271	818.1	Bacteroides thetaiotaomicron strain 7330	Bacteria	14.38
272	469590.1	Bacteroides sp. 2_2_4	Bacteria	14.28
273	484018	Bacteroides plebeius DSM 17135	Bacteria	14.17
274	1077285	Bacteroides faecis MAJ27	Bacteria	14.11
275	1650663.1	Fournierella massiliensis strain AM2	Bacteria	14.03
276	649756.3	Anaerostipes hadrus strain 2789STDY5834860	Bacteria	14.03
277	1297617.2	Intestinimonas butyriciproducens strain 27-5-10	Bacteria	14.02
278	88431.3	Dorea longicatena strain 2789STDY5608866	Bacteria	13.99
279	997884	Bacteroides nordii CL02T12C05	Bacteria	13.95
280	910311.1	Eggerthella sp. HGA1	Bacteria	13.94
281	483218	[Bacteroides] pectinophilus ATCC 43243	Bacteria	13.93
282	1234889	Leuconostoc mesenteroides subsp. cremoris TIFN8	Bacteria	13.93
283	1207542	Bifidobacterium bifidum LMG 13195	Bacteria	13.92
284	997874	Bacteroides cellulosilyticus CL02T12C19	Bacteria	13.65
285	476272	Blautia hydrogenotrophica DSM 10507	Bacteria	13.34
286	818.1	Bacteroides thetaiotaomicron	Bacteria	13.27
287	1002367	Prevotella stercorea DSM 18206	Bacteria	13.15
288	1841855.1	Bacteroides sp. Marseille-P2653 sp. Marseille-P2653	Bacteria	12.94
289	1298596	Ruminococcus faecis JCM 15917	Bacteria	12.86
290	1574263.1	Candidatus Stoquefichus sp. KLE1796	Bacteria	12.66
291	1745713.1	Bariatricus massiliensis strain AT12	Bacteria	12.58
292	1157708	Variovorax paradoxus 110B	Bacteria	12.53
293	1310866	Acinetobacter baumannii 25307_2	Bacteria	12.52
294	999416	Parabacteroides distasonis CL03T12C09	Bacteria	12.45
295	1349822	Coprobacter fastidiosus NSB1	Bacteria	12.41
296	469589.1	Bacteroides sp. 2_1_33B	Bacteria	12.40
297	1121101	Bacteroides salyersiae WAL 10018 = DSM 18765 = JCM 12988	Bacteria	12.14
298	435591	Parabacteroides distasonis ATCC 8503	Bacteria	11.85
299	411475	Flavonifractor plautii ATCC 29863	Bacteria	11.78
300	818.6	Bacteroides thetaiotaomicron strain 2789STDY5834899	Bacteria	11.77
301	1907659.1	Blautia sp. Marseille-P3201T strain Marseille-P3201	Bacteria	11.75
302	28116.7	Bacteroides ovatus strain KLE1656	Bacteria	11.74
303	1550024.1	Ruthenibacterium lactatiformans strain 668	Bacteria	11.72

304	1852361.1	Actinomyces sp. Marseille-P2825 sp. Marseille-P2825	Bacteria	11.69
305	1034346.1	Dielma fastidiosa	Bacteria	11.66
306	1805476.1	Blautia sp. Marseille-P2398	Bacteria	11.64
307	821.4	Bacteroides vulgatus strain NLAE-zl-G202	Bacteria	11.61
308	1796613.1	Bacteroides caecimuris strain I48	Bacteria	11.61
309	371601.3	Bacteroides xylanisolvens strain NLAE-zl-G339	Bacteria	11.60
310	169435.1	Anaerotruncus colihominis strain 2789STDY5834939	Bacteria	11.44
311	1151410	Clostridioides difficile P28	Bacteria	11.42
312	154046.2	Hungatella hathewayi strain 2789STDY5834916	Bacteria	11.41
313	818.9	Bacteroides thetaiotaomicron strain KPPR-3	Bacteria	11.36
314	818.4	Bacteroides thetaiotaomicron strain 2789STDY5608873	Bacteria	11.27
315	518636	[Clostridium asparagiforme] DSM 15981	Bacteria	11.22
316	999418	Parabacteroides goldsteinii CL02T12C30	Bacteria	11.15
317	556269	Oxalobacter formigenes OXCC13	Bacteria	11.15
318	702446	Bacteroides vulgatus PC510	Bacteria	11.15
319	1352.147	Enterococcus faecium isolate Hp_7-6	Bacteria	10.88
320	469586.1	Bacteroides sp. 1_1_6	Bacteria	10.87
321	1227261	Actinobaculum sp. oral taxon 183 str. F0552	Bacteria	10.87
322	28116.5	Bacteroides ovatus strain NLAE-zl-C500	Bacteria	10.82
323	47770.1	Lactobacillus crispatus strain C25	Bacteria	10.74
324	658659.1	Erysipelotrichaceae bacterium 3_1_53	Bacteria	10.61
325	154046.1	Hungatella hathewayi strain 2789STDY5608850	Bacteria	10.54
326	820.4	Bacteroides uniformis strain 2789STDY5834844	Bacteria	10.53
327	1121333	[Clostridium] saccharogumia DSM 17460	Bacteria	10.46
328	213810	Ruminococcus champanellensis 18P13 = JCM 17042	Bacteria	10.40
329	33043.2	Coprococcus eutactus strain 2789STDY5608888	Bacteria	10.38
330	521003	Collinsella intestinalis DSM 13280	Bacteria	10.38
331	292800.2	Flavonifractor plautii strain 2789STDY5834932	Bacteria	10.31
332	742738	Clostridium orbiscindens 1_3_50AFAA	Bacteria	10.24
333	469614.1	Erysipelotrichaceae bacterium 6_1_45	Bacteria	10.20
334	626937.1	Christensenella minuta strain DSM	Bacteria	10.17
335	469593.1	Bacteroides sp. 3_1_40A	Bacteria	10.11
336	1076696	Entamoeba nuttalli P19	Eukaryota	9.85
337	649742	Actinomyces odontolyticus F0309	Bacteria	9.84
338	823.5	Parabacteroides distasonis	Bacteria	9.80
339	33043.3	Coprococcus eutactus strain 2789STDY5608843	Bacteria	9.73
340	33038.1	[Ruminococcus] gnavus	Bacteria	9.69
341	457387.1	Bacteroides sp. 1_1_30	Bacteria	9.63
342	562.2335	Escherichia coli strain LS5218	Bacteria	9.53
343	469591.1	Parabacteroides sp. 20_3	Bacteria	9.47
344	88431.1	Dorea longicatena strain 2789STDY5834961	Bacteria	9.43
345	1297617.3	Intestinimonas butyriciproducens strain ER1	Bacteria	9.30
346	291644.1	Bacteroides salyersiae strain 2789STDY5608871	Bacteria	9.22
347	74426.2	Collinsella aerofaciens strain 2789STDY5608842	Bacteria	9.19

348	763034	Bacteroides fluxus YIT 12057	Bacteria	9.15
349	88431.2	Dorea longicatena strain 2789STDY5608851	Bacteria	9.03
350	1188792.1	Phaseolus vulgaris endornavirus 1	Viruses	8.91
351	1852370.1	Prevotellamassilia timonensis strain Marseille-P2831	Bacteria	8.82
352	33039.1	[Ruminococcus] torques strain 2789STDY5834889	Bacteria	8.75
353	649756.6	Anaerostipes hadrus strain 2789STDY5834959	Bacteria	8.74
354	1871006.1	Bacteroides sp. Marseille-P3132 sp. Marseille-P3132	Bacteria	8.70
355	1776379.1	Prevotella sp. KHD1 sp. KHD1	Bacteria	8.67
356	817.2	Bacteroides fragilis strain 14-106904-1	Bacteria	8.57
357	818.11	Bacteroides thetaiotaomicron strain KLE1254	Bacteria	8.53
358	553973	[Clostridium] hylemonae DSM 15053	Bacteria	8.43
359	818.8	Bacteroides thetaiotaomicron isolate 3731	Bacteria	8.36
360	1871021.1	Lachnoclostridium phocaeense strain Marseille-P3177T sp. Marseille-P3177	Bacteria	8.22
361	658089.1	Lachnospiraceae bacterium 5_1_63FAA	Bacteria	8.21
362	410072.1	Coprococcus comes strain 2789STDY5608832	Bacteria	8.15
363	649756.1	Anaerostipes hadrus strain PEL	Bacteria	7.98
364	1506471.1	Sutterellaceae bacterium ND3	Bacteria	7.96
365	226186	Bacteroides thetaiotaomicron VPI-5482	Bacteria	7.91
366	742727	Bacteroides oleiciplenus YIT 12058	Bacteria	7.82
367	658086.1	Lachnospiraceae bacterium 3_1_57FAA_CT1	Bacteria	7.80
368	888727	Eubacterium sulci ATCC 35585	Bacteria	7.79
369	649756.7	Anaerostipes hadrus strain BPB5	Bacteria	7.78
370	411464	Desulfovibrio piger ATCC 29098	Bacteria	7.76
371	1392862	Escherichia coli M17	Bacteria	7.73
372	1841856.1	Bacteroides mediterraneensis strain Marseille-P2644	Bacteria	7.72
373	1232444.1	Clostridiales bacterium VE202-15	Bacteria	7.62
374	876.1	Desulfovibrio desulfuricans strain DSM	Bacteria	7.57
375	292800.1	Flavonifractor plautii strain 2789STDY5834892	Bacteria	7.54
376	428127	[Eubacterium] dolichum DSM 3991	Bacteria	7.51
377	665950.1	Lachnospiraceae bacterium 3_1_46FAA	Bacteria	7.47
378	1852381.1	Sutterellaceae bacterium Marseille-P2968	Bacteria	7.46
379	545697	Clostridium celatum DSM 1785	Bacteria	7.43
380	457421.1	Clostridiales bacterium 1_7_47FAA	Bacteria	7.39
381	46503.1	Parabacteroides merdae strain 2789STDY5834848	Bacteria	7.28
382	1339276	Bacteroides fragilis str. DS-208	Bacteria	7.26
383	1903263.1	Traorella massiliensis strain Marseille-P3110	Bacteria	7.21
384	85831.1	Bacteroides acidifaciens	Bacteria	7.15
385	12239.2	Pepper mild mottle virus	Viruses	7.14
386	997887	Bacteroides salyersiae CL02T12C01	Bacteria	7.12
387	649756.4	Anaerostipes hadrus strain 2789STDY5834908	Bacteria	7.12
388	1917883.1	Bacteroides sp. Marseille-P3166 sp. Marseille-P3166	Bacteria	7.09

389	410072.3	Coprococcus comes strain 2789STDY5834866	Bacteria	7.05
390	999417	Parabacteroides distasonis CL09T03C24	Bacteria	6.99
391	1121445	Desulfovibrio desulfuricans subsp. desulfuricans DSM 642	Bacteria	6.92
392	1834205.1	Burkholderiales bacterium YL45	Bacteria	6.80
393	742818	Slackia piriformis YIT 12062	Bacteria	6.79
394	1339283	Bacteroides fragilis str. 3996 N(B) 6	Bacteria	6.64
395	1547597.1	Sanguibacteroides justesenii strain OUH	Bacteria	6.58
396	938289.1	Levyella massiliensis	Bacteria	6.55
397	1871013.1	Parabacteroides sp. Marseille-P3236 strain Marseille-P3236, sp. Marseille-P3136	Bacteria	6.54
398	457397.1	Clostridium sp. 1_1_41A1FAA	Bacteria	6.52
399	1339271	Bacteroides fragilis str. J-143-4	Bacteria	6.47
400	457402.1	Eubacterium sp. 3_1_31	Bacteria	6.46
401	1115692.1	Cannabis cryptic virus isolate hemp09	Viruses	6.43
402	1339298	Bacteroides fragilis str. 3725 D9(v)	Bacteria	6.42
403	742817	Odoribacter laneus YIT 12061	Bacteria	6.38
404	927665	Parabacteroides goldsteinii DSM 19448 = WAL 12034	Bacteria	6.38
405	12968.1	Blastocystis hominis isolate B	Eukaryota	6.35
406	1676614.1	Prevotella sp. 109	Bacteria	6.31
407	1291051	[Clostridium] glycyrrhizinilyticum JCM 13369	Bacteria	6.23
408	585544.1	Bacteroides sp. D22	Bacteria	6.21
409	1499681.1	Collinsella sp. MS5	Bacteria	6.13
410	12172.2	Shallot latent virus isolate MS/SW/Aus2	Viruses	6.12
411	1602172.1	Prevotella sp. P5-125	Bacteria	6.11
412	1697795.1	Clostridia bacterium UC5.1-2H11	Bacteria	6.11
413	817.3	Bacteroides fragilis strain DCMOUH0085B	Bacteria	6.09
414	410072.4	Coprococcus comes strain 2789STDY5834913	Bacteria	6.08
415	649756.5	Anaerostipes hadrus strain 2789STDY5608868	Bacteria	6.08
416	186772.1	Saccharomyces 20S RNA narnavirus	Viruses	6.07
417	28116.4	Bacteroides ovatus strain NLAE-zl-C57	Bacteria	5.92
418	360107	Campylobacter hominis ATCC BAA-381	Bacteria	5.90
419	1284708.1	Tissierellia bacterium S7-1-4	Bacteria	5.87
420	328812.1	Parabacteroides goldsteinii strain 910340	Bacteria	5.77
421	154288.3	Turicibacter sanguinis strain 2789STDY5608865	Bacteria	5.70
422	1579343.1	Streptococcus sp. 263_SSPC	Bacteria	5.56
423	1078087.1	Parabacteroides sp. HGS0025	Bacteria	5.56
424	362693.1	Oryza sativa endornavirus	Viruses	5.53
425	1816678.1	Christensenella timonensis strain Marseille-P2437	Bacteria	5.40
426	997885	Bacteroides ovatus CL02T12C04	Bacteria	5.38
427	817.11	Bacteroides fragilis strain 20656-2-1	Bacteria	5.38
428	33043.1	Coprococcus eutactus strain 2789STDY5834963	Bacteria	5.37
429	658085.1	Lachnospiraceae bacterium 5_1_57FAA	Bacteria	5.34
430	901.1	Desulfovibrio piger isolate DESPIGER1	Bacteria	5.34
431	435830	Actinomyces graevenitzii C83	Bacteria	5.24
432	665940.1	Clostridium sp. 7_3_54FAA	Bacteria	5.22
433	479437	Eggerthella lenta DSM 2243	Bacteria	5.14

434	1295009	Candidatus Methanomassiliicoccus intestinalis Issoire-Mx1	Archaea	5.14
435	999408	[Clostridium] clostridioforme 90A8	Bacteria	5.13
436	1227262	Actinomyces johnsonii F0510	Bacteria	5.13
437	944170.2	Blastocystis sp. subtype 4 strain WR1	Eukaryota	5.06
438	451640	Catenibacterium mitsuokai DSM 15897	Bacteria	5.01
439	1459804	Finegoldia magna ALB8	Bacteria	4.91
440	1121114	Blautia producta ATCC 27340 = DSM 2950	Bacteria	4.83
441	1658112.1	Eubacterium sp. SB2	Bacteria	4.79
442	1339348	Bacteroides uniformis str. 3978 T3 i	Bacteria	4.79
443	997886	Bacteroides ovatus CL03T12C18	Bacteria	4.68
444	742743	Dialister succinatiphilus YIT 11850	Bacteria	4.65
445	1123263	Solobacterium moorei DSM 22971	Bacteria	4.61
446	28116.2	Bacteroides ovatus strain 2789STDY5834943	Bacteria	4.55
447	1339344	Parabacteroides distasonis str. 3999B T(B) 4	Bacteria	4.45
448	290052.1	Acetivibrio ethanolgignens strain ACET-33324	Bacteria	4.44
449	1432052.7	Eisenbergiella tayi strain DSM	Bacteria	4.34
450	592026	Catonella morbi ATCC 51271	Bacteria	4.26
451	1120941	Actinomyces dentalis DSM 19115	Bacteria	4.24
452	1519438.1	Eubacterium sp. ER2	Bacteria	4.21
453	327387.1	Tropical soda apple mosaic virus isolate Okeechobee	Viruses	4.17
454	445973	Intestinibacter bartlettii DSM 16795	Bacteria	4.14
455	239935.1	Akkermansia muciniphila isolate Urmite	Bacteria	4.13
456	817.19	Bacteroides fragilis strain DCMOUH0042B	Bacteria	4.10
457	658656.1	Lachnospiraceae bacterium 6_1_37FAA	Bacteria	4.07
458	1841865.1	Mediterranea massiliensis strain Marseille-P2645	Bacteria	4.05
459	292800.3	Flavonifractor plautii strain YL31	Bacteria	4.04
460	1761477.1	Tomato brown rugose fruit virus isolate Tom1-Jo	Viruses	4.03
461	1871003.1	Tidjanibacter massiliensis strain Marseille-P3084	Bacteria	4.02
462	1349763	Curvibacter delicatus NBRC 14919	Bacteria	4.02
463	547042	Bacteroides coprophilus DSM 18228 = JCM 13818	Bacteria	4.00
464	1671366.1	Ruminococcus sp. DSM 100440	Bacteria	3.97
465	371601.2	Bacteroides xylanisolvens strain NLAE-zl-C202	Bacteria	3.94
466	936595.1	Lachnoanaerobaculum sp. OBRC5-5	Bacteria	3.91
467	1723384.1	Fenollaria timonensis	Bacteria	3.91
468	667015	Bacteroides salanitronis DSM 18170	Bacteria	3.86
469	658657.1	Erysipelotrichaceae bacterium 21_3	Bacteria	3.86
470	1531.1	[Clostridium] clostridioforme strain 2789STDY5834865	Bacteria	3.85
471	457424	Bacteroides fragilis 3_1_12	Bacteria	3.83
472	1871030.1	Merdibacter massiliensis strain Marseille-P3254	Bacteria	3.80
473	742742	Collinsella tanakaei YIT 12063	Bacteria	3.79
474	1423800	Lactobacillus sanfranciscensis DSM 20451	Bacteria	3.75
475	1501392.1	Coprobacter secundus strain 177	Bacteria	3.65
476	712411.1	Olsenella sp. oral taxon 807 strain F0089	Bacteria	3.53
477	1133596	Pediococcus pentosaceus IE-3	Bacteria	3.50

478	1816694.1	Clostridium sp. Marseille-P2538 sp. Marseille-P2538	Bacteria	3.48
479	261299.1	Intestinibacter bartlettii strain 2789STDY5834879	Bacteria	3.48
480	880074	Barnesiella viscericola DSM 18177	Bacteria	3.46
481	1197717.1	Cloacibacillus porcorum strain CL-84	Bacteria	3.44
482	1602168.1	Prevotella sp. P4-65	Bacteria	3.42
483	28116.1	Bacteroides ovatus strain CL09T03C03	Bacteria	3.38
484	999407	[Clostridium] clostridioforme 90A7	Bacteria	3.32
485	1261635.1	Roseburia sp. 831b	Bacteria	3.28
486	717960	Faecalitalea cylindroides T2-87	Bacteria	3.25
487	1121094	Bacteroides barnesiae DSM 18169 = JCM 13652	Bacteria	3.25
488	1121866	Enterorhabdus mucosicola DSM 19490	Bacteria	3.23
489	368735.1	Bell pepper mottle virus	Viruses	3.23
490	1903262.1	Bacteroides sp. Marseille-P3108 sp. Marseille-P3108	Bacteria	3.23
491	1686296.1	Gabonia massiliensis strain GM3	Bacteria	3.22
492	742737	Hungatella hathewayi WAL-18680	Bacteria	3.20
493	1261634.1	Roseburia sp. 499	Bacteria	3.19
494	1609975.1	Clostridium sp. FS41	Bacteria	3.19
495	44742.1	Desulfovibrio fairfieldensis strain CCUG	Bacteria	3.16
496	562.2194	Escherichia coli strain UPEC_014	Bacteria	3.12
497	1339307	Bacteroides fragilis str. 3719 A10	Bacteria	3.10
498	1121344	[Clostridium] viride DSM 6836	Bacteria	3.08
499	157777.1	Pea streak virus isolate VRS-541	Viruses	3.06
500	1522.3	[Clostridium] innocuum strain NLAE-zl-C381	Bacteria	3.05
501	33038.2	2789STDY5608852	Bacteria	3.02
502	537007	Blautia hansenii DSM 20583	Bacteria	3.00
503	665938.1	Bacteroides sp. 2_1_56FAA	Bacteria	2.97
504	480391.1	Pediococcus argentinicus strain DSM	Bacteria	2.96
505	1907658.1	Bacteroides sp. Marseille-P3208T strain Marseille-P3208	Bacteria	2.93
506	888056	Actinomyces sp. oral taxon 448 str. F0400	Bacteria	2.92
507	1736.1	Eubacterium limosum strain 32_A2	Bacteria	2.91
508	53443.1	Blautia hydrogenotrophica strain 2789STDY5608857	Bacteria	2.91
509	1232460.1	Clostridiales bacterium VE202-28	Bacteria	2.91
510	999410	[Clostridium] clostridioforme CM201	Bacteria	2.88
511	665937.1	Anaerostipes sp. 3_2_56FAA	Bacteria	2.86
512	39482.1	[Eubacterium] contortum strain 2789STDY5834876	Bacteria	2.85
513	1496.4	Clostridioides difficile isolate VL_0437	Bacteria	2.85
514	1384063	Ruminococcus gnavus AGR2154	Bacteria	2.83
515	1232447.1	Clostridiales bacterium VE202-09	Bacteria	2.82
516	339860	Methanosphaera stadtmanae DSM 3091	Archaea	2.80
517	880526	Rikenella microfusus DSM 15922	Bacteria	2.77
518	1496.59	Clostridioides difficile strain CD105KSE11	Bacteria	2.77
519	866499	Cloacibacillus evryensis DSM 19522	Bacteria	2.76
520	1232442.1	Clostridiales bacterium VE202-06	Bacteria	2.74

521	1870994.1	Urmitella timonensis sp. Marseille-P2918	Bacteria	2.73
522	445975	Collinsella stercoris DSM 13279	Bacteria	2.71
523	457422.1	Erysipelotrichaceae bacterium 2_2_44A	Bacteria	2.70
524	469597.1	Coprobacillus sp. 8_2_54BFAA	Bacteria	2.70
525	658083.1	Lachnospiraceae bacterium 6_1_63FAA	Bacteria	2.70
526	1321817	Actinomyces graevenitzii F0530	Bacteria	2.59
527	1120979	Alloscardovia omnicolens DSM 21503	Bacteria	2.56
528	1423794	Lactobacillus pontis DSM 8475	Bacteria	2.55
529	1816677.1	Butyricimonas sp. Marseille-P2440 sp. Marseille-P2440	Bacteria	2.54
530	658088.1	Lachnospiraceae bacterium 9_1_43BFAA	Bacteria	2.53
531	944168.1	Blastocystis sp. subtype 3	Eukaryota	2.51
532	665941.1	Coprobacillus sp. 3_3_56FAA	Bacteria	2.50
533	1122217	Megamonas rupellensis DSM 19944	Bacteria	2.48
534	84024.3	Clostridium disporicum strain 2789STDY5834856	Bacteria	2.48
535	817.22	Bacteroides fragilis strain DCMOUH0018B	Bacteria	2.44
536	1118060	Enorma massiliensis phI	Bacteria	2.40
537	47900.2	Garlic common latent virus isolate SW3.2	Viruses	2.39
538	187979.1	Mitsuokella jalaludinii strain 2789STDY5608828	Bacteria	2.38
539	1574264.1	Akkermansia sp. KLE1797	Bacteria	2.37
540	1602171.1	Prevotella sp. P5-119	Bacteria	2.35
541	518634	Bifidobacterium breve DSM 20213 = JCM 1192	Bacteria	2.35
542	59201.96	Salmonella enterica subsp. enterica strain SE696A	Bacteria	2.30
543	556268	Oxalobacter formigenes HOxBLS	Bacteria	2.29
544	1852383.1	Ruminococcaceae bacterium Marseille-P2935	Bacteria	2.28
545	59201.11	Salmonella enterica subsp. enterica strain ADRDL-LA-5-2013	Bacteria	2.28
546	649755	Faecalitalea cylindroides ATCC 27803	Bacteria	2.26
547	478820	Blastocystis sp. ATCC 50177/Nand II	Eukaryota	2.26
548	1235797.1	Oscillibacter sp. 1-3	Bacteria	2.26
549	908937	Prevotella dentalis DSM 3688	Bacteria	2.25
550	1736.2	Eubacterium limosum strain ATCC	Bacteria	2.21
551	1122971	Porphyromonas bennonis DSM 23058 = JCM 16335	Bacteria	2.21
552	742734	[Clostridium] citroniae WAL-19142	Bacteria	2.21
553	649757	Anaerostipes hadrus DSM 3319	Bacteria	2.18
554	39778.1	Veillonella dispar strain DNF00926	Bacteria	2.17
555	1496.33	Clostridioides difficile strain 7.10492	Bacteria	2.16
556	693979	Bacteroides helcogenes P 36-108	Bacteria	2.15
557	1739394.1	Porphyromonas sp. HMSC065F10	Bacteria	2.12
558	1796616.1	Blautia sp. YL58 sp. YL58	Bacteria	2.09
559	999403	[Clostridium] clostridioforme 90A1	Bacteria	2.07
560	742733	[Clostridium] citroniae WAL-17108	Bacteria	2.04
561	28125.4	Prevotella bivia strain GED7880	Bacteria	2.03
562	469587.1	Bacteroides sp. 2_1_16	Bacteria	2.03
563	1230734.1	Clostridiales bacterium S5-A14a	Bacteria	2.03
564	412133	Trichomonas vaginalis G3	Eukaryota	2.02
565	502558.1	Eggerthella sp. YY7918	Bacteria	2.02

566	1658109.1	Candidatus Stoquefichus sp. SB1	Bacteria	2.01
567	1235794	Enterorhabdus caecimuris B7	Bacteria	1.98
568	817.5	Bacteroides fragilis	Bacteria	1.98
569	1491.27	Clostridium botulinum strain SU0801	Bacteria	1.98
570	997897	[Clostridium] bolteae 90B8	Bacteria	1.97
571	665942.1	Desulfovibrio sp. 6_1_46AFAA	Bacteria	1.95
572	1585974.1	Beduini massiliensis strain GM1	Bacteria	1.95
573	442302.1	Porcine picobirnavirus strain 221/04-16/ITA/2004	Viruses	1.95
574	817.2	Bacteroides fragilis strain DCMOUH0067B	Bacteria	1.92
575	883077	Actinomyces turicensis ACS-279-V-Col4	Bacteria	1.91
576	47900.1	Garlic common latent virus	Viruses	1.90
577	747056.1	Blueberry shock virus isolate Berkely	Viruses	1.89
578	706434	Megasphaera micronuciformis F0359	Bacteria	1.89
579	665943.1	Eggerthella sp. 1_3_56FAA	Bacteria	1.87
580	817.8	Bacteroides fragilis strain JIM10	Bacteria	1.87
581	1235789	Parabacteroides goldsteinii dnLKV18	Bacteria	1.85
582	1395125	Prevotella salivae F0493	Bacteria	1.84
583	1564113.1	Sphingomonas sp. Ant H11	Bacteria	1.83
584	1123313	Faecalicoccus pleomorphus DSM 20574	Bacteria	1.82
585	252598.1	Saccharomyces sp. 'boulardii' strain unique28	Eukaryota	1.82
586	509923.1	Beet cryptic virus 1	Viruses	1.80
587	1603888.1	Megasphaera sp. MJR8396C	Bacteria	1.80
588	154288.4	Turicibacter sanguinis strain 2789STDY5834949	Bacteria	1.78
589	1632013.1	Drancourtella massiliensis strain GD1	Bacteria	1.78
590	1611875.1	RNA	Viruses	1.78
591	1903261.1	Desulfovibrio sp. Marseille-P3199 sp. Marseille-P3199	Bacteria	1.77
592	1602169.1	Prevotella sp. P4-76	Bacteria	1.75
593	1871016.1	Collinsella sp. Marseille-P3245 sp. Marseille-P3245	Bacteria	1.73
594	1078090.1	Coprococcus sp. HPP0074	Bacteria	1.72
595	706436	Capnocytophaga sp. oral taxon 329 str. F0087	Bacteria	1.72
596	1073388	Bacteroides fragilis HMW 616	Bacteria	1.71
597	1030842	Ruminococcus flavefaciens 17	Bacteria	1.69
598	470.218	Acinetobacter baumannii strain ABBL027	Bacteria	1.69
599	999412	Hungatella hathewayi 12489931	Bacteria	1.67
600	1720313.1	Bittarella massiliensis strain GD6	Bacteria	1.67
601	1871022.1	Libanicoccus massiliensis strain Marseille-P3237T sp. Marseille-P3237	Bacteria	1.65
602	747056.2	Blueberry shock virus	Viruses	1.65
603	1339275	Bacteroides fragilis str. Ds-233	Bacteria	1.64
604	742735	[Clostridium] clostridioforme 2_1_49FAA	Bacteria	1.62
605	857135	Streptococcus mutans U138	Bacteria	1.62
606	39950.1	Dialister pneumosintes strain F0677	Bacteria	1.62
607	1280695	[Clostridium] clostridioforme AGR2157	Bacteria	1.57
608	1437603	Bifidobacterium mongoliense DSM 21395	Bacteria	1.56
609	1069534	Lactobacillus ruminis ATCC 27782	Bacteria	1.56
610	742723.1	Lachnospiraceae bacterium 2_1_46FAA	Bacteria	1.56

611	1352.159	Enterococcus faecium isolate Hp_6-9	Bacteria	1.55
612	1232452.1	Clostridiales bacterium VE202-14	Bacteria	1.54
613	1323529.1	Dill cryptic virus 2 isolate IPP_hortorum	Viruses	1.53
614	1499684.1	Clostridium sp. CL-2	Bacteria	1.53
615	1907654.1	Collinsella sp. Marseille-P3296T strain Marseille-P3296	Bacteria	1.52
616	1834196.1	Lachnoclostridium sp. YL32 sp. YL32	Bacteria	1.52
617	90371.26	Salmonella enterica subsp. enterica serovar Typhimurium strain CFSAN033887	Bacteria	1.52
618	658665.1	Dorea sp. D27	Bacteria	1.51
619	552396.1	Erysipelotrichaceae bacterium 5_2_54FAA	Bacteria	1.51
620	755172.1	Peptoniphilus coxii strain DNF00729	Bacteria	1.50
621	1602170.1	Prevotella sp. P5-60	Bacteria	1.49
622	1531.2	[Clostridium] clostridioforme strain ATCC	Bacteria	1.49
623	679190	Prevotella buccalis ATCC 35310	Bacteria	1.49
624	33760.1	Prune dwarf virus	Viruses	1.49
625	1714570.1	Blueberry shoestring virus	Viruses	1.48
626	165432.1	Cucumber leaf spot virus	Viruses	1.47
627	1111120.1	Acidaminococcus sp. BV3L6	Bacteria	1.46
628	818.3	Bacteroides thetaiotaomicron strain 2789STDY5834846	Bacteria	1.46
629	1122989	Prevotella oris DSM 18711 = JCM 12252	Bacteria	1.45
630	1352.16	Enterococcus faecium isolate Hp_5-10	Bacteria	1.45
631	626522	Alloprevotella tannerae ATCC 51259	Bacteria	1.43
632	1841857.1	Culturomica massiliensis strain Marseille-P2698	Bacteria	1.40
633	1352.152	Enterococcus faecium isolate Hp_76-7	Bacteria	1.39
634	28901.45	Salmonella enterica strain NGUA07	Bacteria	1.39
635	742816	Megamonas funiformis YIT 11815	Bacteria	1.36
636	1797112.1	Olsenella sp. kh2p3 sp. kh2p3	Bacteria	1.34
637	1339274	Bacteroides fragilis str. A7 (UDC12-2)	Bacteria	1.32
638	1120921	Acidaminococcus intestini DSM 21505	Bacteria	1.32
639	1917878.1	Prevotella ihumii sp. Marseille-P3385	Bacteria	1.30
640	28901.37	Salmonella enterica strain NGUA04	Bacteria	1.30
641	1739517.1	Bacteroides sp. HMSC073E02	Bacteria	1.30
642	1432052.4	Eisenbergiella tayi strain NML140904	Bacteria	1.29
643	999406	[Clostridium] clostridioforme 90A6	Bacteria	1.29
644	641112	Ruminococcus flavefaciens FD-1	Bacteria	1.29
645	1105029.1	Actinomyces sp. ICM39	Bacteria	1.29
646	1280705	Prevotella bryantii C21a	Bacteria	1.27
647	84024.1	Clostridium disporicum strain 2789STDY5834855	Bacteria	1.27
648	1284775.1	Prevotella sp. S7-1-8	Bacteria	1.26
649	1581179.1	Clostridium sp. HMSC19A11	Bacteria	1.25
650	1339308	Bacteroides fragilis str. 3774 T13	Bacteria	1.25
651	1423719	Lactobacillus algidus DSM 15638	Bacteria	1.24
652	1078091.1	Coprococcus sp. HPP0048	Bacteria	1.24
653	1211843	Candidatus Soleaferrea massiliensis AP7	Bacteria	1.24
654	1739408.1	Streptococcus sp. HMSC072G04	Bacteria	1.23
655	469616	Fusobacterium mortiferum ATCC 9817	Bacteria	1.22

656	904144	Gardnerella vaginalis 101	Bacteria	1.21
657	596315	Peptostreptococcus stomatis DSM 17678	Bacteria	1.21
658	1739304.1	Anaerosphaera sp. HMSC064C01	Bacteria	1.20
659	1870997.1	Mogibacterium sp. Marseille-P3115 sp. Marseille-P3115	Bacteria	1.20
660	1232428.1	Megasphaera massiliensis strain NP3	Bacteria	1.19
661	658082.1	Lachnospiraceae bacterium 2_1_58FAA	Bacteria	1.19
662	1042156.1	Clostridium sp. SY8519	Bacteria	1.19
663	42817.1	Corynebacterium argentoratense strain CNM	Bacteria	1.19
664	1261636.1	Anaerostipes sp. 494a	Bacteria	1.17
665	1697788.1	Clostridia bacterium UC5.1-2H6	Bacteria	1.16
666	817.14	Bacteroides fragilis strain 2-078382-3	Bacteria	1.16
667	1120943	Actinomyces gerencseriae DSM 6844	Bacteria	1.16
668	1260.1	Finegoldia magna strain GED7760A	Bacteria	1.16
669	411466	Actinomyces odontolyticus ATCC 17982	Bacteria	1.16
670	1122975	Porphyromonas somerae DSM 23386	Bacteria	1.16
671	500635	Mitsuokella multacida DSM 20544	Bacteria	1.16
672	1401073	Prevotella melaninogenica DNF00666	Bacteria	1.11
673	12175.6	Apple chlorotic leaf spot virus	Viruses	1.11
674	857290	Scardovia wiggsiae F0424	Bacteria	1.08
675	585502	Prevotella bergensis DSM 17361	Bacteria	1.08
676	1851429.1	Christensenella sp. AF73-05CM02	Bacteria	1.07
677	1491.75	Clostridium botulinum strain KAPB-3	Bacteria	1.07
678	137838.1	Clostridium neonatale	Bacteria	1.06
679	11613	Tomato spotted wilt virus	Viruses	1.05
680	1408895.1	Dill cryptic virus 1 isolate IPP_hortorum	Viruses	1.05
681	411472	[Clostridium] symbiosum ATCC 14940	Bacteria	1.05
682	1410649	Blautia schinkii DSM 10518	Bacteria	1.05
683	568816	Acidaminococcus intestini RyC-MR95	Bacteria	1.03
684	1121334	[Clostridium] sporosphaeroides DSM 1294	Bacteria	1.03
685	1680.2	Bifidobacterium adolescentis strain Km	Bacteria	1.03
686	243563.3	Strawberry necrotic shock virus	Viruses	1.01
687	198589.1	Beet western yellows ST9 associated virus	Viruses	1.01
688	1122976	Porphyromonas uenonis DSM 23387 = JCM 13868	Bacteria	1.01
689	862962	Bacteroides fragilis 638R	Bacteria	1.01
690	679189	Prevotella timonensis CRIS 5C-B1	Bacteria	1.00
691	297352	Lactococcus piscium MKFS47	Bacteria	0.99
692	1834207.1	Erysipelotrichaceae bacterium I46	Bacteria	0.99
693	666.83	Vibrio cholerae strain MZO-2	Bacteria	0.98
694	791161	Enterococcus faecium PC4.1	Bacteria	0.98
695	562982	Gemella morbillorum M424	Bacteria	0.97
696	1280685.1	Butyrivibrio sp. NC3005	Bacteria	0.97
697	562.188	Escherichia coli strain USVAST406	Bacteria	0.96
698	457398.1	Desulfovibrio sp. 3_1_syn3	Bacteria	0.95
699	1122982	Prevotella denticola DSM 20614 = JCM 13449	Bacteria	0.95
700	295405	Bacteroides fragilis YCH46	Bacteria	0.94
701	246199	Ruminococcus albus 8	Bacteria	0.94

702	28128.1	Prevotella corporis strain MJR7716	Bacteria	0.93
703	1491.58	Clostridium botulinum strain DB-2	Bacteria	0.92
704	556261.1	Clostridium sp. D5	Bacteria	0.92
705	1414720.1	Clostridium saudiense strain JCC	Bacteria	0.91
706	1401062	Prevotella timonensis S9-PR14	Bacteria	0.91
707	1491.57	Clostridium botulinum strain 713_CBOT	Bacteria	0.89
708	1329795.1	Clostridiaceae bacterium MS3	Bacteria	0.89
709	817.26	Bacteroides fragilis strain S14	Bacteria	0.88
710	547043	Bifidobacterium pseudocatenulatum DSM 20438 = JCM 1200 = LMG 10505	Bacteria	0.88
711	270498.2	Catabacter hongkongensis strain HKU16	Bacteria	0.88
712	311413.2	Lettuce big-vein associated virus isolate Ls302	Viruses	0.86
713	1528099.1	Lawsonella clevelandensis	Bacteria	0.84
714	1111134.1	Peptoniphilus sp. BV3C26	Bacteria	0.83
715	525282	Finegoldia magna ATCC 53516	Bacteria	0.83
716	1235792.1	Lachnospiraceae bacterium M18-1	Bacteria	0.83
717	90371.43	Salmonella enterica subsp. enterica serovar Typhimurium strain CFSAN033867	Bacteria	0.82
718	559292	Saccharomyces cerevisiae S288C	Eukaryota	0.80
719	752555	Prevotella bryantii B14	Bacteria	0.79
720	1352.155	Enterococcus faecium isolate Hp_6-10	Bacteria	0.79
721	1308.7	Streptococcus thermophilus	Bacteria	0.79
722	1778580.3	Nectarine virus M isolate NeVM/12P42	Viruses	0.78
723	999425.1	Streptococcus sp. F0442	Bacteria	0.78
724	562.2364	Escherichia coli strain ICBECAM7	Bacteria	0.77
725	729.12	Haemophilus parainfluenzae strain 146_HPAR	Bacteria	0.77
726	1504822.1	bacterium OL-1	Bacteria	0.77
727	1151426	Clostridioides difficile P51	Bacteria	0.77
728	762967	Sutterella parvirubra YIT 11816	Bacteria	0.76
729	199.1	Campylobacter concisus strain RMIT-JF1	Bacteria	0.76
730	1232448.1	Clostridiales bacterium VE202-07	Bacteria	0.75
731	378833.1	Sowbane mosaic virus	Viruses	0.75
732	877411.1	Ruminococcus sp. NK3A76	Bacteria	0.74
733	370354	Entamoeba dispar SAW760	Eukaryota	0.74
734	596327	Porphyromonas uenonis 60-3	Bacteria	0.73
735	470.677	Acinetobacter baumannii strain XH753	Bacteria	0.73
736	70177.2	Grapevine leafroll-associated virus 4	Viruses	0.72
737	53442.1	Eubacterium callanderi strain FD	Bacteria	0.72
738	1378168.1	Firmicutes bacterium ASF500	Bacteria	0.71
739	861450	Anaeroglobus geminatus F0357	Bacteria	0.71
740	1496.426	Clostridioides difficile strain VRECD0128	Bacteria	0.70
741	42680.1	Spinach latent virus	Viruses	0.70
742	1339346	Bacteroides ovatus str. 3725 D1 iv	Bacteria	0.70
743	592010	Abiotrophia defectiva ATCC 49176	Bacteria	0.69
744	562.765	Escherichia coli isolate 14	Bacteria	0.69
745	1236498	Bacteroides paurosaccharolyticus JCM 15092	Bacteria	0.69
746	1583.2	Weissella confusa strain MBF8-1	Bacteria	0.68
747	1235811	Prevotella disiens JCM 6334 = ATCC 29426	Bacteria	0.68

748	1110546.1	Veillonella tobetsuensis strain ATCC	Bacteria	0.67
749	1235813	Bacteroides pyogenes JCM 10003	Bacteria	0.67
750	1437612	Bifidobacterium stercoris JCM 15918	Bacteria	0.67
751	1410665	Mitsuokella jalaludinii DSM 13811	Bacteria	0.66
752	1673717.1	Anaeromassilibacillus senegalensis strain mt9	Bacteria	0.66
753	28126.1	Prevotella buccae strain 1205_PDEN	Bacteria	0.66
754	1352.149	Enterococcus faecium isolate Hp_22-12	Bacteria	0.65
755	1203555.1	Acidaminococcus sp. HPA0509	Bacteria	0.65
756	1512.1	[Clostridium] symbiosum strain 2789STDY5834864	Bacteria	0.65
757	1264.1	Ruminococcus albus strain AR67	Bacteria	0.65
758	1776081.1	Megasphaera sp. DISK 18	Bacteria	0.65
759	666.79	Vibrio cholerae strain 3272-78	Bacteria	0.64
760	1122983	Prevotella falsenii DSM 22864 = JCM 15124	Bacteria	0.64
761	60920.1	Sanguibacter keddieii strain 250_SKED	Bacteria	0.64
762	1588755.1	Parvimonas sp. KA00067	Bacteria	0.63
763	592031	Eubacterium saphenum ATCC 49989	Bacteria	0.62
764	698950	Gardnerella vaginalis 284V	Bacteria	0.62
765	478749	Marvinbryantia formatexigens DSM 14469	Bacteria	0.61
766	28901.31	Salmonella enterica strain NGUA25	Bacteria	0.61
767	411481	Bifidobacterium adolescentis L2-32	Bacteria	0.61
768	187101.1	Sneathia amnii sp. Sn35	Bacteria	0.60
769	1173061.1	Geotrichum candidum strain CLIB	Eukaryota	0.60
770	1577792.1	Terrisporobacter othiniensis strain 08-306576	Bacteria	0.60
771	997895	[Clostridium] bolteae 90B3	Bacteria	0.60
772	575611	Prevotella buccae D17	Bacteria	0.60
773	1352.154	Enterococcus faecium isolate Hp_21-11	Bacteria	0.59
774	1339277	Bacteroides fragilis str. DS-71	Bacteria	0.59
775	1073375	Ruminococcus gnavus CC55_001C	Bacteria	0.59
776	817.4	Bacteroides fragilis strain DCMOUH0017B	Bacteria	0.59
777	1339278	Bacteroides fragilis str. DS-166	Bacteria	0.58
778	762963	Actinomyces sp. oral taxon 170 str. F0386	Bacteria	0.58
779	1261637.1	Anaerostipes sp. 992a	Bacteria	0.57
780	1035195	Corynebacterium durum F0235	Bacteria	0.57
781	679191	Prevotella amnii CRIS 21A-A	Bacteria	0.57
782	500633	[Clostridium] hiranonis DSM 13275	Bacteria	0.57
783	28026.6	B29	Bacteria	0.56
784	1679.14	Bifidobacterium longum subsp. longum strain CCUG30698	Bacteria	0.56
785	12042.2	Beet western yellows virus	Viruses	0.56
786	1292034	Caulobacter crescentus OR37	Bacteria	0.56
787	31722.2	Blueberry scorch virus isolate BC-2	Viruses	0.55
788	1121097	Bacteroides graminisolvens DSM 19988 = JCM 15093	Bacteria	0.55
789	1321775	Actinomyces sp. oral taxon 172 str. F0311	Bacteria	0.55
790	1007096	Oscillibacter ruminantium GH1	Bacteria	0.54
791	1871336.1	Criibacterium bergeronii strain CCRI-22567	Bacteria	0.54
792	1105030.1	Actinomyces sp. ICM58	Bacteria	0.54

793	1313215	Aichi virus 1	Viruses	0.54
794	1122992	Prevotella timonensis 4401737 = DSM 22865 = JCM 15640	Bacteria	0.53
795	525362	Lactobacillus ruminis ATCC 25644	Bacteria	0.53
796	12451.1	Raspberry bushy dwarf virus	Viruses	0.53
797	699246	Mageeibacillus indolicus UPII9-5	Bacteria	0.53
798	1720195.1	Gabonibacter massiliensis strain GM7	Bacteria	0.53
799	1393034.1	Atopobium deltae strain DNF00019	Bacteria	0.51
800	77095.1	Prevotella bryantii strain KHPX14	Bacteria	0.51
801	1697790.1	Clostridia bacterium UC5.1-1C12	Bacteria	0.51
802	322505.1	Sharpea azabuensis strain DSM	Bacteria	0.50
803	84024.2	Clostridium disporicum strain 2789STDY5608827	Bacteria	0.50
804	1352.15	Enterococcus faecium isolate Hp_23-9	Bacteria	0.50
805	1265.2	Ruminococcus flavefaciens strain Y1	Bacteria	0.50
806	1736.3	Eubacterium limosum strain SA11	Bacteria	0.50
807	77095.2	Prevotella bryantii strain FB3001	Bacteria	0.50
808	1051631.1	Streptococcus phage YMC-2011	Viruses	0.50
809	1532180.1	Penicillium roqueforti ssRNA mycovirus 1 strain PRG42-7	Viruses	0.50
810	1852386.1	Olsenella sp. Marseille-P2912 sp. Marseille-P2912	Bacteria	0.49
811	1161902	Eubacterium nodatum ATCC 33099	Bacteria	0.49
812	1111135.1	Coriobacteriaceae bacterium BV3Ac1	Bacteria	0.49
813	556270.1	Coprobacillus sp. D7	Bacteria	0.48
814	1531429.1	Coriobacteriaceae bacterium 68-1-3	Bacteria	0.48
815	1339292	Bacteroides fragilis str. 1007-1-F #7	Bacteria	0.48
816	1232446.1	Clostridiales bacterium VE202-18	Bacteria	0.48
817	1739529.1	Porphyromonas sp. HMSC077F02	Bacteria	0.48
818	11987.2	Melon necrotic spot virus	Viruses	0.47
819	1401074	Prevotella buccalis DNF00853	Bacteria	0.47
820	1805478.1	Olsenella sp. Marseille-P2300	Bacteria	0.47
821	817.15	Bacteroides fragilis strain 86-5443-2-2	Bacteria	0.47
822	642478.1	Lettuce chlorosis virus	Viruses	0.47
823	1697792.1	Clostridia bacterium UC5.1-2F7	Bacteria	0.47
824	1680.1	Bifidobacterium adolescentis strain 2789STDY5608862	Bacteria	0.47
825	1852374.1	Ezakiella massiliensis strain Marseille-P2951T sp. Marseille-P2951	Bacteria	0.47
826	66200.1	Carrot red leaf virus	Viruses	0.47
827	585501	Oribacterium sinus F0268	Bacteria	0.46
828	888050	Actinomyces cardiffensis F0333	Bacteria	0.46
829	608534	Oribacterium sp. oral taxon 078 str. F0262	Bacteria	0.46
830	877414.1	Clostridiales bacterium NK3B98	Bacteria	0.46
831	1852375.1	Acidaminococcus massiliensis strain Marseille-P2828	Bacteria	0.45
832	28901.13	Salmonella enterica strain NGUA10	Bacteria	0.45
833	28026.3	Bifidobacterium pseudocatenulatum strain CA-K29a	Bacteria	0.45
834	580254.1	Raphanus sativus cryptic virus 3	Viruses	0.45

835	483214	Methanobrevibacter smithii DSM 2375	Archaea	0.44
836	742741	[Clostridium] symbiosum WAL-14673	Bacteria	0.44
837	729.1	Haemophilus parainfluenzae strain 215035-2-ISO5	Bacteria	0.44
838	1351.95	Enterococcus faecalis isolate Hp_74-d1	Bacteria	0.44
839	1680.5	Bifidobacterium adolescentis strain 22L	Bacteria	0.44
840	1522.2	2789STDY5834853	Bacteria	0.44
841	1673723.1	Murdochiella massiliensis strain SIT12	Bacteria	0.43
842	216816.7	Bifidobacterium longum isolate Bifido_09	Bacteria	0.43
843	1392494.1	Lachnospiraceae bacterium AC2012	Bacteria	0.43
844	1392836.1	Lachnospiraceae bacterium TWA4	Bacteria	0.43
845	633697	[Eubacterium] cellulosolvens 6	Bacteria	0.42
846	1226323.1	Oscillibacter sp. KLE 1745	Bacteria	0.42
847	290055.1	[Eubacterium] fissicatena strain KCTC	Bacteria	0.42
848	431317.1	Triticum mosaic virus	Viruses	0.42
849	1211844	Candidatus Stoquefichus massiliensis AP9	Bacteria	0.42
850	12433.1	Garlic virus A	Viruses	0.42
851	1321819	Bacteroides pyogenes F0041	Bacteria	0.41
852	1232445.1	Clostridiales bacterium VE202-16	Bacteria	0.41
853	1365967	Bifidobacterium breve MCC 1454	Bacteria	0.40
854	997894	[Clostridium] bolteae 90A9	Bacteria	0.40
855	575593	Lachnospiraceae oral taxon 107 str. F0167	Bacteria	0.40
856	1048332	Streptococcus salivarius CCHSS3	Bacteria	0.40
857	1352.156	Enterococcus faecium isolate Hp_24-3	Bacteria	0.39
858	1336250	Hallella seregens ATCC 51272	Bacteria	0.39
859	1033744	Peptoniphilus senegalensis JC140	Bacteria	0.39
860	1035196	Peptostreptococcus anaerobius VPI 4330 = DSM 2949	Bacteria	0.39
861	31722.3	Blueberry scorch virus	Viruses	0.39
862	457404	Fusobacterium ulcerans 12-1B	Bacteria	0.39
863	827.1	Campylobacter ureolyticus strain CIT007	Bacteria	0.39
864	1776177.1	Cucumis melo endornavirus isolate CL-01	Viruses	0.39
865	1215078	Clostridioides difficile E7	Bacteria	0.38
866	457396.1	Clostridium sp. 7_2_43FAA	Bacteria	0.38
867	1489836.1	Atlantic salmon calicivirus isolate Nordland/2011	Viruses	0.38
868	97478.5	Lactobacillus mucosae strain DPC	Bacteria	0.37
869	1581061.1	Abiotrophia sp. HMSC24B09	Bacteria	0.37
870	12187.2	Strawberry mild yellow edge virus	Viruses	0.37
871	103722.1	Grapevine fleck virus	Viruses	0.37
872	1341157	Ruminococcus flavefaciens 007c	Bacteria	0.36
873	5007.1	Brettanomyces bruxellensis	Eukaryota	0.36
874	1676617.1	Ralstonia sp. MD27	Bacteria	0.35
875	1499683.1	Clostridium sp. CL-6	Bacteria	0.35
876	31722.1	Blueberry scorch virus isolate BC-1	Viruses	0.35
877	469378	Cryptobacterium curtum DSM 15641	Bacteria	0.35
878	883109	Eubacterium infirmum F0142	Bacteria	0.35
879	1321814	Eubacterium brachy ATCC 33089	Bacteria	0.34
880	154288.2	Turicibacter sanguinis strain 2789STDY5608821	Bacteria	0.34
881	1522.1	[Clostridium] innocuum strain AN88	Bacteria	0.34

882	888833	Streptococcus australis ATCC 700641	Bacteria	0.33
883	397544.4	Squash vein yellowing virus isolate IL	Viruses	0.33
884	352165	Pyramidobacter piscolens W5455	Bacteria	0.33
885	908340.1	Clostridium sp. HGF2	Bacteria	0.33
886	145856.1	Human picobirnavirus	Viruses	0.33
887	1561.1	Clostridium baratii strain XCM	Bacteria	0.32
888	887898	Lautropia mirabilis ATCC 51599	Bacteria	0.32
889	1282887	Lachnospira multipara ATCC 19207	Bacteria	0.32
890	642492	Clostridium lentocellum DSM 5427	Bacteria	0.32
891	688246	Prevotella multisaccharivorax DSM 17128	Bacteria	0.32
892	1680.7	Bifidobacterium adolescentis strain 150	Bacteria	0.32
893	1391469	Enterococcus faecalis BM4654	Bacteria	0.31
894	1284680.1	Actinomyces sp. S6-Spd3	Bacteria	0.31
895	1203606	Butyricicoccus pullicaecorum 1.2	Bacteria	0.31
896	1125712	Olsenella profusa F0195	Bacteria	0.31
897	1776390.1	Peptoniphilus sp. KHD5 sp. KHD5	Bacteria	0.30
898	1114972	Lactobacillus rossiae DSM 15814	Bacteria	0.30
899	742767	Dysgonomonas mossii DSM 22836	Bacteria	0.30
900	1219626.1	Peptostreptococcus sp. MV1	Bacteria	0.30
901	1304.27	Streptococcus salivarius strain KB005	Bacteria	0.30
902	1339270	Bacteroides fragilis str. I1345	Bacteria	0.30
903	1280702	Bifidobacterium longum AGR2137	Bacteria	0.30
904	1121485	Dysgonomonas capnocytophagoides DSM 22835	Bacteria	0.30
905	329.3	Ralstonia pickettii strain CW2	Bacteria	0.29
906	1304.11	Streptococcus salivarius strain 37-08	Bacteria	0.29
907	1458465	Megasphaera elsdenii 14-14	Bacteria	0.29
908	32625.1	Mushroom bacilliform virus	Viruses	0.29
909	796942.1	Stomatobaculum longum	Bacteria	0.29
910	216816.1	Bifidobacterium longum isolate Bifido_03	Bacteria	0.29
911	12282.1	Tobacco ringspot virus	Viruses	0.29
912	768727	Veillonella parvula ACS-068-V-Sch12	Bacteria	0.29
913	762983	Succinatimonas hippei YIT 12066	Bacteria	0.29
914	28026.7	Bifidobacterium pseudocatenulatum strain 2789STDY5834840	Bacteria	0.29
915	1125718	Actinomyces massiliensis F0489	Bacteria	0.29
916	1871012.1	Mobilibacterium timonense strain Marseille-P3194	Bacteria	0.29
917	1121864	Enterococcus cecorum DSM 20682 = ATCC 43198	Bacteria	0.28
918	29466.2	Veillonella parvula strain UTDB1-3	Bacteria	0.28
919	193118.1	Lucerne transient streak virus satellite RNA	Viruses	0.28
920	1689303.1	Lagierella massiliensis strain SIT14	Bacteria	0.28
921	1410644	Bifidobacterium adolescentis DSM 20087	Bacteria	0.28
922	39046.1	Cassava common mosaic virus	Viruses	0.28
923	595.15	Salmonella enterica subsp. enterica serovar Infantis strain 120100	Bacteria	0.28
924	649761	Prevotella veroralis F0319	Bacteria	0.28
925	1408439	Fusobacterium perfoetens ATCC 29250	Bacteria	0.28

926	1680.3	Bifidobacterium adolescentis strain 2789STDY5834850	Bacteria	0.28
927	1739305.1	Peptoniphilus sp. HMSC062D09	Bacteria	0.28
928	1095750	Lachnoanaerobaculum saburreum F0468	Bacteria	0.28
929	354328.3	Bell pepper endornavirus	Viruses	0.28
930	1229758	Leuconostoc carnosum JB16	Bacteria	0.28
931	12282.2	Tobacco ringspot virus isolate SK	Viruses	0.28
932	563031.1	Prevotella sp. C561	Bacteria	0.28
933	1680.6	Bifidobacterium adolescentis strain BBMN23	Bacteria	0.28
934	1122150	Lactobacillus nagelii DSM 13675	Bacteria	0.28
935	1588754.1	Veillonellaceae bacterium DNF00626	Bacteria	0.28
936	859.1	Fusobacterium necrophorum strain ATCC	Bacteria	0.27
937	566552	Bifidobacterium catenulatum DSM 16992 = JCM 1194 = LMG 11043	Bacteria	0.27
938	1871017.1	Peptoniphilus urinimassiliensis strain Marseille-P3195	Bacteria	0.27
939	1679.8	Bifidobacterium longum subsp. longum strain LO-K29a	Bacteria	0.27
940	198599.1	Saccharomyces 23S RNA narnavirus	Viruses	0.27
941	1339315	Bacteroides fragilis str. 3988T(B)14	Bacteria	0.27
942	54062.1	Pediococcus parvulus strain 2.6	Bacteria	0.27
943	1338.1	Streptococcus intermedius strain 631_SCON	Bacteria	0.27
944	905.1	Acidaminococcus fermentans strain pGA-4	Bacteria	0.27
945	1161745	Bifidobacterium longum subsp. longum 2-2B	Bacteria	0.26
946	29833.1	Hanseniaspora uvarum	Eukaryota	0.26
947	1120944	Actinomyces israelii DSM 43320	Bacteria	0.26
948	1122216	Megamonas hypermegale DSM 1672	Bacteria	0.26
949	1852387.1	Streptococcus timonensis strain Marseille-P2915	Bacteria	0.26
950	706433	Solobacterium moorei F0204	Bacteria	0.26
951	244362.1	Ruminococcus sp. YE71 sp. YE71	Bacteria	0.25
952	1778580.1	Nectarine virus M isolate NeVM/SF04522E	Viruses	0.25
953	883156	Veillonella seminalis ACS-216-V-Col6b	Bacteria	0.25
954	1339272	Bacteroides fragilis str. J38-1	Bacteria	0.25
955	1301220.1	Citrus vein enation virus isolate VE-1	Viruses	0.25
956	997896	[Clostridium] bolteae 90B7	Bacteria	0.25
957	40543.1	Sneathia sanguinegens strain CCUG41628	Bacteria	0.25
958	450749.1	Veillonella sp. 6_1_27	Bacteria	0.25
959	1449897.1	Uncultured phage WW-nAnB strain 3 strain 3	Viruses	0.25
960	12145.1	Tomato bushy stunt virus	Viruses	0.24
961	1805477.1	Clostridium sp. Marseille-P299	Bacteria	0.24
962	521095	Atopobium parvulum DSM 20469	Bacteria	0.24
963	729.13	Haemophilus parainfluenzae strain 1209_HPAR	Bacteria	0.24
964	1463935.1	Streptomyces sp. NRRL WC-3744	Bacteria	0.24
965	28026.1	D29	Bacteria	0.24
966	1415630.1	Pseudomonas sp. TKP sp. TKP	Bacteria	0.23
967	39029.1	Megasphaera cerevisiae strain NSB1	Bacteria	0.23
968	216816.9	Bifidobacterium longum isolate Bifido_12	Bacteria	0.23
969	112227.1	Cactus virus X	Viruses	0.23

970	29466.1	Veillonella parvula strain DNF00876	Bacteria	0.23
971	1123311	Streptococcus orisratti DSM 15617	Bacteria	0.23
972	12175.8	Apple chlorotic leaf spot virus isolate QD-13	Viruses	0.23
973	1348633	Lactococcus raffinolactis NBRC 100932	Bacteria	0.23
974	1040964	Lactobacillus ruminis SPM0211	Bacteria	0.23
975	1496.114	Clostridioides difficile isolate VL_0181	Bacteria	0.23
976	1423814	Lactobacillus vaginalis DSM 5837 = ATCC 49540	Bacteria	0.22
977	714313	Lactobacillus sanfranciscensis TMW 1.1304	Bacteria	0.22
978	29397.1	Lactobacillus delbrueckii subsp. lactis	Bacteria	0.22
979	887325	Lachnoanaerobaculum saburreum DSM 3986	Bacteria	0.22
980	1074044.1	uncultured phage WW-nAnB	Viruses	0.22
981	469617	Fusobacterium ulcerans ATCC 49185	Bacteria	0.22
982	1511761.1	Leuconostoc mesenteroides subsp. suionicum strain DSM	Bacteria	0.22
983	1125779	Corynebacterium pyruviciproducens ATCC BAA-1742	Bacteria	0.22
984	1778.5	Mycobacterium gordonae strain HMC_M15	Bacteria	0.22
985	1739251.1	Fusobacterium sp. HMSC073F01	Bacteria	0.22
986	479436	Veillonella parvula DSM 2008	Bacteria	0.21
987	397288.1	Lachnospiraceae bacterium 3-1	Bacteria	0.21
988	563191.1	Acidaminococcus sp. D21	Bacteria	0.21
989	646413.1	Streptococcus phage 5093	Viruses	0.21
990	645512	Jonquetella anthropi E3_33 E1	Bacteria	0.21
991	565040	Bifidobacterium longum subsp. infantis 157F	Bacteria	0.21
992	12263.1	Squash mosaic virus isolate CH	Viruses	0.21
993	35350.4	Apple stem pitting virus	Viruses	0.20
994	12317.6	Tobacco streak virus isolate 1973	Viruses	0.20
995	243563.2	Strawberry necrotic shock virus isolate Florida-4	Viruses	0.20
996	1776381.1	Olsenella sp. KHD7 sp. KHD7	Bacteria	0.20
997	760570	Streptococcus parasanguinis ATCC 15912	Bacteria	0.20
998	1653434.1	Sellimonas intestinalis strain BR72	Bacteria	0.20
999	44008.8	Enterococcus cecorum strain G-29	Bacteria	0.20
1000	322159	Streptococcus thermophilus LMD-9	Bacteria	0.20
1001	537288.1	Megasphaera sp. DJF_B143	Bacteria	0.20
1002	1583.1	Weissella confusa strain DSM	Bacteria	0.20
1003	1852372.1	Varibaculum sp. Marseille-P2802 sp. Marseille-P2802	Bacteria	0.20
1004	907931	Leuconostoc fallax KCTC 3537	Bacteria	0.20
1005	1261.3	Peptostreptococcus anaerobius strain MJR8628A	Bacteria	0.20
1006	1681.1	Bifidobacterium bifidum strain 791	Bacteria	0.19
1007	938288.1	Fenollaria massiliensis	Bacteria	0.19
1008	270498.1	Catabacter hongkongensis strain ABBA15k	Bacteria	0.19
1009	634994	Leptotrichia hofstadii F0254	Bacteria	0.19
1010	1501391.1	Alistipes inops strain 627	Bacteria	0.19
1011	1216932.1	Clostridium bornimense strain M2/40T	Bacteria	0.19
1012	546262	Neisseria cinerea ATCC 14685	Bacteria	0.19
1013	12242.1	Tobacco mosaic virus	Viruses	0.19
1014	12263.2	Squash mosaic virus	Viruses	0.19

1015	1236497	Prevotella oulorum JCM 14966	Bacteria	0.19
1016	596329	Peptostreptococcus anaerobius 653-L	Bacteria	0.18
1017	1720317.1	Porphyromonadaceae bacterium FC4	Bacteria	0.18
1018	1236689	Candidatus Methanomethylophilus alvus Mx1201	Archaea	0.18
1019	1871014.1	Arcanobacterium urinimassiliense sp. Marseille-P3248	Bacteria	0.18
1020	1391466	Enterococcus faecium NEF1	Bacteria	0.18
1021	1658108.1	Niameybacter massiliensis	Bacteria	0.18
1022	209529.6	Aphid lethal paralysis virus isolate AP	Viruses	0.18
1023	712122.1	Actinomyces sp. oral taxon 414 strain F0588	Bacteria	0.18
1024	1432052.5	Eisenbergiella tayi strain NML110678	Bacteria	0.18
1025	143387.2	Fusobacterium necrophorum subsp. funduliforme strain LS_1272	Bacteria	0.18
1026	1449336	Carnobacterium divergens DSM 20623	Bacteria	0.18
1027	1871002.1	Acidaminococcus timonensis strain Marseille-P2764	Bacteria	0.18
1028	35350.7	Apple stem pitting virus isolate apple	Viruses	0.17
1029	1720204.1	Collinsella ihuae sp. GD7	Bacteria	0.17
1030	1347366.1	Clostridium sp. ND2	Bacteria	0.17
1031	1838287.1	Gammaproteobacteria bacterium 2W06	Bacteria	0.17
1032	12458	Garlic latent virus	Viruses	0.17
1033	78448.1	Bifidobacterium pullorum strain LMG	Bacteria	0.17
1034	1871033.1	Olsenella sp. Marseille-P3197 sp. Marseille-P3197	Bacteria	0.17
1035	1280689	Clostridium paraputrificum AGR2156	Bacteria	0.17
1036	367928	Bifidobacterium adolescentis ATCC 15703	Bacteria	0.17
1037	1073386	Bacteroides fragilis HMW 610	Bacteria	0.17
1038	1497955.1	Clostridiales bacterium KA00274	Bacteria	0.17
1039	1136138	Pseudomonas fragi B25	Bacteria	0.17
1040	1050201	Allobaculum stercoricanis DSM 13633	Bacteria	0.17
1041	525919	Anaerococcus prevotii DSM 20548	Bacteria	0.17
1042	1105031.1	Clostridium sp. MSTE9	Bacteria	0.17
1043	1680.4	Bifidobacterium adolescentis strain 2789STDY5608824	Bacteria	0.17
1044	817.9	Bacteroides fragilis strain 20793-3	Bacteria	0.17
1045	1852362.1	Bacteroides ihuae strain Marseille-P2824	Bacteria	0.17
1046	1778.3	Mycobacterium gordonae strain 1245752.6	Bacteria	0.17
1047	1304.5	Streptococcus salivarius strain 726_SSAL	Bacteria	0.17
1048	1739406.1	Actinomyces sp. HMSC035G02	Bacteria	0.17
1049	1401075	Prevotella disiens DNF00882	Bacteria	0.17
1050	1280674.1	Prevotella sp. AGR2160	Bacteria	0.17
1051	1321782	Oribacterium sp. oral taxon 078 str. F0263	Bacteria	0.17
1052	1167629	27673	Bacteria	0.17
1053	1497953.1	Bacteroidales bacterium KA00251	Bacteria	0.17
1054	1805470.1	Clostridium sp. Marseille-P2414 sp. Marseille-P2414	Bacteria	0.17
1055	28026.5	Bifidobacterium pseudocatenulatum strain CA-K29b	Bacteria	0.17

1056	103724.1	Grapevine asteroid mosaic-associated virus isolate GV30	Viruses	0.17
1057	936588.1	Veillonella sp. ACP1	Bacteria	0.17
1058	927691	Leuconostoc gelidum subsp. gelidum KCTC 3527	Bacteria	0.17
1059	287.9	Pseudomonas aeruginosa strain Pae_CF67.12q	Bacteria	0.17
1060	1715051.1	Streptococcus sp. HMSC068F04	Bacteria	0.17
1061	205913	Bifidobacterium longum DJO10A	Bacteria	0.17
1062	1352.145	Enterococcus faecium isolate Hp_7-8	Bacteria	0.16
1063	1561.3	Clostridium baratii strain 2789STDY5834956	Bacteria	0.16
1064	1581080.1	Streptococcus sp. HMSC10E12	Bacteria	0.16
1065	1111454.1	Megasphaera sp. BV3C16-1	Bacteria	0.16
1066	742766	Dysgonomonas gadei ATCC BAA-286	Bacteria	0.16
1067	1339288	Bacteroides fragilis str. 3988 T1	Bacteria	0.16
1068	1032506.1	Prevotella sp. MSX73	Bacteria	0.16
1069	1655645.1	Parabacteroides phage YZ-2015b	Viruses	0.16
1070	457416.1	Veillonella sp. 3_1_44	Bacteria	0.16
1071	1118062	Peptoniphilus obesi ph1	Bacteria	0.16
1072	1623.1	Lactobacillus ruminis strain WC1T17	Bacteria	0.16
1073	1588753.1	Coriobacteriales bacterium DNF00809	Bacteria	0.16
1074	546269	Filifactor alocis ATCC 35896	Bacteria	0.16
1075	139208.1	Isoptericola variabilis strain 871_IVAR	Bacteria	0.16
1076	1203593.1	Veillonella sp. HPA0037	Bacteria	0.16
1077	879243	Porphyromonas asaccharolytica DSM 20707	Bacteria	0.15
1078	817.23	Bacteroides fragilis strain BOB25	Bacteria	0.15
1079	867080.1	Paenibacillus sp. IHB B 3415	Bacteria	0.15
1080	1720315.1	Eggerthellaceae bacterium AT8	Bacteria	0.15
1081	1410673	Selenomonas bovis 8-14-1	Bacteria	0.15
1082	525146	Desulfovibrio desulfuricans subsp. desulfuricans str. ATCC 27774	Bacteria	0.15
1083	1352.101	Enterococcus faecium strain XH877	Bacteria	0.15
1084	1423747	Lactobacillus fuchuensis DSM 14340 = JCM 11249	Bacteria	0.15
1085	1302.13	Streptococcus gordonii strain M5	Bacteria	0.15
1086	873513	Prevotella buccae ATCC 33574	Bacteria	0.15
1087	184922	Giardia lamblia ATCC 50803	Eukaryota	0.15
1088	1339306	Bacteroides fragilis str. 3719 T6	Bacteria	0.15
1089	1871023.1	Rikenella sp. Marseille-P3215 sp. Marseille-P3215	Bacteria	0.15
1090	548908.1	Fig fleck-associated virus	Viruses	0.15
1091	28347.12	Apple stem grooving virus clone ASGVp12	Viruses	0.15
1092	883069	Actinomyces europaeus ACS-120-V-Col10b	Bacteria	0.15
1093	1293039	Methanobrevibacter arboriphilus JCM 9315	Archaea	0.15
1094	206672	Bifidobacterium longum NCC2705	Bacteria	0.15
1095	1206566.2	Blueberry virus A isolate Elliot	Viruses	0.14
1096	1161409.1	Bifidobacterium sp. MSTE12	Bacteria	0.14
1097	1410621.1	Lachnospiraceae bacterium AD3010	Bacteria	0.14
1098	742740	[Clostridium] symbiosum WAL-14163	Bacteria	0.14
1099	12172.3	Shallot latent virus isolate WA-1	Viruses	0.14
1100	112227.2	Cactus virus X strain SCM51431	Viruses	0.14

1101	1308.1	Streptococcus thermophilus strain KLDS	Bacteria	0.14
1102	1351.93	Enterococcus faecalis isolate Hp_74-d5	Bacteria	0.14
1103	1629.2	Weissella viridescens strain NCDO	Bacteria	0.14
1104	1623.2	Lactobacillus ruminis strain DPC	Bacteria	0.14
1105	469618	Fusobacterium varium ATCC 27725	Bacteria	0.14
1106	879305	Anaerococcus prevotii ACS-065-V-Col13	Bacteria	0.14
1107	1052902	Tobacco mosaic virus strain Ohio V	Viruses	0.14
1108	1739525.1	Peptoniphilus sp. HMSC075B08	Bacteria	0.14
1109	1401072	Prevotella bivia DNF00650	Bacteria	0.13
1110	1294025	Cellulosilyticum ruminicola JCM 14822	Bacteria	0.13
1111	37733.7	Prunus necrotic ringspot virus	Viruses	0.13
1112	393921.1	Porphyromonas crevioricanis strain COT-253	Bacteria	0.13
1113	1496.602	Clostridioides difficile isolate VL_0285	Bacteria	0.13
1114	1943580.1	Pyramidobacter sp. C12-8	Bacteria	0.13
1115	1410663	Megasphaera elsdenii T81	Bacteria	0.13
1116	1236517	Prevotella fusca JCM 17724	Bacteria	0.13
1117	168135.1	Apium virus Y	Viruses	0.13
1118	1739522.1	Haemophilus sp. HMSC068C11	Bacteria	0.13
1119	544580.13	Actinomyces oris strain P6N	Bacteria	0.13
1120	1382366	Lactobacillus plantarum 4_3	Bacteria	0.13
1121	1035185	Streptococcus parasanguinis SK236	Bacteria	0.13
1122	404196.1	Blackberry yellow vein-associated virus	Viruses	0.13
1123	12172.1	Shallot latent virus isolate SW3	Viruses	0.13
1124	28026.4	Bifidobacterium pseudocatenulatum strain CA-05	Bacteria	0.13
1125	686660	Veillonella parvula ATCC 17745	Bacteria	0.13
1126	1679.13	Bifidobacterium longum subsp. longum strain VMKB44	Bacteria	0.13
1127	1122981	Prevotella corporis DSM 18810 = JCM 8529	Bacteria	0.13
1128	469588.1	Bacteroides sp. 2_1_22	Bacteria	0.13
1129	1235558	Herbaspirillum huttiense subsp. putei IAM 15032	Bacteria	0.13
1130	1401244	Methanobrevibacter arboriphilus ANOR1	Archaea	0.13
1131	1261.2	Peptostreptococcus anaerobius strain KA00810	Bacteria	0.13
1132	1408472	Prevotella brevis ATCC 19188	Bacteria	0.13
1133	1579342.1	Streptococcus sp. 343_SSPC	Bacteria	0.13
1134	910312	Porphyromonas asaccharolytica PR426713P-I	Bacteria	0.13
1135	180332.2	Robinsoniella peoriensis isolate 6600698	Bacteria	0.13
1136	1630.2	Kandleria vitulina strain S3b	Bacteria	0.13
1137	1322347	Bifidobacterium longum E18	Bacteria	0.13
1138	936375.1	Mogibacterium sp. CM50	Bacteria	0.12
1139	999422	Prevotella maculosa OT 289	Bacteria	0.12
1140	12056.1	Tobacco necrosis virus D	Viruses	0.12
1141	193121.1	Pea enation mosaic virus-1	Viruses	0.12
1142	1423746	Lactobacillus frumenti DSM 13145	Bacteria	0.12
1143	154288.1	Turicibacter sanguinis strain 2789STDY5834851	Bacteria	0.12
1144	1449896.1	Uncultured phage WW-nAnB strain 2 strain 2	Viruses	0.12
1145	1236521	Porphyromonas macacae JCM 15984	Bacteria	0.12
1146	59201.8	Salmonella enterica subsp. enterica strain ADRDL-LA-5-2014	Bacteria	0.12

1147	1286820	[Clostridium] methoxybenzovorans SR3	Bacteria	0.12
1148	1402207	Lactobacillus ruminis S23	Bacteria	0.12
1149	1121950	Hespellia stercorisuis DSM 15480	Bacteria	0.12
1150	445974	Erysipelatoclostridium ramosum DSM 1402	Bacteria	0.12
1151	727.74	Haemophilus influenzae strain 841_HINF	Bacteria	0.12
1152	1495144.1	methanogenic archaeon ISO4-H5	Archaea	0.12
1153	12319.3	Apple mosaic virus isolate Apple	Viruses	0.12
1154	31504.1	Tobacco ringspot virus satellite RNA	Viruses	0.12
1155	502393.1	Gemella asaccharolytica strain KA00071	Bacteria	0.12
1156	1155766	Enterococcus faecium Aus0004	Bacteria	0.12
1157	11987.1	Melon necrotic spot virus strain MNSV/USA/2016	Viruses	0.11
1158	1105171.1	Bacteroides phage B124-14	Viruses	0.11
1159	1341156	Ruminococcus albus SY3	Bacteria	0.11
1160	1347790	Prevotella intermedia ZT	Bacteria	0.11
1161	1639.215	Listeria monocytogenes strain FDA00009837	Bacteria	0.11
1162	1852379.1	Veillonellaceae bacterium Marseille-P2911	Bacteria	0.11
1163	521393	Actinomyces timonensis DSM 23838	Bacteria	0.11
1164	1401078	Prevotella buccalis DNF00985	Bacteria	0.11
1165	216816.14	Bifidobacterium longum isolate Bifido_04	Bacteria	0.11
1166	562981	Gemella haemolysans M341	Bacteria	0.11
1167	1232449.1	Clostridiales bacterium VE202-08	Bacteria	0.11
1168	563008	Prevotella oris C735	Bacteria	0.11
1169	1768874.1	Sinapis alba cryptic virus 1 isolate LTBJ	Viruses	0.11
1170	47770.6	Lactobacillus crispatus strain VMC7	Bacteria	0.11
1171	1128111	Veillonella atypica KON	Bacteria	0.11
1172	862965	Haemophilus parainfluenzae T3T1	Bacteria	0.11
1173	1391465	Enterococcus faecium 10/96A	Bacteria	0.11
1174	1122984	Prevotella intermedia ATCC 25611 = DSM 20706	Bacteria	0.11
1175	305.9	Ralstonia solanacearum strain 58_RSOL	Bacteria	0.11
1176	1319815	Cetobacterium somerae ATCC BAA-474	Bacteria	0.11
1177	1712675.1	Turicibacter sp. H121 sp. H121	Bacteria	0.11
1178	469605	Fusobacterium gonidiaformans 3-1-5R	Bacteria	0.11
1179	180332.1	Robinsoniella peoriensis strain WT	Bacteria	0.11
1180	1190620.1	Atopobium sp. ICM42b	Bacteria	0.11
1181	397290.1	Lachnospiraceae bacterium A2	Bacteria	0.11
1182	1423782	Lactobacillus panis DSM 6035	Bacteria	0.10
1183	887929	Pseudoramibacter alactolyticus ATCC 23263	Bacteria	0.10
1184	1280686.1	Butyrivibrio sp. MC2013	Bacteria	0.10
1185	585506	Weissella paramesenteroides ATCC 33313	Bacteria	0.10
1186	1423769	Lactobacillus manihotivorans DSM 13343 = JCM 12514	Bacteria	0.10
1187	839.3	Prevotella ruminicola strain D31d	Bacteria	0.10
1188	367121.1	Grapevine leafroll-associated virus 10	Viruses	0.10
1189	1685.22	Bifidobacterium breve strain LMC520	Bacteria	0.10
1190	167634.1	Grapevine rootstock stem lesion associated virus	Viruses	0.10
1191	1122978	Prevotella albensis DSM 11370 = JCM 12258	Bacteria	0.10
1192	626369	Granulicatella elegans ATCC 700633	Bacteria	0.10
1193	1595998.12	Human smacovirus 1 isolate France/3/2009/4191	Viruses	0.10

1194	1122991	Prevotella shahii DSM 15611 = JCM 12083	Bacteria	0.10
1195	12175.4	Apple chlorotic leaf spot virus strain AC-ind	Viruses	0.10
1196	873127	Enterococcus faecium E4453	Bacteria	0.10
1197	1054217.1	Thermoplasmatales archaeon BRNA1	Archaea	0.10
1198	1127131	Weissella confusa LBAE C39-2	Bacteria	0.10
1199	548480	Bifidobacterium longum subsp. longum ATCC 55813	Bacteria	0.10
1200	318464.1	Clostridium sulfidigenes strain 113A	Bacteria	0.10
1201	649743	Actinomyces sp. oral taxon 848 str. F0332	Bacteria	0.10
1202	1384065	Ruminococcus albus AD2013	Bacteria	0.10
1203	33945.1	Enterococcus avium strain 639_EFCM	Bacteria	0.10
1204	39681.1	Asparagus virus 2	Viruses	0.10
1205	936591.1	Veillonella sp. ICM51a	Bacteria	0.10
1206	1218148	Salmonella enterica subsp. enterica serovar Typhimurium str. STm8	Bacteria	0.10
1207	119219.2	Cupriavidus metallidurans strain NA4	Bacteria	0.10
1208	1423801	Lactobacillus satsumensis DSM 16230 = JCM 12392	Bacteria	0.10
1209	1496.628	Clostridioides difficile isolate VL_0086	Bacteria	0.09
1210	35350.1	Apple stem pitting virus isolate PM8	Viruses	0.09
1211	91753.2	Cucurbit aphid-borne yellows virus	Viruses	0.09
1212	1581114.1	Enterococcus sp. HMSC05C03	Bacteria	0.09
1213	99179.1	Bacteroides phage B40-8	Viruses	0.09
1214	35350.2	Apple stem pitting virus isolate YT	Viruses	0.09
1215	1702221.2	Faecalibaculum rodentium strain Alo17	Bacteria	0.09
1216	983966	Cyberlindnera jadinii NRRL Y-1542	Eukaryota	0.09
1217	35350.1	Apple stem pitting virus isolate Hannover	Viruses	0.09
1218	288000.3	Bradyrhizobium sp. BTAi1 sp. BTAi1	Bacteria	0.09
1219	1321772	Aggregatibacter sp. oral taxon 458 str. W10330	Bacteria	0.09
1220	203168.1	Grapevine leafroll-associated virus 6	Viruses	0.09
1221	90410.1	Streptococcus phage DT1	Viruses	0.09
1222	1318.7	Streptococcus parasanguinis strain 392_SPAR	Bacteria	0.09
1223	82135.2	Atopobium vaginae strain CMW7778A	Bacteria	0.09
1224	1561964.1	Methanosphaera sp. WGK6	Archaea	0.09
1225	28026.2	C29	Bacteria	0.09
1226	479713	Primula malacoides virus China/Mar2007	Viruses	0.09
1227	29272.3	Turnip vein-clearing virus	Viruses	0.09
1228	1588751.1	Tissierellia bacterium KA00581	Bacteria	0.09
1229	1282664	Streptococcus oralis subsp. tigurinus AZ_3a	Bacteria	0.09
1230	927694	Leuconostoc inhae KCTC 3774	Bacteria	0.09
1231	348151.1	Lactobacillus siliginis strain DSM	Bacteria	0.09
1232	1165892	Leuconostoc gelidum subsp. gasicomitatum KG16-1	Bacteria	0.09
1233	585530	Brevibacterium mcbrellneri ATCC 49030	Bacteria	0.09
1234	1121126	Brochothrix thermosphacta DSM 20171 = FSL F6-1036	Bacteria	0.09
1235	1430896	Haemophilus parahaemolyticus G321	Bacteria	0.09
1236	1121132	Butyrivibrio hungatei DSM 14810	Bacteria	0.08

1237	11612	Impatiens necrotic spot virus	Viruses	0.08
1238	1235835.1	Anaerotruncus sp. G3(2012)	Bacteria	0.08
1239	1661.1	Trueperella pyogenes strain 331_TPYO	Bacteria	0.08
1240	134358.2	Westerdykella cylindrica strain ATCC	Eukaryota	0.08
1241	1267.2	Clostridium ventriculi strain 2789STDY5834858	Bacteria	0.08
1242	1605.2	Lactobacillus animalis strain 381-IL-28	Bacteria	0.08
1243	196400.1	Grapevine rupestris stem pitting-associated virus isolate SK704-B	Viruses	0.08
1244	470.537	Acinetobacter baumannii strain XH803	Bacteria	0.08
1245	907.1	Megasphaera elsdenii strain 24-50	Bacteria	0.08
1246	28347.4	Apple stem grooving virus isolate M219-3	Viruses	0.08
1247	180957.7	Pectobacterium carotovorum subsp. brasiliense strain BD255	Bacteria	0.08
1248	190721.2	Ralstonia insidiosa strain ATCC	Bacteria	0.08
1249	1176736.1	Pitaya virus X isolate P37	Viruses	0.08
1250	1755753.1	Penicillium aurantiogriseum foetidus-like virus	Viruses	0.08
1251	1673726.1	Clostridiales bacterium SIT11	Bacteria	0.08
1252	1111133.1	Peptoniphilus sp. BV3AC2	Bacteria	0.08
1253	1123303	Streptococcus ferus DSM 20646	Bacteria	0.08
1254	28901.27	Salmonella enterica strain NGUA09	Bacteria	0.08
1255	1352.151	Enterococcus faecium isolate Hp_21-21	Bacteria	0.08
1256	1226531	Saccharomyces cerevisiae virus L-A-lus	Viruses	0.08
1257	1235802	Eubacterium plexicaudatum ASF492	Bacteria	0.08
1258	1324352.1	Chryseobacterium gallinarum strain DSM	Bacteria	0.08
1259	485724.1	Melon severe mosaic tospovirus isolate VE440-A	Viruses	0.08
1260	1203556.1	Actinomyces sp. HPA0247	Bacteria	0.08
1261	1357399	Helicobacter canis NCTC 12740	Bacteria	0.08
1262	1840217.1	Candidatus Arthromitus sp. SFB-turkey isolate UMNCA01	Bacteria	0.08
1263	796944	Oribacterium asaccharolyticum ACB7	Bacteria	0.08
1264	12615.8	Cherry leaf roll virus isolate Olm1	Viruses	0.08
1265	67761.3	Cowpea mild mottle virus isolate CPMMV:BR:GO:01:1	Viruses	0.08
1266	1401068	Prevotella bivia DNF00320	Bacteria	0.08
1267	1660.1	Actinomyces odontolyticus strain XH001	Bacteria	0.07
1268	35350.8	Apple stem pitting virus isolate HB-HN1	Viruses	0.07
1269	64003.5	93/955	Viruses	0.07
1270	1118059	Kallipyga massiliensis ph2	Bacteria	0.07
1271	936574.1	Shuttleworthia sp. MSX8B	Bacteria	0.07
1272	37733.5	Prunus necrotic ringspot virus isolate ChrYL	Viruses	0.07
1273	1385512	Pontibacillus litoralis JSM 072002	Bacteria	0.07
1274	12143.1	Cucumber necrosis virus	Viruses	0.07
1275	1407052	Lactobacillus fermentum L930BB	Bacteria	0.07
1276	202566.1	Cherry rasp leaf virus	Viruses	0.07
1277	1121111	Bifidobacterium thermacidophilum subsp. thermacidophilum DSM 15837	Bacteria	0.07
1278	328061.2	Radish mosaic virus	Viruses	0.07
1279	1335.1	Streptococcus equinus strain ICDDRB-NRC-S6	Bacteria	0.07

1280	37734.4	Enterococcus casseliflavus strain PAVET15	Bacteria	0.07
1281	28347.8	Apple stem grooving virus isolate HPKu-2	Viruses	0.07
1282	1720298.1	Peptoniphilus phoceensis strain SIT15	Bacteria	0.07
1283	1339293	Bacteroides fragilis str. 1007-1-F #8	Bacteria	0.07
1284	1280670.1	Butyrivibrio sp. AD3002	Bacteria	0.07
1285	1679.3	Bifidobacterium longum subsp. longum strain MC-42	Bacteria	0.07
1286	888057	Aggregatibacter segnis ATCC 33393	Bacteria	0.07
1287	1402208	Lactobacillus ruminis DPC 6832	Bacteria	0.07
1288	888062	Dialister micraerophilus DSM 19965	Bacteria	0.07
1289	1435051	Bifidobacterium moukalabense DSM 27321	Bacteria	0.07
1290	11008.1	Saccharomyces cerevisiae virus L-A	Viruses	0.07
1291	997898	Clostridium butyricum 60E.3	Bacteria	0.07
1292	467210.1	Lachnoanaerobaculum saburreum strain DNF00896	Bacteria	0.07
1293	111105.1	Porphyromonas gulae strain COT-052	Bacteria	0.07
1294	679192	Bulleidia extructa W1219	Bacteria	0.07
1295	767453	Lactobacillus fermentum F-6	Bacteria	0.07
1296	294747	Candida tropicalis MYA-3404	Eukaryota	0.07
1297	326941.1	Raspberry leaf mottle virus isolate HCRL	Viruses	0.07
1298	697329	Ruminococcus albus 7 = DSM 20455	Bacteria	0.07
1299	33029.1	Anaerococcus hydrogenalis strain MJR7738A	Bacteria	0.07
1300	469615	Fusobacterium gonidiaformans ATCC 25563	Bacteria	0.07
1301	936549.1	Actinomyces sp. ICM54	Bacteria	0.07
1302	879308	Peptoniphilus sp. oral taxon 375 str. F0436	Bacteria	0.07
1303	54067.1	Xylophilus ampelinus strain CCH5-B3	Bacteria	0.07
1304	1230730.1	Tissierellia bacterium S5-A11	Bacteria	0.07
1305	12433.2	Garlic virus A isolate WA7	Viruses	0.07
1306	1697797.1	Actinobacteria bacterium UC5.1-1B11	Bacteria	0.07
1307	1695.2	Bifidobacterium longum subsp. suis strain BSM11-5	Bacteria	0.07
1308	1348632	Lactococcus plantarum NBRC 100936	Bacteria	0.07
1309	253700.1	Schlumbergera virus X	Viruses	0.07
1310	1347393.1	Bacteroides neonati strain MS4	Bacteria	0.07
1311	663278	Ethanoligenens harbinense YUAN-3	Bacteria	0.07
1312	1437611	Bifidobacterium saeculare DSM 6531 = LMG 14934	Bacteria	0.07
1313	1211814	Bacillus massilioanorexius AP8	Bacteria	0.07
1314	1496.534	Clostridioides difficile isolate VL_0083	Bacteria	0.06
1315	1739371.1	Streptococcus sp. HMSC064H09	Bacteria	0.06
1316	33763.1	Peanut mottle virus	Viruses	0.06
1317	1161744	Bifidobacterium longum subsp. longum 1-6B	Bacteria	0.06
1318	936589.1	Veillonella sp. AS16	Bacteria	0.06
1319	904306	Streptococcus vestibularis F0396	Bacteria	0.06
1320	537971	Helicobacter cinaedi CCUG 18818 = ATCC BAA-847	Bacteria	0.06
1321	1080349.1	Saccharomyces eubayanus strain FM1318	Eukaryota	0.06
1322	1265.3	Ruminococcus flavefaciens strain YRD2003	Bacteria	0.06

1323	985691	Sapovirus Hu/GI.2/BR-DF01/BRA/2009	Viruses	0.06
1324	12430.3	Garlic virus D isolate GarVD-SW10	Viruses	0.06
1325	1215061	Clostridioides difficile E10	Bacteria	0.06
1326	1350473	Bifidobacterium longum subsp. longum 17-1B	Bacteria	0.06
1327	1299998.1	Olsenella scatoligenes strain SK9K4	Bacteria	0.06
1328	1118058.1	Actinomyces sp. ph3	Bacteria	0.06
1329	1046402.2	Potato virus H isolate YN	Viruses	0.06
1330	1127690	Actinomyces sp. oral taxon 181 str. F0379	Bacteria	0.06
1331	696281	Desulfotomaculum ruminis DSM 2154	Bacteria	0.06
1332	866774	Atopobium vaginae PB189-T1-4	Bacteria	0.06
1333	866776	Veillonella atypica ACS-049-V-Sch6	Bacteria	0.06
1334	1122151	Lactobacillus paralimentarius DSM 13238 = JCM 10415	Bacteria	0.06
1335	1410616.1	Pseudobutyrivibrio sp. MD2005	Bacteria	0.06
1336	37734.2	Enterococcus casseliflavus strain NLAE-zl-G268	Bacteria	0.06
1337	1679.12	W11	Bacteria	0.06
1338	36874.1	Porphyromonas cangingivalis strain COT-109	Bacteria	0.06
1339	1852378.1	Veillonellaceae bacterium Marseille-P2974	Bacteria	0.06
1340	679200	Johnsonella ignava ATCC 51276	Bacteria	0.06
1341	767031	Prevotella denticola F0289	Bacteria	0.06
1342	1914544	Norovirus Hu/USA/2014/GI.P7_GI.7/GA5043	Viruses	0.06
1343	284592	Debaryomyces hansenii CBS767	Eukaryota	0.06
1344	1930605	Saccharomyces cerevisiae virus L-BC-2	Viruses	0.06
1345	1774276.1	Hordeum vulgare endornavirus	Viruses	0.06
1346	1235798.1	Dorea sp. 5-2	Bacteria	0.06
1347	537937	Bifidobacterium longum subsp. infantis CCUG 52486	Bacteria	0.06
1348	1235790.1	Eubacterium sp. 14-2	Bacteria	0.06
1349	31713.1	Lettuce infectious yellows virus	Viruses	0.06
1350	208084.1	Grapevine Algerian latent virus	Viruses	0.06
1351	253700.3	Schlumbergera virus X isolate Palma-PE	Viruses	0.06
1352	253700.2	Schlumbergera virus X isolate nopal	Viruses	0.06
1353	1175296	Methanomassiliicoccus luminyensis B10	Archaea	0.06
1354	1401079	Mogibacterium timidum ATCC 33093	Bacteria	0.06
1355	1203602	Atopobium sp. oral taxon 199 str. F0494	Bacteria	0.06
1356	33036.1	Anaerococcus tetradius strain MJR8151	Bacteria	0.06
1357	1423799	Lactobacillus salivarius DSM 20555 = ATCC 11741	Bacteria	0.06
1358	1358418.1	Sinorhizobium sp. GL28	Bacteria	0.06
1359	1401066	Prevotella amnii DNF00058	Bacteria	0.06
1360	1739537.1	Anaerococcus sp. HMSC075B03	Bacteria	0.06
1361	1423764	Lactobacillus kefiri DSM 20587 = JCM 5818	Bacteria	0.06
1362	196400.1	Grapevine rupestris stem pitting-associated virus strain Syrah	Viruses	0.06
1363	11987.4	Melon necrotic spot virus isolate N	Viruses	0.06
1364	35350.9	Apple stem pitting virus isolate PB66	Viruses	0.06
1365	1423815	Lactobacillus versmoldensis DSM 14857 = KCTC 3814	Bacteria	0.06

1366	1423783	Lactobacillus pantheris DSM 15945 = JCM 12539 = NBRC 106106	Bacteria	0.06
1367	1302.12	Streptococcus gordonii strain MB666	Bacteria	0.06
1368	888826	Campylobacter upsaliensis JV21	Bacteria	0.06
1369	1579341.1	Streptococcus sp. 400_SSPC	Bacteria	0.06
1370	348449.1	Raphanus sativus cryptic virus 1	Viruses	0.06
1371	1161412.1	Prevotella sp. ICM33	Bacteria	0.06
1372	1613.2	Lactobacillus fermentum	Bacteria	0.06
1373	1000568.1	Megasphaera sp. UPII 199-6	Bacteria	0.06
1374	12430.2	Garlic virus D isolate GarVD-SW9	Viruses	0.06
1375	1095733	Streptococcus parasanguinis F0449	Bacteria	0.06
1376	379892.1	Passiflora latent carlavirus	Viruses	0.06
1377	1442158	Saccharomyces cerevisiae virus L-A-2	Viruses	0.06
1378	1123250	Selenomonas bovis DSM 23594	Bacteria	0.06
1379	1261640.1	Eubacterium sp. 68-3-10	Bacteria	0.06
1380	42004.6	Leek yellow stripe virus isolate LYSV-MG	Viruses	0.06
1381	42882.2	Cherry virus A isolate ChYT52	Viruses	0.06
1382	158787.2	Bifidobacterium scardovii strain LMG	Bacteria	0.06
1383	60456	Iris yellow spot virus	Viruses	0.06
1384	553175	Porphyromonas endodontalis ATCC 35406	Bacteria	0.06
1385	1123754.1	nopal	Viruses	0.06
1386	1297865.1	Bradyrhizobium sp. OHSU_III	Bacteria	0.06
1387	2702.11	Gardnerella vaginalis strain CMW7778B	Bacteria	0.06
1388	562.547	Escherichia coli strain LM17584/1	Bacteria	0.06
1389	767100	Parvimonas sp. oral taxon 110 str. F0139	Bacteria	0.06
1390	1151384	Clostridioides difficile Y266	Bacteria	0.06
1391	1686287.1	Kallipyga gabonensis strain GM4	Bacteria	0.06
1392	1276.1	Kytococcus sedentarius strain 262_KSED	Bacteria	0.06
1393	1080072.1	Streptococcus dentasini	Bacteria	0.06
1394	105219.3	Ralstonia mannitolilytica strain SN83A39	Bacteria	0.06
1395	31537.1	Lactococcus phage c2	Viruses	0.06
1396	1852373.1	Murdochiella sp. Marseille-P2341 strain Marseille-P2341T	Bacteria	0.06
1397	1469950.1	Robinsoniella sp. KNHs210	Bacteria	0.06
1398	72000.1	Kocuria rhizophila strain 14ASP	Bacteria	0.06
1399	1525173.1	Human circovirus VS6600022	Viruses	0.06
1400	134605.1	Fusobacterium equinum strain CMW8396	Bacteria	0.06
1401	234601	Sapovirus Mc10	Viruses	0.06
1402	626523	Shuttleworthia satelles DSM 14600	Bacteria	0.06
1403	936550.1	Atopobium sp. BS2	Bacteria	0.06
1404	1348.8	Streptococcus parauberis strain SP-llh	Bacteria	0.06
1405	425279.2	Rehmannia mosaic virus	Viruses	0.06
1406	1777865.1	Weissella sp. DD23	Bacteria	0.06
1407	196375.8	Beet black scorch virus isolate CO	Viruses	0.06
1408	39777.1	Veillonella atypica strain CMW7756B	Bacteria	0.06
1409	655811	Anaerococcus vaginalis ATCC 51170	Bacteria	0.06
1410	1715211.1	Haemophilus sp. HMSC061E01	Bacteria	0.06
1411	45071.2	Legionella parisiensis strain DSM	Bacteria	0.06

1412	1235800.1	Lachnospiraceae bacterium 10-1	Bacteria	0.06
1413	862517	Peptoniphilus duerdenii ATCC BAA-1640	Bacteria	0.06
1414	1711684.1	Hot pepper endornavirus isolate CS	Viruses	0.06
1415	1497954.1	Bacteroidales bacterium KA00344	Bacteria	0.06
1416	1280700	Butyrivibrio fibrisolvens FE2007	Bacteria	0.06
1417	869209	Treponema succinifaciens DSM 2489	Bacteria	0.05
1418	729.7	Haemophilus parainfluenzae strain 901_HPAR	Bacteria	0.05
1419	328396.1	Enterococcus aquimarinus strain DSM	Bacteria	0.05
1420	1232454.1	Clostridiales bacterium VE202-26	Bacteria	0.05
1421	1425364.1	Carrot torradovirus 1 isolate CTV-1_RNA2_H6	Viruses	0.05
1422	396268.1	Lactobacillus secaliphilus strain DSM	Bacteria	0.05
1423	1074482	Bifidobacterium breve DPC 6330	Bacteria	0.05
1424	1190621	Olsenella uli MSTE5	Bacteria	0.05
1425	1121100	Bacteroides pyogenes DSM 20611 = JCM 6294	Bacteria	0.05
1426	1095727.1	Streptococcus sp. SK643	Bacteria	0.05
1427	888832	Prevotella salivae DSM 15606	Bacteria	0.05
1428	595895.1	Drosophila A virus	Viruses	0.05
1429	1265.5	Ruminococcus flavefaciens strain XPD3002	Bacteria	0.05
1430	1265.1	Ruminococcus flavefaciens strain YL228	Bacteria	0.05
1431	1405296	Chlamydia suis MD56	Bacteria	0.05
1432	1654927.1	Klebsiella phage PKP126	Viruses	0.05
1433	1074494	Streptococcus salivarius M18	Bacteria	0.05
1434	12432.1	Garlic virus B isolate Mesi	Viruses	0.05
1435	1739422.1	Streptococcus sp. HMSC065C01	Bacteria	0.05
1436	1520332.1	Blueberry mosaic associated virus	Viruses	0.05
1437	907.3	Megasphaera elsdenii strain YE34	Bacteria	0.05
1438	29363.1	Clostridium paraputrificum strain 373-A1	Bacteria	0.05
1439	1236509	Prevotella dentasini JCM 15908	Bacteria	0.05
1440	866778	Veillonella atypica ACS-134-V-Col7a	Bacteria	0.05
1441	216816.16	Bifidobacterium longum	Bacteria	0.05
1442	1739286.1	Anaerococcus sp. HMSC068A02	Bacteria	0.05
1443	1167628	Actinomyces massiliensis 4401292	Bacteria	0.05
1444	311413.1	Lettuce big-vein associated virus	Viruses	0.05
1445	42882.4	Cherry virus A isolate JK	Viruses	0.05
1446	729.4	Haemophilus parainfluenzae strain 209_HPAR	Bacteria	0.05
1447	739.1	Aggregatibacter segnis strain 933_AAPH	Bacteria	0.05
1448	1122147	Lactobacillus harbinensis DSM 16991	Bacteria	0.05
1449	1284686	Anaerococcus lactolyticus S7-1-13	Bacteria	0.05
1450	1658779.2	Porphyromonadaceae bacterium H1	Bacteria	0.05
1451	12280.8	Tomato ringspot virus isolate Rasp1-2014	Viruses	0.05
1452	936381.1	Selenomonas sp. CM52	Bacteria	0.05
1453	1331064	Escherichia coli CE516	Bacteria	0.05
1454	1123754.2	Rattail cactus necrosis-associated virus	Viruses	0.05
1455	284593	Candida glabrata CBS 138	Eukaryota	0.05
1456	284590	Kluyveromyces lactis NRRL Y-1140	Eukaryota	0.05
1457	682370.1	Streptococcus phage Alq132	Viruses	0.05
1458	729.16	Haemophilus parainfluenzae strain 65114	Bacteria	0.05
1459	1267000	Mycoplasma hominis ATCC 27545	Bacteria	0.05

1460	768724	Peptoniphilus sp. oral taxon 836 str. F0141	Bacteria	0.05
1461	84135.1	Gemella sanguinis strain 1094_BTHU	Bacteria	0.05
1462	553207	Corynebacterium matruchotii ATCC 14266	Bacteria	0.05
1463	1323524.1	Red clover cryptic virus 2 isolate IPP_Nemaro	Viruses	0.05
1464	1719140.1	Klebsiella phage vB_KpnM_KB57	Viruses	0.05
1465	173976.1	Cycas necrotic stunt virus	Viruses	0.05
1466	1122979	Prevotella amnii DSM 23384 = JCM 14753	Bacteria	0.05
1467	1351755	Clostridium chauvoei JF4335	Bacteria	0.05
1468	1715086.1	Streptococcus sp. HMSC078H03	Bacteria	0.05
1469	290314	Sapovirus C12	Viruses	0.05
1470	1121321	Asaccharospora irregularis DSM 2635	Bacteria	0.05
1471	876478.1	Tepidiphilus thermophilus strain JCM	Bacteria	0.05
1472	471858.1	Helicobacter magdeburgensis strain MIT	Bacteria	0.05
1473	397287.1	Lachnospiraceae bacterium 28-4	Bacteria	0.05
1474	35281.1	Paprika mild mottle virus strain israeli	Viruses	0.05
1475	767029	Pseudopropionibacterium propionicum F0230a	Bacteria	0.05
1476	1840518.1	Gardnerella sp. 30-4	Bacteria	0.05
1477	87541.1	Aerococcus christensenii strain KA00635	Bacteria	0.05
1478	1051074	Streptococcus thermophilus JIM 8232	Bacteria	0.05
1479	1035817	Bifidobacterium longum subsp. longum KACC 91563	Bacteria	0.05
1480	1675609.1	Klebsiella phage Sushi	Viruses	0.05
1481	1496.3	Clostridioides difficile isolate VL_0125	Bacteria	0.05
1482	525256	Atopobium vaginae DSM 15829	Bacteria	0.05
1483	457388.1	Parabacteroides sp. 2_1_7	Bacteria	0.05
1484	664639.1	Kocuria salsicia	Bacteria	0.05
1485	1006000	Kluyvera ascorbata ATCC 33433	Bacteria	0.05
1486	883113	Facklamia languida CCUG 37842	Bacteria	0.05
1487	1505.39	Paeniclostridium sordellii strain R32462	Bacteria	0.05
1488	1601.1	Lactobacillus agilis	Bacteria	0.05
1489	1287474	Atopobium parvulum DNF00906	Bacteria	0.05
1490	883062	Bifidobacterium bifidum S17	Bacteria	0.05
1491	1384078.1	Prevotella sp. DNF00663	Bacteria	0.05
1492	42478.1	Saccharomyces cerevisiae virus L-BC (La)	Viruses	0.05
1493	905.2	Acidaminococcus fermentans strain WCC6	Bacteria	0.05
1494	71030.1	Chayote mosaic virus	Viruses	0.05
1495	883112	Facklamia ignava CCUG 37419	Bacteria	0.05
1496	1744.1	Propionibacterium freudenreichii	Bacteria	0.04
1497	1131702.1	Persea americana endornavirus 1	Viruses	0.04
1498	33763.2	Peanut mottle virus isolate Habin	Viruses	0.04
1499	55951.9	Grapevine leafroll-associated virus 3 clone 3	Viruses	0.04
1500	591001	Acidaminococcus fermentans DSM 20731	Bacteria	0.04
1501	273677.2	Microbacterium oleivorans strain Wellendorf	Bacteria	0.04
1502	392504.1	Turnip ringspot virus isolate B	Viruses	0.04
1503	1592930.1	Yam latent virus isolate SG1	Viruses	0.04
1504	1403344	Xylella fastidiosa Mul-MD	Bacteria	0.04
1505	1423770	Lactobacillus mindensis DSM 14500	Bacteria	0.04
1506	1121102	Campylobacter ureolyticus DSM 20703	Bacteria	0.04

1507	1674942.1	Caenibacillus caldisaponilyticus strain B157	Bacteria	0.04
1508	137591.1	Weissella cibaria strain FBL5	Bacteria	0.04
1509	1457183	Bifidobacterium longum subsp. infantis EK3	Bacteria	0.04
1510	4909.1	Pichia kudriavzevii	Eukaryota	0.04
1511	1120996	Anaerosporobacter mobilis DSM 15930	Bacteria	0.04
1512	944565	Parvimonas sp. oral taxon 393 str. F0440	Bacteria	0.04
1513	1318.15	Streptococcus parasanguinis strain 512_SPAR	Bacteria	0.04
1514	1321823	Porphyromonas gingivalis W4087	Bacteria	0.04
1515	360104	Campylobacter concisus 13826	Bacteria	0.04
1516	1323525.1	White clover cryptic virus 2 isolate IPP_Lirepa	Viruses	0.04
1517	999411	Clostridium colicanis 209318	Bacteria	0.04
1518	1862960.1	Lactococcus phage M5938	Viruses	0.04
1519	1597976.1	Enterococcus phage EFDG1	Viruses	0.04
1520	61592.1	Corynebacterium durum strain CCH6-D9	Bacteria	0.04
1521	907.2	Megasphaera elsdenii strain J1	Bacteria	0.04
1522	1423750	Lactobacillus ghanensis DSM 18630	Bacteria	0.04
1523	45634.1	Streptococcus cristatus strain DD08	Bacteria	0.04
1524	1739356.1	Anaerococcus sp. HMSC065G05	Bacteria	0.04
1525	638301	Granulicatella adiacens ATCC 49175	Bacteria	0.04
1526	298.2	Pseudomonas marginalis strain BS2952	Bacteria	0.04
1527	1165092.1	Lachnospiraceae bacterium JC7	Bacteria	0.04
1528	97139.1	Clostridium sp. ASF502	Bacteria	0.04
1529	28347.9	Apple stem grooving virus isolate HH	Viruses	0.04
1530	1410676	Succinivibrio dextrinosolvens H5	Bacteria	0.04
1531	1579339.1	Streptococcus sp. 449_SSPC	Bacteria	0.04
1532	1739413.1	Alloscardovia sp. HMSC034E08	Bacteria	0.04
1533	1433844.1	Prevotella sp. HJM029	Bacteria	0.04
1534	1336241	Eubacterium xylanophilum ATCC 35991	Bacteria	0.04
1535	42004.2	Leek yellow stripe virus isolate SW8	Viruses	0.04
1536	1316412.1	Streptococcus sp. HSISS3	Bacteria	0.04
1537	525254	Anaerococcus lactolyticus ATCC 51172	Bacteria	0.04
1538	888052	Actinomyces sp. oral taxon 180 str. F0310	Bacteria	0.04
1539	1235799.1	Lachnospiraceae bacterium 3-2	Bacteria	0.04
1540	1214168	Streptococcus suis 88-1861	Bacteria	0.04
1541	1805472.1	Clostridium sp. Marseille-P2434 sp. Marseille-P2434	Bacteria	0.04
1542	1860161.1	Streptococcus sp. CCUG 49591	Bacteria	0.04
1543	12275.4	Tomato black ring virus isolate TBRV-P1	Viruses	0.04
1544	1815509.1	Bacillus phage AR9	Viruses	0.04
1545	1445607	Bacteroides reticulotermitis JCM 10512	Bacteria	0.04
1546	1122949	Peptoniphilus lacrimalis DSM 7455	Bacteria	0.04
1547	1158603	Enterococcus flavescens ATCC 49996	Bacteria	0.04
1548	1496.14	Clostridioides difficile strain CD8-15	Bacteria	0.04
1549	1739452.1	Haemophilus sp. HMSC073C03	Bacteria	0.04
1550	1682.4	Bifidobacterium longum subsp. infantis strain TPY12-1	Bacteria	0.04
1551	186189.1	Xylanimonas cellulosilytica strain 352_XCEL	Bacteria	0.04
1552	306264	Campylobacter upsaliensis RM3195	Bacteria	0.04

1553	212365.1	Bifidobacterium thermacidophilum subsp. porcinum strain LMG	Bacteria	0.04
1554	197614.4	Streptococcus pasteurianus strain GED7275A	Bacteria	0.04
1555	1101373.1	Tepidimonas fonticaldi strain PL17	Bacteria	0.04
1556	1496.439	Clostridioides difficile strain VRECD0155	Bacteria	0.04
1557	1423718	Lactobacillus agilis DSM 20509	Bacteria	0.04
1558	213633.2	Providence virus	Viruses	0.04
1559	28026.8	Bifidobacterium pseudocatenulatum strain CECT	Bacteria	0.04
1560	997893	[Clostridium] bolteae 90A5	Bacteria	0.04
1561	1122993	Prevotella veroralis DSM 19559 = JCM 6290	Bacteria	0.04
1562	1930298.1	Chicken stool-associated gemycircularvirus strain RS/BR/2015	Viruses	0.04
1563	1679.16	Bifidobacterium longum subsp. longum strain AH1206	Bacteria	0.04
1564	216816.6	Bifidobacterium longum isolate Bifido_06	Bacteria	0.04
1565	12558.1	Sesbania mosaic virus	Viruses	0.04
1566	12041.1	Bean leafroll virus strain Manfredi	Viruses	0.04
1567	399795	Comamonas testosteroni KF-1	Bacteria	0.04
1568	277.1	Meiothermus ruber strain TC-1	Bacteria	0.04
1569	1122219	Megasphaera cerevisiae DSM 20462	Bacteria	0.04
1570	1282.6	Staphylococcus epidermidis strain MF1789	Bacteria	0.04
1571	1280688	Pseudobutyrivibrio ruminis CF1b	Bacteria	0.04
1572	890402	Bifidobacterium longum subsp. longum BBMN68	Bacteria	0.04
1573	11983.2	Norwalk virus	Viruses	0.04
1574	1384082.1	Veillonellaceae bacterium DNF00751	Bacteria	0.04
1575	1794912.1	Anaerosporomusa subterranea strain RU4	Bacteria	0.04
1576	1454376	Bifidobacterium pseudocatenulatum IPLA36007	Bacteria	0.04
1577	1392486.1	Prevotella sp. HUN102	Bacteria	0.04
1578	546271	Selenomonas sputigena ATCC 35185	Bacteria	0.04
1579	306902	Clavispora lusitaniae ATCC 42720	Eukaryota	0.04
1580	879301	Lactobacillus iners LEAF 2053A-b	Bacteria	0.04
1581	1914543	Norovirus Hu/USA/2011/GI.P7_GI.7/CS5567	Viruses	0.04
1582	29385.22	Staphylococcus saprophyticus strain JB027	Bacteria	0.04
1583	1161417.1	Streptococcus sp. SR4	Bacteria	0.04
1584	1352.198	Enterococcus faecium strain E161	Bacteria	0.04
1585	1339350	Bacteroides vulgatus str. 3775 SL(B) 10 (iv)	Bacteria	0.04
1586	54005.1	Peptoniphilus harei strain CMW7756A	Bacteria	0.04
1587	39397.1	Candida sake strain CBS	Eukaryota	0.04
1588	378833.2	Sowbane mosaic virus isolate SoMV-WA	Viruses	0.04
1589	1408415	Acholeplasma equifetale ATCC 29724	Bacteria	0.04
1590	1408440	Gemella sanguinis ATCC 700632	Bacteria	0.04
1591	1739381.1	Streptococcus sp. HMSC072D03	Bacteria	0.04
1592	90371.51	Salmonella enterica subsp. enterica serovar Typhimurium strain CFSAN033866	Bacteria	0.04
1593	49266.1	Fucus vesiculosus	Eukaryota	0.04
1594	529.5	Ochrobactrum anthropi strain OAB	Bacteria	0.04
1595	28901.23	Salmonella enterica strain NGUA11	Bacteria	0.04
1596	575612	Prevotella melaninogenica D18	Bacteria	0.04

1597	12317.18	Tobacco streak virus isolate 2334	Viruses	0.04
1598	1870984.1	Anaerococcus mediterraneensis strain Marseille-P2765T sp. Marseille-P2765	Bacteria	0.04
1599	47736.1	Carrot mottle mimic virus	Viruses	0.04
1600	225992.1	Comamonas kerstersii strain J29	Bacteria	0.04
1601	12263.4	Squash mosaic virus strain Kimble	Viruses	0.04
1602	888743	Prevotella multiformis DSM 16608	Bacteria	0.03
1603	95609.1	Herbaspirillum sp. B39	Bacteria	0.03
1604	1458705	Norovirus Hu/GII.6/HS245/2010/USA	Viruses	0.03
1605	1756285.1	Maize associated totivirus isolate EC_Portoviejo	Viruses	0.03
1606	1121947	Helcococcus sueciensis DSM 17243	Bacteria	0.03
1607	62059.1	Shallot yellow stripe virus	Viruses	0.03
1608	1304.19	Streptococcus salivarius strain 37-09	Bacteria	0.03
1609	112436.1	Celery mosaic virus	Viruses	0.03
1610	1118057	Peptoniphilus grossensis ph5	Bacteria	0.03
1611	1435146.1	Morganella sp. EGD-HP17	Bacteria	0.03
1612	1235479.1	Pelosinus sp. HCF1	Bacteria	0.03
1613	1294024	Calditerricola satsumensis JCM 14719	Bacteria	0.03
1614	1685.9	Bifidobacterium breve strain BR-10	Bacteria	0.03
1615	1161918	Brachyspira pilosicoli WesB	Bacteria	0.03
1616	1681.5	Bifidobacterium bifidum strain LMG	Bacteria	0.03
1617	12139.3	Southern bean mosaic virus isolate Sao	Viruses	0.03
1618	12450.1	Saccharomyces cerevisiae killer virus M1	Viruses	0.03
1619	1267.1	Clostridium ventriculi	Bacteria	0.03
1620	1352.164	Enterococcus faecium strain E3	Bacteria	0.03
1621	5353.1	Lentinula edodes	Eukaryota	0.03
1622	1859694.1	Haemophilus sp. CCUG 66565	Bacteria	0.03
1623	883092	Lactobacillus crispatus FB077-07	Bacteria	0.03
1624	111015.1	Actinomyces radicidentis strain CCUG	Bacteria	0.03
1625	85154.1	Streptococcus phage O1205	Viruses	0.03
1626	679937	Bacteroides coprosuis DSM 18011	Bacteria	0.03
1627	1496.503	Clostridioides difficile strain VRECD0027	Bacteria	0.03
1628	253701.2	Zygocactus virus X	Viruses	0.03
1629	253701.1	Zygocactus virus X isolate P39	Viruses	0.03
1630	243563.1	Strawberry necrotic shock virus isolate 840	Viruses	0.03
1631	1120988	Anaerobiospirillum succiniciproducens DSM 6400	Bacteria	0.03
1632	2748.1	Carnobacterium divergens isolate CDIV41	Bacteria	0.03
1633	1339280	Bacteroides fragilis str. 2-F-2 #4	Bacteria	0.03
1634	1141139.1	Enterobacteria phage vB_EcoP_ACG-C91	Viruses	0.03
1635	1624.2	Lactobacillus salivarius strain 778_LSAL	Bacteria	0.03
1636	458233	Macrococcus caseolyticus JCSC5402	Bacteria	0.03
1637	1111734	4900	Bacteria	0.03
1638	1581148.1	Clostridium sp. HMSC19A10	Bacteria	0.03
1639	33037.1	Anaerococcus vaginalis strain PH9	Bacteria	0.03
1640	257758.5	Streptococcus pseudopneumoniae strain 315_SPSE	Bacteria	0.03
1641	419005.1	Prevotella amnii strain DNF00307	Bacteria	0.03
1642	1307833	Tannerella forsythia KS16	Bacteria	0.03

1643	883165	Campylobacter ureolyticus ACS-301-V-Sch3b	Bacteria	0.03
1644	729.6	Haemophilus parainfluenzae strain CCUG	Bacteria	0.03
1645	72539.1	Physalis mottle virus	Viruses	0.03
1646	908338	Peptoniphilus harei ACS-146-V-Sch2b	Bacteria	0.03
1647	12313.1	Peanut stunt virus	Viruses	0.03
1648	1287488.1	Prevotella sp. S7 MS 2	Bacteria	0.03
1649	1113547	Salmonella phage 9NA	Viruses	0.03
1650	470.622	Acinetobacter baumannii strain XH545	Bacteria	0.03
1651	578361.3	Soybean yellow mottle mosaic virus isolate New	Viruses	0.03
1652	1141663	Providencia rettgeri Dmel1	Bacteria	0.03
1653	1932006.1	Chicken associated smacovirus strain RS/BR/2015/3	Viruses	0.03
1654	1496.648	Clostridioides difficile isolate VL_0231	Bacteria	0.03
1655	369963	Sapovirus Hu/Ehime475/2004/JP	Viruses	0.03
1656	1120932	Actinotignum schaalii DSM 15541	Bacteria	0.03
1657	388038.1	Cucumber mottle virus	Viruses	0.03
1658	1723382.1	Peptoniphilaceae bacterium FC2	Bacteria	0.03
1659	469553.1	Delftia sp. JD2	Bacteria	0.03
1660	112023.1	Streptococcus phage 7201	Viruses	0.03
1661	1608898.1	Haemophilus sp. HMSC71H05	Bacteria	0.03
1662	1658008.1	Dysgonomonas sp. BGC7	Bacteria	0.03
1663	1379858	Mucispirillum schaedleri ASF457	Bacteria	0.03
1664	1073387	Bacteroides fragilis HMW 615	Bacteria	0.03
1665	698486	Escherichia phage K1-dep(4)	Viruses	0.03
1666	12274.29	Grapevine fanleaf virus isolate GFLV-SDHN	Viruses	0.03
1667	693746	Oscillibacter valericigenes Sjm18-20	Bacteria	0.03
1668	1681.12	Bifidobacterium bifidum strain 156B	Bacteria	0.03
1669	1315956.1	Shigella phage pSf-1	Viruses	0.03
1670	470.475	Acinetobacter baumannii strain IHIT17966	Bacteria	0.03
1671	1123488	Varibaculum cambriense DSM 15806	Bacteria	0.03
1672	1328.3	Streptococcus anginosus strain 557_SANG	Bacteria	0.03
1673	1318.5	Streptococcus parasanguinis strain 886_SPAR	Bacteria	0.03
1674	1318.4	Streptococcus parasanguinis strain 65_SPAR	Bacteria	0.03
1675	1423778	Lactobacillus oligofermentans DSM 15707 = LMG 22743	Bacteria	0.03
1676	298338.1	Lactobacillus phage LP65	Viruses	0.03
1677	1218109	Propionibacterium propionicum NBRC 14587	Bacteria	0.03
1678	1492.4	Clostridium butyricum strain HM-68	Bacteria	0.03
1679	665952	Bacillus smithii 7_3_47FAA	Bacteria	0.03
1680	1689.1	Bifidobacterium dentium strain DE-29	Bacteria	0.03
1681	1225194	Streptococcus mutans B24Sm2	Bacteria	0.03
1682	1526.2	[Clostridium] aminophilum strain KH1P1	Bacteria	0.03
1683	1685.8	Bifidobacterium breve strain BR-21	Bacteria	0.03
1684	1122986	Prevotella maculosa DSM 19339 = JCM 15638	Bacteria	0.03
1685	344022.1	Escherichia virus K1E	Viruses	0.03
1686	35288.1	Grapevine virus A isolate 3138-03	Viruses	0.03
1687	1871031.1	Olsenella sp. Marseille-P3256 sp. Marseille-P3256	Bacteria	0.03
1688	879310	Selenomonas sp. oral taxon 137 str. F0430	Bacteria	0.03

1689	1129191	Klebsiella phage KP36	Viruses	0.03
1690	12470.2	Lucerne transient streak virus	Viruses	0.03
1691	1111121.1	Atopobium sp. BV3Ac4	Bacteria	0.03
1692	1922434.2	Beihai mollusks virus 1 strain WZSLuoI86140	Viruses	0.03
1693	51354.1	Maize chlorotic dwarf virus strain Severe	Viruses	0.03
1694	521000	Providencia rettgeri DSM 1131	Bacteria	0.03
1695	521002	Methanobrevibacter smithii DSM 2374	Archaea	0.03
1696	1027232.1	Groundnut ringspot and Tomato chlorotic spot virus reassortant	Viruses	0.03
1697	1922513.1	HOU234589	Viruses	0.03
1698	1114922	Citrobacter farmeri GTC 1319	Bacteria	0.03
1699	866771	Prevotella disiens FB035-09AN	Bacteria	0.03
1700	988645.1	Raspberry leaf blotch virus	Viruses	0.03
1701	439334.28	Mycobacterium avium subsp. hominissuis strain MAH-E-104-1	Bacteria	0.03
1702	237561	Candida albicans SC5314	Eukaryota	0.03
1703	1121933	Granulicoccus phenolivorans DSM 17626	Bacteria	0.03
1704	1739309.1	Streptococcus sp. HMSC034E03	Bacteria	0.03
1705	1590596.1	Sphingobacterium sp. T2	Bacteria	0.03
1706	1659298.2	Nectarine stem pitting-associated virus isolate NSPaV/12P42	Viruses	0.03
1707	42783	Coxsackievirus A22	Viruses	0.03
1708	1156433.1	Streptococcus sp. I-P16 sp. I-P16	Bacteria	0.03
1709	1496.68	Clostridioides difficile isolate VL_0167	Bacteria	0.03
1710	1304.26	Streptococcus salivarius strain GED7778A	Bacteria	0.03
1711	1718158.1	Enterococcus phage IME-EFm5	Viruses	0.03
1712	1287476	Prevotella bivia DNF00188	Bacteria	0.03
1713	1683.2	Bifidobacterium angulatum strain GT102	Bacteria	0.03
1714	106008.1	Curvibasidium cygneicollum	Eukaryota	0.03
1715	1567484.1	Lactobacillus phage LfeInf	Viruses	0.03
1716	12165.1	Chrysanthemum virus B	Viruses	0.03
1717	290399.2	Arthrobacter sp. FB24	Bacteria	0.03
1718	47715.3	Lactobacillus rhamnosus strain Lrh22	Bacteria	0.03
1719	1891289.1	Sporanaerobacter sp. PP17-6a isolate PP176A	Bacteria	0.03
1720	1496.74	Clostridioides difficile strain CD105KSE1	Bacteria	0.03
1721	879212	Desulfobacter postgatei 2ac9	Bacteria	0.03
1722	1930509.1	Husavirus sp. isolate 16370_59, sp. isolate	Viruses	0.03
1723	529.4	Ochrobactrum anthropi strain ML7	Bacteria	0.03
1724	340.1	Xanthomonas campestris pv. campestris	Bacteria	0.03
1725	1702287.1	Negativicoccus massiliensis strain AT7	Bacteria	0.03
1726	1130798	Lactobacillus mucosae LM1	Bacteria	0.03
1727	12208.1	Pea seed-borne mosaic virus	Viruses	0.02
1728	12431.3	Garlic virus C isolate SW3.3B	Viruses	0.02
1729	469594.1	Bifidobacterium sp. 12_1_47BFAA	Bacteria	0.02
1730	1398.2	Bacillus coagulans strain B4098	Bacteria	0.02
1731	1423780	Lactobacillus otakiensis DSM 19908 = JCM 15040	Bacteria	0.02
1732	1608882.1	Haemophilus sp. HMSC61B11	Bacteria	0.02

1733	596330	Peptoniphilus lacrimalis 315-B	Bacteria	0.02
1734	424716.1	Salmonella phage Vi II-E1	Viruses	0.02
1735	483913.1	Bacillus subtilis subsp. inaquosorum strain DE111	Bacteria	0.02
1736	91753.8	Cucurbit aphid-borne yellows virus strain Sq/2005/9.2	Viruses	0.02
1737	693582.1	Pseudomonas phage phi-2	Viruses	0.02
1738	1517899.1	Kytococcus sp. CUA-901	Bacteria	0.02
1739	243164	Dehalococcoides mccartyi 195	Bacteria	0.02
1740	1922435.1	Beihai mollusks virus 2 strain WZSLuoI86113	Viruses	0.02
1741	1759399.1	Streptococcus sp. A12 sp. A12	Bacteria	0.02
1742	1118056	Anaerococcus obesiensis ph10	Bacteria	0.02
1743	726.4	Haemophilus haemolyticus strain 27P25	Bacteria	0.02
1744	392504.3	Turnip ringspot virus	Viruses	0.02
1745	392504.4	Turnip ringspot virus isolate M12	Viruses	0.02
1746	36015.1	Pichia kluyveri strain CBS	Eukaryota	0.02
1747	1215915	Lactococcus raffinolactis 4877	Bacteria	0.02
1748	1301100.1	[Clostridium] dakarense strain FF1	Bacteria	0.02
1749	67962.3	Carrot red leaf luteovirus associated RNA clone sigma	Viruses	0.02
1750	936578.1	Streptococcus sp. AS20	Bacteria	0.02
1751	1379.2	Gemella haemolysans strain DNF01167	Bacteria	0.02
1752	79604.1	Denitrobacterium detoxificans strain DSM	Bacteria	0.02
1753	1496722.1	Butyrivibrio sp. AE2005	Bacteria	0.02
1754	1123404	Tissierella praeacuta DSM 18095	Bacteria	0.02
1755	137591.4	Weissella cibaria strain MG1	Bacteria	0.02
1756	137591.5	Weissella cibaria strain AB3b	Bacteria	0.02
1757	1457186	Bifidobacterium longum subsp. longum EK13	Bacteria	0.02
1758	562.2176	Escherichia coli strain MN067	Bacteria	0.02
1759	1613.5	Lactobacillus fermentum strain LfQi6	Bacteria	0.02
1760	1613.6	Lactobacillus fermentum strain RI-508	Bacteria	0.02
1761	1078773	Herbaspirillum rubrisubalbicans M1	Bacteria	0.02
1762	134632.1	American plum line pattern virus	Viruses	0.02
1763	485.83	Neisseria gonorrhoeae strain m07.05	Bacteria	0.02
1764	1502.2	Clostridium perfringens strain 1207_CPER	Bacteria	0.02
1765	1496.601	Clostridioides difficile isolate VL_0330	Bacteria	0.02
1766	1236526	Porphyromonas gingivicanis JCM 15907	Bacteria	0.02
1767	196400.5	Grapevine rupestris stem pitting-associated virus isolate GRSPaV-JF	Viruses	0.02
1768	12162.44	Citrus tristeza virus isolate T68-1	Viruses	0.02
1769	12024.1	Pseudomonas phage PRR1	Viruses	0.02
1770	1423760	Lactobacillus ingluviei DSM 15946	Bacteria	0.02
1771	1235793.1	Lachnospiraceae bacterium COE1	Bacteria	0.02
1772	12430.1	Garlic virus D isolate Mesi	Viruses	0.02
1773	184870.1	Varibaculum cambriense strain DNF00696	Bacteria	0.02
1774	1410662	Lachnospira multipara MC2003	Bacteria	0.02
1775	402626	Ralstonia pickettii 12J	Bacteria	0.02
1776	1095730	Streptococcus constellatus subsp. constellatus SK53	Bacteria	0.02

1777	1445858.1	Enterococcus phage IME-EFm1	Viruses	0.02
1778	1596.8	Lactobacillus gasseri strain AL5	Bacteria	0.02
1779	339420.3	Blackberry chlorotic ringspot virus	Viruses	0.02
1780	1908263.1	Rodentibacter trehalosifermentans strain H1987082031	Bacteria	0.02
1781	762051	Leuconostoc kimchii IMSNU 11154	Bacteria	0.02
1782	260742.1	Streptomyces sp. SS	Bacteria	0.02
1783	1236514	Bacteroides stercorirosoris JCM 17103	Bacteria	0.02
1784	1496.181	Clostridioides difficile isolate VL_0239	Bacteria	0.02
1785	140626.1	Lachnobacterium bovis strain S1b	Bacteria	0.02
1786	1923170.1	Hubei polero-like virus 2 strain QTM26674	Viruses	0.02
1787	1448141	Trueperella pyogenes MS249	Bacteria	0.02
1788	1272.1	Kocuria varians strain G6	Bacteria	0.02
1789	632112.1	Lactobacillus phage Lb338-1	Viruses	0.02
1790	1385385.1	Streptococcus phage TP-778L	Viruses	0.02
1791	4950.2	Torulaspora delbrueckii	Eukaryota	0.02
1792	573061	Clostridium cellulovorans 743B	Bacteria	0.02
1793	1598.19	Lactobacillus reuteri strain I49	Bacteria	0.02
1794	1255.2	Pediococcus pentosaceus strain NKYL15	Bacteria	0.02
1795	562.1794	Escherichia coli strain GN02091	Bacteria	0.02
1796	1739491.1	Streptococcus sp. HMSC067H01	Bacteria	0.02
1797	154339.1	Little cherry virus 2	Viruses	0.02
1798	562.979	Escherichia coli strain upec-127	Bacteria	0.02
1799	637909	Streptococcus gallolyticus UCN34	Bacteria	0.02
1800	65467.5	Cherry green ring mottle virus isolate S10	Viruses	0.02
1801	1321816	Alloscardovia omnicolens F0580	Bacteria	0.02
1802	1229757	Escherichia phage EC6	Viruses	0.02
1803	1120942	Actinomyces georgiae DSM 6843	Bacteria	0.02
1804	1658779.1	Porphyromonadaceae bacterium H1 strain H2	Bacteria	0.02
1805	218923.3	Turnip rosette virus isolate TRoV-2	Viruses	0.02
1806	1304.18	Streptococcus salivarius strain 40-02	Bacteria	0.02
1807	1529923	Norovirus GII/Hu/JP/2010/GII.P7_GII.7/Musashimurayama/TAKAsanKimchi	Viruses	0.02
1808	1280693	Clostridium butyricum AGR2140	Bacteria	0.02
1809	12242.49	Tobacco mosaic virus strain pet-TW	Viruses	0.02
1810	999405	[Clostridium] clostridioforme 90A4	Bacteria	0.02
1811	1889813.1	Anaerolineaceae bacterium oral taxon 439 strain W11661	Bacteria	0.02
1812	1122154	Lactococcus chungangensis CAU 28 = DSM 22330	Bacteria	0.02
1813	55951.12	Grapevine leafroll-associated virus 3 isolate GH24	Viruses	0.02
1814	1229753.1	Escherichia phage phAPEC8	Viruses	0.02
1815	1496.402	Clostridioides difficile strain VRECD0053	Bacteria	0.02
1816	1033733	Anaerococcus senegalensis JC48	Bacteria	0.02
1817	42882.3	Cherry virus A	Viruses	0.02
1818	729.8	Haemophilus parainfluenzae strain 432_HPAR	Bacteria	0.02
1819	729.5	Haemophilus parainfluenzae strain 488_HPAR	Bacteria	0.02

1820	95340	Norwalk-like virus	Viruses	0.02
1821	1739465.1	Enterococcus sp. HMSC076E04	Bacteria	0.02
1822	353496	Lactobacillus delbrueckii subsp. bulgaricus 2038	Bacteria	0.02
1823	1330524.3	Salivirus A isolate BN-5	Viruses	0.02
1824	28131.1	Prevotella intermedia strain ATCC	Bacteria	0.02
1825	980518	Enterobacter mori LMG 25706	Bacteria	0.02
1826	1477000.1	Peptoniphilus sp. DNF00840	Bacteria	0.02
1827	1564903.1	Strawberry polerovirus 1 isolate AB5301	Viruses	0.02
1828	443746.1	Asparagus virus 1 isolate DSMZ	Viruses	0.02
1829	1795648.1	Picornavirales Tottori-HG1	Viruses	0.02
1830	682382.1	HMO Astrovirus A	Viruses	0.02
1831	1423730	Lactobacillus camelliae DSM 22697 = JCM 13995	Bacteria	0.02
1832	1423739	Lactobacillus diolivorans DSM 14421	Bacteria	0.02
1833	1647408.1	Klebsiella phage KLPN1	Viruses	0.02
1834	1316931	Clostridium butyricum DSM 10702	Bacteria	0.02
1835	749551	Selenomonas artemidis F0399	Bacteria	0.02
1836	944557	Prevotella denticola CRIS 18C-A	Bacteria	0.02
1837	1035189	Streptococcus infantis SK970	Bacteria	0.02
1838	12317.5	Tobacco streak virus isolate OK	Viruses	0.02
1839	1318.21	Streptococcus parasanguinis strain DD19	Bacteria	0.02
1840	1200793	Streptococcus salivarius K12	Bacteria	0.02
1841	553218	Campylobacter rectus RM3267	Bacteria	0.02
1842	1932006.2	Chicken associated smacovirus strain RS/BR/2015/4	Viruses	0.02
1843	1206545.1	Klebsiella phage 0507-KN2-1	Viruses	0.02
1844	685899.1	Papaya lethal yellowing virus	Viruses	0.02
1845	1095768	Enterobacter massiliensis JC163	Bacteria	0.02
1846	300717	Sapovirus NongKhai-24/Thailand	Viruses	0.02
1847	5478.1	[Candida] glabrata	Eukaryota	0.02
1848	1597.12	Lactobacillus paracasei strain DSM	Bacteria	0.02
1849	1410625.1	Lachnospiraceae bacterium MD2004	Bacteria	0.02
1850	936577.1	Streptococcus sp. AS14	Bacteria	0.02
1851	12275.1	Tomato black ring virus strain ED)	Viruses	0.02
1852	1073353	Campylobacter showae CC57C	Bacteria	0.02
1853	1444211	Escherichia coli 5-172-05_S4_C1	Bacteria	0.02
1854	150055.1	Streptococcus lutetiensis strain DD06	Bacteria	0.02
1855	1172562	Helicobacter cinaedi PAGU611	Bacteria	0.02
1856	948870.1	Enterobacteria phage phi92	Viruses	0.02
1857	33964.1	Leuconostoc citreum strain 1301_LGAS	Bacteria	0.02
1858	1353.2	Enterococcus gallinarum strain SKF1	Bacteria	0.02
1859	1111137.1	Slackia sp. CM382	Bacteria	0.02
1860	1123309	Streptococcus minor DSM 17118	Bacteria	0.02
1861	1682.1	CECT	Bacteria	0.02
1862	331278.1	Yersinia phage phiR1-37	Viruses	0.02
1863	471285.1	Lettuce yellow mottle virus	Viruses	0.02
1864	1095770	Peptoniphilus timonensis JC401	Bacteria	0.02
1865	1475062.1	Porcine stool-associated circular virus 4 isolate CP2	Viruses	0.02

1866	689781.1	Oribacterium sp. NK2B42	Bacteria	0.02
1867	1354300.1	Peptoniphilus sp. ChDC B134	Bacteria	0.02
1868	1276.2	Kytococcus sedentarius strain 1083_KSED	Bacteria	0.02
1869	1197906	Afipia birgiae 34632	Bacteria	0.02
1870	12041.2	Bean leafroll virus	Viruses	0.02
1871	613026	Helicobacter bilis ATCC 43879	Bacteria	0.02
1872	56879.1	Oat blue dwarf virus isolate OBDV-2r	Viruses	0.02
1873	1339351	Bacteroides vulgatus str. 3775 SR(B) 19	Bacteria	0.02
1874	1073366	Prevotella nigrescens CC14M	Bacteria	0.02
1875	112227.3	Cactus virus X isolate NTU	Viruses	0.02
1876	1354264	Kluyvera georgiana ATCC 51603	Bacteria	0.02
1877	1381124	Lactobacillus fermentum 3872	Bacteria	0.02
1878	575594	Lactobacillus coleohominis 101-4-CHN	Bacteria	0.02
1879	1679.11	Bifidobacterium longum subsp. longum strain LO-10	Bacteria	0.02
1880	525363	Lactobacillus sakei subsp. carnosus DSM 15831	Bacteria	0.02
1881	1599.3	Lactobacillus sakei strain RI-412	Bacteria	0.02
1882	29388.7	Staphylococcus capitis strain 129_SAUR	Bacteria	0.02
1883	1283279	Saccharopolyspora rectivirgula DSM 43747	Bacteria	0.02
1884	28141.1	Cronobacter sakazakii strain HPB5174	Bacteria	0.02
1885	1857568.1	Macellibacteroides sp. HH-ZS	Bacteria	0.02
1886	35703.8	Citrobacter amalonaticus strain FDAARGOS_122	Bacteria	0.02
1887	51288.1	Kluyvera ascorbata strain WCH1410	Bacteria	0.02
1888	1318.6	Streptococcus parasanguinis strain 766_SPAR	Bacteria	0.02
1889	218667.1	Oyster mushroom spherical virus	Viruses	0.02
1890	1522179.1	Asterionellopsis glacialis RNA virus	Viruses	0.02
1891	1339301	Bacteroides fragilis str. 3397 N3	Bacteria	0.02
1892	1581071.1	Granulicatella sp. HMSC30F09	Bacteria	0.02
1893	1685.1	Bifidobacterium breve strain BR-L29	Bacteria	0.02
1894	596322	Streptococcus salivarius SK126	Bacteria	0.02
1895	683173	Astrovirus VA2	Viruses	0.02
1896	52253.1	Candida sojae	Eukaryota	0.02
1897	758863	Sapovirus Hu/GI/Sapporo/MT-2010/1982	Viruses	0.02
1898	39443.2	Carnation Italian ringspot virus isolate CZ	Viruses	0.02
1899	1195163.1	Amazon lily mild mottle virus	Viruses	0.02
1900	12165.3	Chrysanthemum virus B isolate Tamil	Viruses	0.02
1901	315405.1	Streptococcus gallolyticus strain ICDDRB-NRC-S3	Bacteria	0.02
1902	34073.1	Variovorax paradoxus strain H112	Bacteria	0.02
1903	1785087.1	Candidatus Protochlamydia sp. W-9 sp. W-9	Bacteria	0.02
1904	1122988	Prevotella nanceiensis DSM 19126 = JCM 15639	Bacteria	0.02
1905	1303.18	Streptococcus oralis strain DD24	Bacteria	0.02
1906	641149	Neisseria sp. oral taxon 014 str. F0314	Bacteria	0.02
1907	29363.3	Clostridium paraputrificum strain 2789STDY5834857	Bacteria	0.02
1908	1715012.1	Enterococcus sp. HMSC072H05	Bacteria	0.02
1909	1852625.1	Klebsiella phage vB_KpnM_KpV477	Viruses	0.02
1910	1081640	Sphingomonas elodea ATCC 31461	Bacteria	0.02

1911	1127128	Leuconostoc citreum LBAE C11	Bacteria	0.02
1912	28348.2	Sweet clover necrotic mosaic virus	Viruses	0.02
1913	1665651	Norovirus GII/Hu/NL/2014/GII.2/Groningen	Viruses	0.02
1914	1211388.1	Apple green crinkle associated virus isolate Aurora-1	Viruses	0.02
1915	50948.1	Enterobacteria phage RB49	Viruses	0.02
1916	1261065	Gardnerella vaginalis JCP8070	Bacteria	0.02
1917	727.77	Haemophilus influenzae strain 159_HINF	Bacteria	0.02
1918	1354263	Hafnia paralvei ATCC 29927	Bacteria	0.02
1919	1913024.1	White clover mottle virus	Viruses	0.02
1920	227507.1	Strawberry pallidosis-associated virus	Viruses	0.02
1921	1193128	Parascardovia denticolens IPLA 20019	Bacteria	0.02
1922	1280675.1	Bifidobacterium sp. AGR2158	Bacteria	0.02
1923	1739450.1	Actinomyces sp. HMSC062G12	Bacteria	0.02
1924	879306	Anaerococcus hydrogenalis ACS-025-V-Sch4	Bacteria	0.02
1925	997352	Prevotella nigrescens ATCC 33563	Bacteria	0.02
1926	1630.1	Kandleria vitulina strain WCC7	Bacteria	0.02
1927	1906334.1	Corynebacterium sp. NML140438	Bacteria	0.02
1928	596153	Alicycliphilus denitrificans BC	Bacteria	0.02
1929	46170.38	Staphylococcus aureus subsp. aureus strain SA-120	Bacteria	0.02
1930	1610829	Klebsiella phage 1513	Viruses	0.02
1931	888064	Enterococcus italicus DSM 15952	Bacteria	0.02
1932	1923554.1	Wenzhou bivalvia virus 2 strain beimix75763	Viruses	0.02
1933	585.5	Proteus vulgaris strain ATCC	Bacteria	0.02
1934	199.3	Campylobacter concisus strain ATCC	Bacteria	0.02
1935	469607	Fusobacterium nucleatum subsp. animalis 4_8	Bacteria	0.02
1936	1416758	Morganella morganii H1r	Bacteria	0.02
1937	1547495.1	Salivirus FHB	Viruses	0.02
1938	1204476	Fusobacterium nucleatum CTI-3	Bacteria	0.02
1939	702439	Prevotella nigrescens F0103	Bacteria	0.02
1940	1868658.1	Human astrovirus	Viruses	0.02
1941	1327956	Escherichia phage JES2013	Viruses	0.02
1942	12055.1	Tobacco necrosis virus A	Viruses	0.02
1943	868164	Escherichia coli DEC6E	Bacteria	0.02
1944	570949.1	Carrot mottle mimic virus satellite RNA	Viruses	0.02
1945	1408324.1	Lachnospiraceae bacterium MC2017	Bacteria	0.02
1946	582.3	Morganella morganii strain L3	Bacteria	0.02
1947	28038.4	Lactobacillus curvatus strain RI-406	Bacteria	0.02
1948	1339291	Bacteroides fragilis str. S23 R14	Bacteria	0.02
1949	10590	Human papillomavirus type 42	Viruses	0.02
1950	351495.1	Raphanus sativus cryptic virus 2	Viruses	0.02
1951	12433.3	Garlic virus A isolate SW3.1A	Viruses	0.02
1952	12433.4	Garlic virus A isolate GarVA-SP	Viruses	0.02
1953	12049.1	Soybean dwarf virus	Viruses	0.02
1954	1590.151	Lactobacillus plantarum strain MF1298	Bacteria	0.02
1955	12431.2	Garlic virus C isolate SW3.3A	Viruses	0.02
1956	399183	Escherichia phage V5	Viruses	0.02

1957	1698360.1	Klebsiella phage JD18	Viruses	0.02
1958	1304.6	Streptococcus salivarius strain 1003_SOLI	Bacteria	0.02
1959	1852368.1	Prevotellaceae bacterium Marseille-P2826	Bacteria	0.02
1960	315405.11	Streptococcus gallolyticus strain DD02	Bacteria	0.02
1961	722911	Bifidobacterium longum subsp. longum F8	Bacteria	0.02
1962	1563222.1	Citrobacter pasteurii strain CIP	Bacteria	0.02
1963	217686.1	Little cherry virus 1	Viruses	0.02
1964	1339347	Bacteroides ovatus str. 3725 D9 iii	Bacteria	0.02
1965	28347.11	Apple stem grooving virus isolate ASGV-CHN	Viruses	0.02
1966	499174	Clostridioides difficile QCD-23m63	Bacteria	0.02
1967	1537165.1	Porcine stool-associated circular virus 6 isolate XP1	Viruses	0.02
1968	1163671.1	Clostridium sp. 12(A)	Bacteria	0.02
1969	156976.1	Corynebacterium riegelii strain PUDD_83A45	Bacteria	0.02
1970	1737425.1	Corynebacterium provencense strain SN15	Bacteria	0.02
1971	1032457.1	Passion fruit mosaic virus	Viruses	0.02
1972	53655.1	Pichia fermentans	Eukaryota	0.02
1973	1654357.1	La Jolla virus	Viruses	0.02
1974	1002805	Helicobacter bizzozeronii CCUG 35545	Bacteria	0.02
1975	1127129	Leuconostoc citreum LBAE E16	Bacteria	0.02
1976	1654356.1	Thika virus	Viruses	0.02
1977	562.496	Escherichia coli strain 592_ECOL	Bacteria	0.02
1978	1581074.1	Granulicatella sp. HMSC31F03	Bacteria	0.02
1979	1131317	Escherichia phage FV3	Viruses	0.02
1980	1665644	Norovirus GI/Hu/NL/2011/GI.4/Groningen	Viruses	0.02
1981	1183241.1	Persimmon cryptic virus isolate SSPI	Viruses	0.02
1982	359987.1	Rhizosolenia setigera RNA virus 01	Viruses	0.02
1983	59241.1	Streptococcus phage Dp-1	Viruses	0.02
1984	1211817	Clostridium ihumii AP5	Bacteria	0.02
1985	1923116.1	Hubei picorna-like virus 36 strain SCM50248	Viruses	0.02
1986	1121269	Campylobacter upsaliensis DSM 5365	Bacteria	0.02
1987	1123489	Veillonella magna DSM 19857	Bacteria	0.02
1988	562.1822	Escherichia coli strain SF-491	Bacteria	0.02
1989	1351.11	Enterococcus faecalis	Bacteria	0.02
1990	92395.1	Black queen cell virus strain PP	Viruses	0.01
1991	196400.16	Grapevine rupestris stem pitting-associated virus isolate GRSPaV-MG	Viruses	0.01
1992	1218493.1	Lactobacillus kullabergensis strain Biut2	Bacteria	0.01
1993	1429888	Leuconostoc mesenteroides P45	Bacteria	0.01
1994	1284702	Mageeibacillus indolicus 0009-5	Bacteria	0.01
1995	64003.1	Grapevine leafroll-associated virus 2 isolate GLRaV-2-SG	Viruses	0.01
1996	1229204.1	alpha proteobacterium L41A	Bacteria	0.01
1997	1093141	Nannochloropsis gaditana CCMP526	Eukaryota	0.01
1998	336306.3	Enterobacter cloacae subsp. cloacae strain GN02616	Bacteria	0.01
1999	11987.3	Melon necrotic spot virus isolate Malfa5	Viruses	0.01
2000	216816.19	Bifidobacterium longum strain BG7	Bacteria	0.01

2001	1505227.1	Aeromonas phage pAh6-C	Viruses	0.01
2002	329.1	Ralstonia pickettii strain H2Cu5	Bacteria	0.01
2003	349519	Leuconostoc citreum KM20	Bacteria	0.01
2004	1551.1	Clostridium aurantibutyricum strain DSM	Bacteria	0.01
2005	169292.4	Corynebacterium aurimucosum strain 620_CAUR	Bacteria	0.01
2006	1566990.1	Streptococcus phage SpSL1	Viruses	0.01
2007	1502.38	Clostridium perfringens strain FORC_025	Bacteria	0.01
2008	641487.1	Lactococcus phage P087	Viruses	0.01
2009	1235801	Lactobacillus murinus ASF361	Bacteria	0.01
2010	649760	Prevotella oris F0302	Bacteria	0.01
2011	577.2	Raoultella terrigena strain NZ133	Bacteria	0.01
2012	1297581	Anoxybacillus flavithermus AK1	Bacteria	0.01
2013	562.1581	Escherichia coli strain GN02461	Bacteria	0.01
2014	1304.15	Streptococcus salivarius strain 84-12	Bacteria	0.01
2015	546.1	Citrobacter freundii strain CF7_ST91	Bacteria	0.01
2016	620833	Fusobacterium periodonticum D10	Bacteria	0.01
2017	1302.16	Streptococcus gordonii strain 1116_SGOR	Bacteria	0.01
2018	762550	Leuconostoc gelidum subsp. gasicomitatum LMG 18811	Bacteria	0.01
2019	633135.1	Streptococcus phage Abc2	Viruses	0.01
2020	448384.1	Enterobacteria phage Phi1	Viruses	0.01
2021	1868652.2	High Plains wheat mosaic virus	Viruses	0.01
2022	1868652.1	High Plains wheat mosaic virus isolate Nebraska	Viruses	0.01
2023	1284663	Lactobacillus plantarum ZJ316	Bacteria	0.01
2024	1365969	Bifidobacterium breve MCC 1605	Bacteria	0.01
2025	1787.2	Mycobacterium szulgai strain ACS1160	Bacteria	0.01
2026	1898961.1	Kluyvera intestini strain GT-16	Bacteria	0.01
2027	12317.4	Tobacco streak virus isolate dp	Viruses	0.01
2028	12188.1	White clover mosaic virus	Viruses	0.01
2029	1425364.2	Carrot torradovirus 1 isolate CTV-1_RNA1_H6	Viruses	0.01
2030	392504.2	Turnip ringspot virus isolate CV-3	Viruses	0.01
2031	1496.635	Clostridioides difficile isolate VL_0468	Bacteria	0.01
2032	1231072	Clostridium tetani 12124569	Bacteria	0.01
2033	501571.1	Butyricicoccus	Bacteria	0.01
2034	877422	Butyrivibrio hungatei NK4A153	Bacteria	0.01
2035	1089447	Aggregatibacter actinomycetemcomitans RhAA1	Bacteria	0.01
2036	113574.1	Hyphomicrobium sp. GJ21 sp. GJ21	Bacteria	0.01
2037	537970	Helicobacter canadensis MIT 98-5491	Bacteria	0.01
2038	936554.1	Campylobacter sp. FOBRC14	Bacteria	0.01
2039	1496.457	Clostridioides difficile strain VRECD0002	Bacteria	0.01
2040	1437607	Bifidobacterium saguini DSM 23967	Bacteria	0.01
2041	932006	Bacillus subtilis subsp. spizizenii RFWG1A3	Bacteria	0.01
2042	1913031	Papaya ringspot virus-W	Viruses	0.01
2043	1739479.1	Actinomyces sp. HMSC065F12	Bacteria	0.01
2044	1437600	Bifidobacterium pullorum DSM 20433	Bacteria	0.01
2045	185902	Human rhinovirus A31	Viruses	0.01
2046	185905	Human rhinovirus A34	Viruses	0.01
2047	817.25	Bacteroides fragilis strain O:21	Bacteria	0.01

2048	1685.4	Bifidobacterium breve strain BR-I29	Bacteria	0.01
2049	1354.1	Enterococcus hirae strain 19m	Bacteria	0.01
2050	1681.3	2789STDY5608877	Bacteria	0.01
2051	303541.1	Lactobacillus apis strain R-53131	Bacteria	0.01
2052	562.78	Escherichia coli strain VL127	Bacteria	0.01
2053	1051661	Lactobacillus casei UW4	Bacteria	0.01
2054	562.77	Escherichia coli strain AF7658-2	Bacteria	0.01
2055	195.1	Campylobacter coli	Bacteria	0.01
2056	1923308.1	Hubei toti-like virus 2 strain arthropodmix13450	Viruses	0.01
2057	32629.1	Indian peanut clump virus	Viruses	0.01
2058	1123243	Schwartzia succinivorans DSM 10502	Bacteria	0.01
2059	135750	Tomato bushy stunt virus satellite RNA B10	Viruses	0.01
2060	1568973.1	Botrytis cinerea RNA virus 1 strain BerBc-1	Viruses	0.01
2061	1302.23	Streptococcus gordonii strain G9B	Bacteria	0.01
2062	1439319.1	Citrobacter sp. MGH 55	Bacteria	0.01
2063	137591.6	Weissella cibaria strain CH2	Bacteria	0.01
2064	1222338.1	Enterobacteria phage GEC-3S	Viruses	0.01
2065	1293592	Lactobacillus curvatus JCM 1096 = DSM 20019	Bacteria	0.01
2066	1496.325	Clostridioides difficile strain VRECD0035	Bacteria	0.01
2067	1244083	Campylobacter showae CSUNSWCD	Bacteria	0.01
2068	1613.3	Lactobacillus fermentum strain DSM	Bacteria	0.01
2069	1423813	Lactobacillus vaccinostercus DSM 20634	Bacteria	0.01
2070	910996	Hafnia alvei ATCC 13337	Bacteria	0.01
2071	1588750.1	Clostridiales bacterium KA00134	Bacteria	0.01
2072	1219360	Erwinia persicina NBRC 102418	Bacteria	0.01
2073	1605.1	Lactobacillus animalis strain P38	Bacteria	0.01
2074	1081904	Prevotella pleuritidis F0068	Bacteria	0.01
2075	291175	Sapovirus Hu/Dresden/pJG-Sap01/DE	Viruses	0.01
2076	1283.11	Staphylococcus haemolyticus strain 1292_SHAE	Bacteria	0.01
2077	1232440	Hungatella hathewayi VE202-04	Bacteria	0.01
2078	562.134	Escherichia coli isolate YS	Bacteria	0.01
2079	1504.1	Clostridium septicum strain P1044	Bacteria	0.01
2080	267135.9	Porphyrobacter donghaensis strain CCH1-A1	Bacteria	0.01
2081	1450518	Sphingobium lucknowense F2	Bacteria	0.01
2082	12196.45	Bean common mosaic virus isolate 1755a	Viruses	0.01
2083	1702221.1	Faecalibaculum rodentium strain NYU-BL-K8	Bacteria	0.01
2084	1193182	Tetrasphaera australiensis Ben110	Bacteria	0.01
2085	328061.1	Radish mosaic virus isolate DH-1	Viruses	0.01
2086	439016.1	Marine RNA virus JP-B	Viruses	0.01
2087	151043.1	Tulare apple mosaic virus	Viruses	0.01
2088	28037.3	Streptococcus mitis strain SK629	Bacteria	0.01
2089	1414721.1	Clostridium jeddahense strain JCD	Bacteria	0.01
2090	1715019.1	Enterococcus sp. HMSC064A12	Bacteria	0.01
2091	1242969	Campylobacter concisus ATCC 51562	Bacteria	0.01
2092	92444.3	Acute bee paralysis virus isolate Hungary	Viruses	0.01
2093	1739351.1	Corynebacterium sp. HMSC074C03	Bacteria	0.01
2094	267135.1	Porphyrobacter donghaensis strain CCH7-A10	Bacteria	0.01
2095	1120992	Anaeromusa acidaminophila DSM 3853	Bacteria	0.01

2096	1223528	Microbacterium oleivorans NBRC 103075	Bacteria	0.01
2097	747.4	Pasteurella multocida strain 306_PMUL	Bacteria	0.01
2098	1715007.1	Rothia sp. HMSC071B01	Bacteria	0.01
2099	1281072	Escherichia coli HVH 139 (4-3192644)	Bacteria	0.01
2100	1739310.1	Turicella sp. HMSC076G08	Bacteria	0.01
2101	1077464.1	Streptococcus oralis subsp. tigurinus strain DGIIBVI	Bacteria	0.01
2102	12056.2	Tobacco necrosis virus D isolate TNV-DP	Viruses	0.01
2103	1220025.4	Pokeweed mosaic virus isolate PkMV-NJ	Viruses	0.01
2104	1235640.1	Enterobacteria phage M	Viruses	0.01
2105	857154	Streptococcus mutans 1ID3	Bacteria	0.01
2106	944560	Actinomyces sp. oral taxon 175 str. F0384	Bacteria	0.01
2107	1714265.1	Klebsiella sp. KGM-IMP216	Bacteria	0.01
2108	12235.13	Cucumber green mottle mosaic virus isolate Ec	Viruses	0.01
2109	586220	Leuconostoc mesenteroides subsp. cremoris ATCC 19254	Bacteria	0.01
2110	44135	Human rhinovirus A65	Viruses	0.01
2111	1519399.1	Sewage-associated gemycircularvirus 4 isolate BS3913	Viruses	0.01
2112	187978.1	Peru tomato mosaic virus	Viruses	0.01
2113	1923604.1	Wenzhou picorna-like virus 2 strain beimix73672	Viruses	0.01
2114	586419.2	Human cosavirus A strain CMH-N199-11	Viruses	0.01
2115	45972.1	Staphylococcus pasteuri strain 915_SPAS	Bacteria	0.01
2116	134533.1	Acinetobacter parvus strain CM11	Bacteria	0.01
2117	1318.1	Streptococcus parasanguinis strain 349_SPAR	Bacteria	0.01
2118	1318.18	Streptococcus parasanguinis strain C1A	Bacteria	0.01
2119	76860.1	Streptococcus constellatus strain 317_SINT	Bacteria	0.01
2120	5755.1	Acanthamoeba castellanii	Eukaryota	0.01
2121	1410666	Prevotella brevis P6B11	Bacteria	0.01
2122	1610835	Klebsiella phage vB_KpnP_SU552A	Viruses	0.01
2123	1679.15	Bifidobacterium longum subsp. longum strain NCIMB8809	Bacteria	0.01
2124	569.3	Hafnia alvei strain GB001	Bacteria	0.01
2125	1321820	Gemella bergeriae ATCC 700627	Bacteria	0.01
2126	630199.1	Grapevine Syrah virus 1 isolate VF-BR	Viruses	0.01
2127	1433126.1	Mucinivorans hirudinis	Bacteria	0.01
2128	11986.1	Carnation mottle virus	Viruses	0.01
2129	1095736	Streptococcus mitis SK575	Bacteria	0.01
2130	1095737	Streptococcus mitis SK579	Bacteria	0.01
2131	1321950	Clostridium butyricum CWBI1009	Bacteria	0.01
2132	12275.3	Tomato black ring virus	Viruses	0.01
2133	1598.13	Lactobacillus reuteri strain MD	Bacteria	0.01
2134	35350.11	Apple stem pitting virus strain N	Viruses	0.01
2135	1768792.1	Erythrobacter sp. CCH5-A1	Bacteria	0.01
2136	1208313	Piscicoccus intestinalis NBRC 104926	Bacteria	0.01
2137	1270.3	Micrococcus luteus strain O'kane	Bacteria	0.01
2138	837.8	Porphyromonas gingivalis strain 84_3 isolate 84_3	Bacteria	0.01
2139	908337	Eremococcus coleocola ACS-139-V-Col8	Bacteria	0.01

2140	1122129	Jeotgalicoccus psychrophilus DSM 19085	Bacteria	0.01
2141	999415	Eggerthia catenaformis OT 569 = DSM 20559	Bacteria	0.01
2142	4896.1	Schizosaccharomyces pombe	Eukaryota	0.01
2143	140626.2	Lachnobacterium bovis strain AE2004	Bacteria	0.01
2144	1739400.1	Corynebacterium sp. HMSC069E04	Bacteria	0.01
2145	1258574	Streptococcus gallolyticus subsp. gallolyticus DSM 16831	Bacteria	0.01
2146	1715164.1	Streptococcus sp. HMSC074F05	Bacteria	0.01
2147	255248.1	Leuconostoc garlicum strain KFRI01	Bacteria	0.01
2148	1444120	Escherichia coli 2-156-04_S3_C1	Bacteria	0.01
2149	1129192.1	Bacillus phage BCP8-2	Viruses	0.01
2150	1496.477	Clostridioides difficile strain VRECD0180	Bacteria	0.01
2151	1496.476	Clostridioides difficile strain VRECD0025	Bacteria	0.01
2152	1423759	Lactobacillus hordei DSM 19519	Bacteria	0.01
2153	1423753	Lactobacillus hammesii DSM 16381	Bacteria	0.01
2154	548.55	Klebsiella aerogenes strain 35003	Bacteria	0.01
2155	1353980	Shigella dysenteriae SD1D	Bacteria	0.01
2156	1219585.1	Arcanobacterium sp. S3PF19	Bacteria	0.01
2157	736.1	Haemophilus paraphrohaemolyticus strain CCUG	Bacteria	0.01
2158	857143	Streptococcus mutans 11VS1	Bacteria	0.01
2159	398513	Bifidobacterium bifidum NCIMB 41171	Bacteria	0.01
2160	1542743.1	Caribou feces-associated gemycircularvirus	Viruses	0.01
2161	971.3	Selenomonas ruminantium strain S137	Bacteria	0.01
2162	1255.5	Pediococcus pentosaceus strain LP28	Bacteria	0.01
2163	1255.4	Pediococcus pentosaceus	Bacteria	0.01
2164	195.518	Campylobacter coli isolate M2D2	Bacteria	0.01
2165	28141.47	Cronobacter sakazakii strain MOD1-Md33g	Bacteria	0.01
2166	12232.13	Zucchini yellow mosaic virus	Viruses	0.01
2167	1529.1	Clostridium cadaveris strain NLAE-zl-G419	Bacteria	0.01
2168	1167632	Staphylococcus vitulinus F1028	Bacteria	0.01
2169	563037.1	Streptococcus sp. M143	Bacteria	0.01
2170	438780.1	Lactobacillus phage phiPYB5	Viruses	0.01
2171	1210046	Janibacter hoylei PVAS-1	Bacteria	0.01
2172	195103	Clostridium perfringens ATCC 13124	Bacteria	0.01
2173	1064592	Naumovozyma castellii CBS 4309	Eukaryota	0.01
2174	1652048	Sapovirus Hu/GI.1/Seoul/ROK62/2013/KOR	Viruses	0.01
2175	1410674	Sharpea azabuensis DSM 18934	Bacteria	0.01
2176	1410672	Ruminococcus flavefaciens ND2009	Bacteria	0.01
2177	1541211.1	Cripavirus NB-1/2011/HUN	Viruses	0.01
2178	29363.2	Clostridium paraputrificum strain 2789STDY5834955	Bacteria	0.01
2179	562.1739	Escherichia coli strain M8	Bacteria	0.01
2180	1269760	Lactobacillus delbrueckii subsp. lactis CRL581	Bacteria	0.01
2181	1051676.1	Erwinia phage vB_EamM-Y2	Viruses	0.01
2182	550.13	Enterobacter cloacae strain MNCRE12	Bacteria	0.01
2183	1795832.1	Eikenella sp. NML130454	Bacteria	0.01
2184	656024.1	Frankia symbiont of Datisca glomerata	Bacteria	0.01
2185	1293.1	Staphylococcus gallinarum strain DSM	Bacteria	0.01

2186	1647391.1	Streptococcus phage APCM01	Viruses	0.01
2187	1280697	Butyrivibrio fibrisolvens AB2020	Bacteria	0.01
2188	1280692	Clostridium cadaveris AGR2141	Bacteria	0.01
2189	48296.22	Acinetobacter pittii strain ABBL074	Bacteria	0.01
2190	1236504	Prevotella histicola JCM 15637 = DNF00424	Bacteria	0.01
2191	866773	Finegoldia magna BVS033A4	Bacteria	0.01
2192	1122157	Laribacter hongkongensis DSM 14985	Bacteria	0.01
2193	1335616.1	Lactobacillus wasatchensis strain WDC04	Bacteria	0.01
2194	35288.5	Grapevine virus A isolate GTR1-1	Viruses	0.01
2195	1408894.1	Red clover cryptic virus 1 isolate IPP_Nemaro	Viruses	0.01
2196	158787.1	Bifidobacterium scardovii strain 981_BLON	Bacteria	0.01
2197	1055192.1	Comamonas sp. B-9	Bacteria	0.01
2198	571.8	Klebsiella oxytoca strain 2880STDY5682571	Bacteria	0.01
2199	419015.3	Alloscardovia omnicolens strain 476_GVAG	Bacteria	0.01
2200	419015.4	Alloscardovia omnicolens strain 350_GVAG	Bacteria	0.01
2201	1622070.1	Paenibacillus sp. GM2 sp. GM2	Bacteria	0.01
2202	1496.311	Clostridioides difficile strain CD10	Bacteria	0.01
2203	28125.2	Prevotella bivia strain GED7760C	Bacteria	0.01
2204	633147	Olsenella uli DSM 7084	Bacteria	0.01
2205	563038.1	Streptococcus sp. M334	Bacteria	0.01
2206	1415765	Streptococcus mitis 21/39	Bacteria	0.01
2207	905067	Streptococcus parasanguinis F0405	Bacteria	0.01
2208	1581143.1	Arthrobacter sp. HMSC08H08	Bacteria	0.01
2209	1692238.1	Enterobacter sp. FY-07 sp. FY-07	Bacteria	0.01
2210	1692238.2	Enterobacter sp. FY-07	Bacteria	0.01
2211	185932	Human rhinovirus A77	Viruses	0.01
2212	1293441.1	Lysinibacillus contaminans strain DSM	Bacteria	0.01
2213	947969	Cellulomonas carbonis T26	Bacteria	0.01
2214	1294274	Streptococcus equinus JB1	Bacteria	0.01
2215	1080365	Pediococcus acidilactici MA18/5M	Bacteria	0.01
2216	1817674.1	Geobacillus sp. 8	Bacteria	0.01
2217	328430.1	Chickpea chlorotic stunt virus	Viruses	0.01
2218	1384081.1	Veillonella sp. DNF00869	Bacteria	0.01
2219	1599.7	Lactobacillus sakei strain RI-409	Bacteria	0.01
2220	307486.1	Tepidimonas taiwanensis strain MB2	Bacteria	0.01
2221	42004.3	Leek yellow stripe virus isolate AG1	Viruses	0.01
2222	1446490	Escherichia phage FFH2	Viruses	0.01
2223	1912598.1	Cherry associated luteovirus	Viruses	0.01
2224	1871025.1	Ndongobacter massiliensis strain Marseille-P3170T sp. Marseille-P3170	Bacteria	0.01
2225	222805.6	Mycobacterium chimaera strain AH16	Bacteria	0.01
2226	571.33	Klebsiella oxytoca strain k142b	Bacteria	0.01
2227	1748.2	F3E8	Bacteria	0.01
2228	675631	Desulfurococcus mobilis DSM 2161	Archaea	0.01
2229	871203.2	Caballeronia zhejiangensis strain CEIB	Bacteria	0.01
2230	941824	Thermobrachium celere DSM 8682	Bacteria	0.01
2231	1280676.1	Butyrivibrio sp. WCD3002	Bacteria	0.01
2232	61647.4	Pluralibacter gergoviae strain DL84A27	Bacteria	0.01

2233	209529.2	Aphid lethal paralysis virus isolate ALPV-An	Viruses	0.01
2234	1873985.1	Salmonella phage IME207	Viruses	0.01
2235	1739536.1	Corynebacterium sp. HMSC073D01	Bacteria	0.01
2236	1402966	Mycobacterium avium subsp. hominissuis 100	Bacteria	0.01
2237	936562.1	Fusobacterium sp. CM21	Bacteria	0.01
2238	1686381.1	Citrobacter sp. MGH106	Bacteria	0.01
2239	28115.1	Porphyromonas macacae strain COT-192	Bacteria	0.01
2240	381764	Fervidobacterium nodosum Rt17-B1	Bacteria	0.01
2241	936572.1	Selenomonas sp. FOBRC6	Bacteria	0.01
2242	1462608.1	Pseudomonas phage KPP25	Viruses	0.01
2243	47770.11	Lactobacillus crispatus strain C037	Bacteria	0.01
2244	47770.12	Lactobacillus crispatus strain PSS7772C	Bacteria	0.01
2245	1089548	Thermicanus aegyptius DSM 12793	Bacteria	0.01
2246	544580.12	Actinomyces oris strain G53E	Bacteria	0.01
2247	544580.16	Actinomyces oris strain F4D1	Bacteria	0.01
2248	1768771.1	Streptococcus sp. CCH8-G7	Bacteria	0.01
2249	498216	Lactobacillus casei str. Zhang	Bacteria	0.01
2250	1581089.1	Corynebacterium sp. HMSC11E11	Bacteria	0.01
2251	1169321.1	Escherichia sp. KTE114	Bacteria	0.01
2252	1739496.1	Prevotella sp. HMSC069G02	Bacteria	0.01
2253	12268.1	Carnation ringspot virus	Viruses	0.01
2254	2162.2	Methanobacterium formicicum	Archaea	0.01
2255	1545701.1	Lactobacillus sp. wkB10	Bacteria	0.01
2256	1423738	Lactobacillus dextrinicus DSM 20335	Bacteria	0.01
2257	1408226	Vagococcus lutrae LBD1	Bacteria	0.01
2258	12280.2	Tomato ringspot virus	Viruses	0.01
2259	78541.1	Streptococcus phage Sfi11	Viruses	0.01
2260	1675603.1	Citrobacter phage Michonne	Viruses	0.01
2261	29484.2	Yersinia frederiksenii strain FCF208	Bacteria	0.01
2262	1340495	Lactobacillus reuteri I5007	Bacteria	0.01
2263	1204529	Salmonella phage SSE121	Viruses	0.01
2264	1216979	Aerococcus urinae NBRC 15544 = CCUG 36881	Bacteria	0.01
2265	888055	Leptotrichia wadei F0279	Bacteria	0.01
2266	1029718	Candidatus Arthromitus sp. SFB-mouse-Japan	Bacteria	0.01
2267	1481465.1	Tomato necrotic dwarf virus isolate R	Viruses	0.01
2268	1318.22	Streptococcus parasanguinis strain MGH413	Bacteria	0.01
2269	553174	Prevotella melaninogenica ATCC 25845	Bacteria	0.01
2270	273525	Pear black necrotic leaf spot virus	Viruses	0.01
2271	562.703	Escherichia coli strain CVM	Bacteria	0.01
2272	1932006.4	Chicken associated smacovirus strain RS/BR/2015/2	Viruses	0.01
2273	1134687.28	Klebsiella michiganensis strain 97_38	Bacteria	0.01
2274	1512.2	[Clostridium] symbiosum	Bacteria	0.01
2275	1280.912	Staphylococcus aureus strain 01-19	Bacteria	0.01
2276	1514105.1	Erysipelothrix larvae sp. LV19	Bacteria	0.01
2277	1138898	Enterococcus faecium EnGen0001	Bacteria	0.01
2278	169292.2	1237_CAUR	Bacteria	0.01

2279	1529909	Norovirus GI/Hu/JP/2007/GI.P3_GI.3/Shimizu/KK2866	Viruses	0.01
2280	1302863	Streptococcus cristatus AS 1.3089	Bacteria	0.01
2281	1922438.1	Beihai narna-like virus 11 strain BWBFG39775	Viruses	0.01
2282	1590.44	Lactobacillus plantarum strain FBR4	Bacteria	0.01
2283	216816.2	Bifidobacterium longum strain 379	Bacteria	0.01
2284	300715	Sapovirus Chanthaburi-74/Thailand	Viruses	0.01
2285	228582.1	Cereal yellow dwarf virus-RPS	Viruses	0.01
2286	1122172	Leptotrichia shahii DSM 19757	Bacteria	0.01
2287	33936.1	Aeribacillus pallidus strain 8m3	Bacteria	0.01
2288	646010.2	Suakwa aphid-borne yellows virus isolate DL76	Viruses	0.01
2289	1814960.1	Streptococcus virus 9874	Viruses	0.01
2290	713030.1	Selenomonas sp. oral taxon 136 strain F0591	Bacteria	0.01
2291	2702.1	Gardnerella vaginalis strain GV37	Bacteria	0.01
2292	1423720	Lactobacillus alimentarius DSM 20249	Bacteria	0.01
2293	936594.1	Lachnoanaerobaculum sp. ICM7	Bacteria	0.01
2294	12275.2	Tomato black ring virus isolate TBRV-Mirs	Viruses	0.01
2295	550.373	Enterobacter cloacae strain WCHECl-C4	Bacteria	0.01
2296	562973	Actinomyces viscosus C505	Bacteria	0.01
2297	257464.2	Potato black ringspot virus isolate PRI-Ec	Viruses	0.01
2298	1673719.1	Anaerococcus sp. SB3	Bacteria	0.01
2299	1825924.1	Barley virus G isolate Gimje	Viruses	0.01
2300	33966.1	Leuconostoc mesenteroides subsp. dextranicum strain LbE15	Bacteria	0.01
2301	1158602	Enterococcus raffinosus ATCC 49464	Bacteria	0.01
2302	1358009	Xanthomonas campestris pv. campestris str. CFBP 5817	Bacteria	0.01
2303	1246.1	Leuconostoc lactis strain WIKIM21	Bacteria	0.01
2304	232846.1	IITR89	Bacteria	0.01
2305	1121307	Clostridium cylindrosporum DSM 605	Bacteria	0.01
2306	46256.1	Weissella hellenica strain R-53116	Bacteria	0.01
2307	1770210.1	Micrococcus sp. CH7	Bacteria	0.01
2308	1496.11	Clostridioides difficile strain VRECD0055	Bacteria	0.01
2309	712538.1	Selenomonas sp. oral taxon 478	Bacteria	0.01
2310	1353.4	Enterococcus gallinarum	Bacteria	0.01
2311	1410654	Corynebacterium vitaeruminis Ga6A13	Bacteria	0.01
2312	150285.1	Garlic virus E	Viruses	0.01
2313	1195085.1	Cronobacter phage CR5	Viruses	0.01
2314	156978.1	Corynebacterium imitans strain DSM	Bacteria	0.01
2315	44008.7	Enterococcus cecorum strain BB-66	Bacteria	0.01
2316	44008.6	Enterococcus cecorum strain CB-32	Bacteria	0.01
2317	44008.3	Enterococcus cecorum strain CL-1	Bacteria	0.01
2318	546.23	Citrobacter freundii strain 804_CKOS	Bacteria	0.01
2319	1682.3	Bifidobacterium longum subsp. infantis strain IN-F29	Bacteria	0.01
2320	1169330	Escherichia coli KTE10	Bacteria	0.01
2321	928328	Lactobacillus iners UPII 60-B	Bacteria	0.01
2322	44562.1	Pothos latent virus	Viruses	0.01

2323	1379702.1	Methanobacterium sp. MB1	Archaea	0.01
2324	1540094.1	Citrobacter phage Moogle	Viruses	0.01
2325	129395.2	Botrytis virus F	Viruses	0.01
2326	1423726	Lactobacillus bifermentans DSM 20003	Bacteria	0.01
2327	1304.28	Streptococcus salivarius strain UC3162	Bacteria	0.01
2328	1256988	Providencia alcalifaciens 205/92	Bacteria	0.01
2329	550.31	Enterobacter cloacae strain CB2	Bacteria	0.01
2330	31631	Human coronavirus OC43	Viruses	0.01
2331	1567453.1	Lactobacillus phage LfeSau	Viruses	0.01
2332	888808	Streptococcus sanguinis SK49	Bacteria	0.01
2333	582.15	Morganella morganii strain FDAARGOS_172	Bacteria	0.01
2334	463676.6	Rhinovirus C isolate 1570-MY-10	Viruses	0.01
2335	388452.1	Lactococcus phage KSY1	Viruses	0.01
2336	2094.1	Mycoplasma arginini	Bacteria	0.01
2337	729.2	Haemophilus parainfluenzae strain ATCC	Bacteria	0.01
2338	56879.2	Oat blue dwarf virus	Viruses	0.01
2339	1264.2	Ruminococcus albus strain KH2T6	Bacteria	0.01
2340	131082.2	Beet chlorosis virus isolate BChV-CR	Viruses	0.01
2341	1639.45	Listeria monocytogenes strain CFSAN026586	Bacteria	0.01
2342	1681197.1	Arthrobacter sp. RIT-PI-e	Bacteria	0.01
2343	1736702.1	Enterobacter sp. K66-74	Bacteria	0.01
2344	28901.16	Salmonella enterica strain NGUA31	Bacteria	0.01
2345	796937.3	Peptoanaerobacter stomatis strain ACC19a	Bacteria	0.01
2346	1612.1	Lactobacillus farciminis	Bacteria	0.01
2347	796937.4	Peptoanaerobacter stomatis strain CM2	Bacteria	0.01
2348	1496.438	Clostridioides difficile strain VRECD0005	Bacteria	0.01
2349	1496.434	Clostridioides difficile strain VRECD0080	Bacteria	0.01
2350	29379.1	Staphylococcus auricularis strain DSM	Bacteria	0.01
2351	698957	Gardnerella vaginalis 1500E	Bacteria	0.01
2352	1302272	Lactobacillus kimchicus JCM 15530	Bacteria	0.01
2353	904296	Oribacterium sp. oral taxon 108 str. F0425	Bacteria	0.01
2354	1423715	Lactobacillus acidifarinae DSM 19394	Bacteria	0.01
2355	373058.1	Tomato bushy stunt virus satellite RNA	Viruses	0.01
2356	1218113	Kluyvera intermedia NBRC 102594 = ATCC 33110	Bacteria	0.01
2357	1218112	Kluyvera cryocrescens NBRC 102467	Bacteria	0.01
2358	1280.1861	Staphylococcus aureus strain C2485	Bacteria	0.01
2359	1303256.1	Sphingobium sp. DC-2	Bacteria	0.01
2360	99565	Chiba virus	Viruses	0.01
2361	187764.1	Escherichia virus K1-5	Viruses	0.01
2362	698487	Escherichia phage K1-dep(1)	Viruses	0.01
2363	1121315	Terrisporobacter glycolicus ATCC 14880 = DSM 1288	Bacteria	0.01
2364	91753.24	Cucurbit aphid-borne yellows virus isolate CABYV-R-TW82	Viruses	0.01
2365	1745712.1	Anaerococcus sp. Marseille-P2143 strain Marseille-P2143, sp. FC4	Bacteria	0.01
2366	329852.1	Escherichia virus MS2 isolate J20	Viruses	0.01

2367	31770.2	Shallot virus X strain Russian	Viruses	0.01
2368	1131442	Mycobacterium marinum E11	Bacteria	0.01
2369	1400823	Enterococcus faecium UC7256	Bacteria	0.01
2370	1028804	Haemophilus haemolyticus M21127	Bacteria	0.01
2371	1028806	Haemophilus haemolyticus M21639	Bacteria	0.01
2372	1115515	Escherichia vulneris NBRC 102420	Bacteria	0.01
2373	1122997	Acidipropionibacterium jensenii DSM 20535	Bacteria	0.01
2374	1239384	Escherichia phage IME11	Viruses	0.01
2375	857292	Streptococcus intermedius F0395	Bacteria	0.01
2376	347253	Streptococcus salivarius JIM8777	Bacteria	0.01
2377	575599	Lactobacillus fermentum 28-3-CHN	Bacteria	0.01
2378	1156431.1	Streptococcus sp. I-G2 sp. I-G2	Bacteria	0.01
2379	1661745.1	Haemophilus sp. C1	Bacteria	0.01
2380	12295.8	Tobacco rattle virus isolate Rostock	Viruses	0.01
2381	525369	Proteus mirabilis ATCC 29906	Bacteria	0.01
2382	473784.1	Opium poppy mosaic virus isolate PHEL5235	Viruses	0.01
2383	12295.6	Tobacco rattle virus isolate MI-1	Viruses	0.01
2384	11226.4	Human parainfluenza virus 4b strain QLD-01	Viruses	0.01
2385	1008452	Streptococcus mitis SK1073	Bacteria	0.01
2386	1879023.1	Mycobacterium sp. djl-10 sp. djl-10	Bacteria	0.01
2387	76859.1	Fusobacterium nucleatum subsp. animalis strain KCOM	Bacteria	0.01
2388	35703.1	Citrobacter amalonaticus strain L8A	Bacteria	0.01
2389	1813769.1	Salmonella phage 64795_sal3	Viruses	0.01
2390	244366.24	Klebsiella variicola isolate T29A	Bacteria	0.01
2391	1328.2	Streptococcus anginosus strain 1080_SANG	Bacteria	0.01
2392	1391428	Escherichia phage 4MG	Viruses	0.01
2393	1112212	Sphingomonas echinoides ATCC 14820	Bacteria	0.01
2394	1581067.1	Kytococcus sp. HMSC28H12	Bacteria	0.01
2395	228578.6	Youcai mosaic virus isolate Br	Viruses	0.01
2396	889204	Streptococcus infantis ATCC 700779	Bacteria	0.01
2397	338473.1	Actinomyces virus Av1	Viruses	0.01
2398	5082.1	Penicillium roqueforti strain UASWS	Eukaryota	0.01
2399	1685.13	Bifidobacterium breve strain BR-15	Bacteria	0.01
2400	1631871.1	Weissella jogaejeotgali strain FOL01	Bacteria	0.01
2401	1423771	Lactobacillus mucosae DSM 13345	Bacteria	0.01
2402	1608993.1	Pseudomonas sp. DSM 28140	Bacteria	0.01
2403	12274.5	Grapevine fanleaf virus isolate GHu	Viruses	0.01
2404	646010.1	Suakwa aphid-borne yellows virus isolate SABYV-TW19	Viruses	0.01
2405	1316587	Fusobacterium nucleatum CTI-6	Bacteria	0.01
2406	1073373	Streptococcus sanguinis CC94A	Bacteria	0.01
2407	105219.2	Ralstonia mannitolilytica strain SN82F48	Bacteria	0.01
2408	1581069.1	Corynebacterium sp. HMSC29G08	Bacteria	0.01
2409	889206	Streptococcus vestibularis ATCC 49124	Bacteria	0.01
2410	546.15	Citrobacter freundii strain FDAARGOS_61	Bacteria	0.01
2411	28347.3	Apple stem grooving virus isolate M220	Viruses	0.01
2412	1492.2	Clostridium butyricum strain SU1	Bacteria	0.01

2413	1492.5	Clostridium butyricum strain JKY6D1	Bacteria	0.01
2414	1492.8	Clostridium butyricum strain CDC_51208	Bacteria	0.01
2415	1585.4	Lactobacillus delbrueckii subsp. bulgaricus strain LBB.B5	Bacteria	0.01
2416	864563	Selenomonas sp. oral taxon 149 str. 67H29BP	Bacteria	0.01
2417	315405.6	Streptococcus gallolyticus strain VTM2R47	Bacteria	0.01
2418	12169.1	Potato virus S isolate 89.249	Viruses	0.01
2419	1496.31	Clostridioides difficile strain 106	Bacteria	0.01
2420	1122980	Prevotella baroniae DSM 16972 = JCM 13447	Bacteria	0.01
2421	1581075.1	Neisseria sp. HMSC31F04	Bacteria	0.01
2422	546.4	Citrobacter freundii strain GED7749C	Bacteria	0.01
2423	10752.1	Escherichia phage N4	Viruses	0.01
2424	104263.2	Hop latent virus	Viruses	0.01
2425	1756832.2	Phasey bean mild yellows virus isolate NSWCP15	Viruses	0.01
2426	1303.1	Streptococcus oralis strain 918_SORA	Bacteria	0.01
2427	1042417	Brachyspira pilosicoli P43/6/78	Bacteria	0.01
2428	727.66	Haemophilus influenzae strain 839_HINF	Bacteria	0.01
2429	1496.406	Clostridioides difficile strain CD33	Bacteria	0.01
2430	1051503	Bacillus subtilis subsp. spizizenii DV1-B-1	Bacteria	0.01
2431	4959.1	Debaryomyces hansenii	Eukaryota	0.01
2432	1581072.1	Corynebacterium sp. HMSC30G07	Bacteria	0.01
2433	644284	Arcanobacterium haemolyticum DSM 20595	Bacteria	0.01
2434	1655.5	Actinomyces naeslundii strain R8152	Bacteria	0.01
2435	12197.1	Bean yellow mosaic virus	Viruses	0.01
2436	162.1	Treponema phagedenis	Bacteria	0.01
2437	1116231	Streptococcus macedonicus ACA-DC 198	Bacteria	0.01
2438	1401659	Cronobacter sakazakii CMCC 45402	Bacteria	0.01
2439	1923725.1	Wuhan insect virus 21 strain WHCCII13077	Viruses	0.01
2440	1206110.1	Lactobacillus phage phiAQ113	Viruses	0.01
2441	576789.1	Enterobacteria phage JSE	Viruses	0.01
2442	469599	Fusobacterium periodonticum 2_1_31	Bacteria	0.01
2443	1203573.1	Propionibacterium sp. KPL1844	Bacteria	0.01
2444	28348.1	Sweet clover necrotic mosaic virus strain 38	Viruses	0.01
2445	936140	Lactobacillus farciminis KCTC 3681 = DSM 20184	Bacteria	0.01
2446	1527519.1	Escherichia phage Av-05	Viruses	0.01
2447	235443	Cryptococcus neoformans var. grubii H99	Eukaryota	0.01
2448	1035839	Haemophilus sputorum CCUG 13788	Bacteria	0.01
2449	488537	Clostridium perfringens D str. JGS1721	Bacteria	0.01
2450	562.535	Escherichia coli strain 444_ECOL	Bacteria	0.01
2451	673375	Sodalis phage SO1	Viruses	0.01
2452	294746	Meyerozyma guilliermondii ATCC 6260	Eukaryota	0.01
2453	11988.2	Turnip crinkle virus	Viruses	0.01
2454	1414738	Shigella phage pSb-1	Viruses	0.01
2455	556267	Helicobacter winghamensis ATCC BAA-430	Bacteria	0.01
2456	1095749	Pasteurella bettyae CCUG 2042	Bacteria	0.01
2457	1381464.1	Blackberry vein banding associated virus isolate Mississippi1	Viruses	0.01

2458	1496.46	Clostridioides difficile isolate VL_0218	Bacteria	0.01
2459	185891	Human rhinovirus A9	Viruses	0.01
2460	1224748	Solibacillus isronensis B3W22	Bacteria	0.01
2461	185897	Human rhinovirus A24	Viruses	0.01
2462	185896	Human rhinovirus A22	Viruses	0.01
2463	709323.1	Fructobacillus tropaeoli	Bacteria	0.01
2464	102684.1	Streptococcus infantarius strain ICDDRB-NRC-S5	Bacteria	0.01
2465	142843.1	Hop mosaic virus	Viruses	0.01
2466	45634.5	Streptococcus cristatus strain JPIIBBV4	Bacteria	0.01
2467	1121421	Desulfotomaculum aeronauticum DSM 10349	Bacteria	0.01
2468	12470.1	Lucerne transient streak virus isolate LTSV-Can	Viruses	0.01
2469	1580.8	Lactobacillus brevis strain DmCS_003	Bacteria	0.01
2470	1580.9	Lactobacillus brevis	Bacteria	0.01
2471	33759	Citrus tatter leaf virus	Viruses	0.01
2472	33750	Hawaii calicivirus	Viruses	0.01
2473	1768743.1	Blastomonas sp. CCH8-A3	Bacteria	0.01
2474	111970.2	Kyuri green mottle mosaic virus strain Yodo	Viruses	0.01
2475	1232427.1	Corynebacterium ihumii strain GD7	Bacteria	0.01
2476	1552735.1	Lactobacillus phage Ldl1	Viruses	0.01
2477	1124962	Salmonella enterica subsp. enterica serovar Poona str. ATCC BAA-1673	Bacteria	0.01
2478	456999.1	Rhizoctonia solani strain AG3	Eukaryota	0.01
2479	1100043.1	Apis mellifera filamentous virus isolate CH-CO5	Viruses	0.01
2480	565655	Enterococcus casseliflavus EC20	Bacteria	0.01
2481	1871034.1	Propionimicrobium sp. Marseille-P3275 strain Marseille-P3275T	Bacteria	0.01
2482	1768764.1	Streptococcus sp. CCH5-D3	Bacteria	0.01
2483	1581113.1	Corynebacterium sp. HMSC05C01	Bacteria	0.01
2484	1064535	Megasphaera elsdenii DSM 20460	Bacteria	0.01
2485	1790.3	Mycobacterium asiaticum strain 1276495.2	Bacteria	0.01
2486	644.11	Aeromonas hydrophila strain AH-1	Bacteria	0.01
2487	732.2	Aggregatibacter aphrophilus strain W10433	Bacteria	0.01
2488	1923094.2	Hubei picorna-like virus 15 strain QTM27139	Viruses	0.01
2489	57706.2	Citrobacter braakii strain GTA-CB04	Bacteria	0.01
2490	36343.1	Lactococcus phage bIL67	Viruses	0.01
2491	39804.3	Escherichia virus FI strain BR8	Viruses	0.01
2492	537874.1	Streptococcus phage PH15	Viruses	0.01
2493	39804.5	Escherichia virus FI strain BR1	Viruses	0.01
2494	1123249	Selenomonas artemidis DSM 19719	Bacteria	0.01
2495	327277.1	Bifidobacterium crudilactis strain LMG	Bacteria	0.01
2496	1408431	Butyrivibrio proteoclasticus FD2007	Bacteria	0.01
2497	73422.1	Streptococcus phage TP-J34	Viruses	0.01
2498	1444235	Escherichia coli 2-316-03_S4_C2	Bacteria	0.01
2499	71032.3	Grapevine leafroll-associated virus 5 isolate TRAJ1-BR	Viruses	0.01
2500	1226633	Fusobacterium necrophorum subsp. funduliforme B35	Bacteria	0.01
2501	936046	Agaricus bisporus var. bisporus H97	Eukaryota	0.01

2502	1182762.1	Enterococcus sp. C1	Bacteria	0.01
2503	1392849	Escherichia coli M3	Bacteria	0.01
2504	1873990.1	Escherichia phage vB_EcoM_Alf5	Viruses	0.01
2505	1236508	Prevotella aurantiaca JCM 15754	Bacteria	0.01
2506	576791	Escherichia phage wV8	Viruses	0.01
2507	317010.1	Enterococcus canintestini strain DSM	Bacteria	0.01
2508	796937.2	Peptoanaerobacter stomatis strain CM5	Bacteria	0.01
2509	546270	Gemella haemolysans ATCC 10379	Bacteria	0.01
2510	12267.1	Red clover necrotic mosaic virus	Viruses	0.01
2511	546275	Fusobacterium periodonticum ATCC 33693	Bacteria	0.01
2512	1295140	Haemophilus influenzae CGSHiCZ412602	Bacteria	0.01
2513	1285582	Lactobacillus sakei subsp. sakei LS25	Bacteria	0.01
2514	37961.1	Atkinsonella hypoxylon virus	Viruses	0.01
2515	515622	Butyrivibrio proteoclasticus B316	Bacteria	0.01
2516	331679.1	Pediococcus stilesii strain DSM	Bacteria	0.01
2517	255238.1	Fragaria chiloensis latent virus	Viruses	0.01
2518	550.203	Enterobacter cloacae strain e1639	Bacteria	0.01
2519	12319.5	Apple mosaic virus	Viruses	0.01
2520	1823756.1	Actinomycetaceae bacterium BA112	Bacteria	0.01
2521	1768770.1	Caulobacter sp. CCH5-E12	Bacteria	0.01
2522	33964.3	Leuconostoc citreum	Bacteria	0.01
2523	1233383.1	Human cosavirus isolate Cosavirus_Amsterdam_1994	Viruses	0.01
2524	1033736.1	Brevibacterium senegalense sp. JC43	Bacteria	0.01
2525	1914546	Norovirus Hu/USA/2016/GI.P9_GI.9/SC6350	Viruses	0.01
2526	563194	Pediococcus acidilactici 7_4	Bacteria	0.01
2527	484020	Bifidobacterium bifidum BGN4	Bacteria	0.01
2528	1505.13	Paeniclostridium sordellii strain W2922	Bacteria	0.01
2529	997353	Prevotella pallens ATCC 700821	Bacteria	0.01
2530	561177	Anaerococcus hydrogenalis DSM 7454	Bacteria	0.01
2531	457403	Fusobacterium nucleatum subsp. animalis 11_3_2	Bacteria	0.01
2532	28375.1	Soil-borne wheat mosaic virus	Viruses	0.01
2533	56407.1	Hanseniaspora occidentalis	Eukaryota	0.01
2534	167161.6	Strawberry mottle virus	Viruses	0.01
2535	167161.2	Strawberry mottle virus isolate NSper17	Viruses	0.01
2536	167161.3	Strawberry mottle virus isolate NSper51	Viruses	0.01
2537	1305618	Porphyromonas crevioricanis JCM 13913	Bacteria	0.01
2538	264483.1	Phaffia rhodozyma	Eukaryota	0.01
2539	2371.8	Xylella fastidiosa strain CFBP8073	Bacteria	0.01
2540	1739264.1	Corynebacterium sp. HMSC065D07	Bacteria	0.01
2541	1439318	Citrobacter freundii MGH 56	Bacteria	0.01
2542	451754	Clostridium perfringens B str. ATCC 3626	Bacteria	0.01
2543	451755	Clostridium perfringens E str. JGS1987	Bacteria	0.01
2544	665550.1	Dietzia alimentaria strain BP	Bacteria	0.01
2545	68033.1	Carrot mottle virus	Viruses	0.01
2546	1161906.1	Weissella phage phiYS61	Viruses	0.01
2547	1329838.1	Enterobacter sp. BIDMC 26	Bacteria	0.01
2548	1451189	Corynebacterium falsenii DSM 44353	Bacteria	0.01

2549	979982.1	Leuconostoc sp. C2 sp. C2	Bacteria	0.01
2550	590403.1	Red clover vein mosaic virus isolate NZ	Viruses	0.01
2551	585.1	Proteus vulgaris strain CSUR	Bacteria	0.01
2552	36745.1	Clostridium saccharoperbutylacetonicum strain N1-504	Bacteria	0.01
2553	571.67	Klebsiella oxytoca strain CHS143	Bacteria	0.01
2554	861454	Lachnospiraceae bacterium oral taxon 082 str. F0431	Bacteria	0.01
2555	1444222	Escherichia coli 3-105-05_S4_C1	Bacteria	0.01
2556	74381.1	Undaria pinnatifida	Eukaryota	0.01
2557	1505.8	Paeniclostridium sordellii strain SSCC32135	Bacteria	0.01
2558	37206.1	Helicoverpa armigera stunt virus	Viruses	0.01
2559	1923266.1	Hubei tombus-like virus 2 strain WHSFII19265	Viruses	0.01
2560	1416754	Klebsiella michiganensis H1g	Bacteria	0.01
2561	1171373	Acidipropionibacterium acidipropionici ATCC 4875	Bacteria	0.01
2562	35350.3	Apple stem pitting virus isolate PR1	Viruses	0.01
2563	10829.2	Squash leaf curl virus	Viruses	0.01
2564	817.1	Bacteroides fragilis strain DCMSKEJBY0001B	Bacteria	0.01
2565	1457184	Bifidobacterium longum subsp. longum 72B	Bacteria	0.01
2566	1408287	Fusobacterium nucleatum W1481	Bacteria	0.01
2567	702438	Prevotella oulorum F0390	Bacteria	0.01
2568	264076.1	Horseradish latent virus	Viruses	0.01
2569	851.3	Fusobacterium nucleatum strain MJR7757B	Bacteria	0.01
2570	1229751.1	Lactococcus phage BM13	Viruses	0.01
2571	1965306.1	Lagenaria siceraria endornavirus-Hubei isolate JZ	Viruses	0.01
2572	12055.2	Tobacco necrosis virus A isolate Velence	Viruses	0.01
2573	1805471.1	Clostridium sp. Marseille-P2415 sp. Marseille-P2415	Bacteria	0.01
2574	1423822	Lactobacillus coryniformis subsp. torquens DSM 20004 = KCTC 3535	Bacteria	0.01
2575	1050107.1	Lactobacillus delbrueckii subsp. sunkii strain JCM	Bacteria	0.01
2576	1441736	Fusobacterium necrophorum BFTR-2	Bacteria	0.01
2577	938293.1	Anaerococcus provenciensis sp. 9402080	Bacteria	0.01
2578	1175299	Dickeya zeae ZJU1202	Bacteria	0.01
2579	1522060.1	Pantoea sp. 3.5.1	Bacteria	0.01
2580	1005705	Streptococcus infantis SK1076	Bacteria	0.01
2581	368736.1	Maracuja mosaic virus	Viruses	0.01
2582	1496.7	Clostridioides difficile isolate VL_0350	Bacteria	0.01
2583	439334.34	Mycobacterium avium subsp. hominissuis strain MAH-P-0913	Bacteria	0.01
2584	35841.1	Bacillus thermoamylovorans strain 1A1	Bacteria	0.01
2585	326202.1	Vanilla distortion mosaic virus isolate VDMV-Cor	Viruses	0.01
2586	1382301	Lactobacillus plantarum EGD-AQ4	Bacteria	0.01
2587	553190	Gardnerella vaginalis 409-05	Bacteria	0.01
2588	553198	Propionibacterium acidifaciens F0233	Bacteria	0.01
2589	1502.4	Clostridium perfringens strain CP15	Bacteria	0.01

2590	471872	Streptococcus infantarius subsp. infantarius ATCC BAA-102	Bacteria	0.01
2591	909827.2	Pepper vein yellows virus isolate 12KNX1	Viruses	0.01
2592	699248	Streptococcus ratti FA-1 = DSM 20564	Bacteria	0.01
2593	12230.2	Turnip mosaic virus	Viruses	0.01
2594	1134687.39	Klebsiella michiganensis strain MGH	Bacteria	0.01
2595	12431.1	Garlic virus C	Viruses	0.01
2596	1071395	Bacillus coagulans XZL4	Bacteria	0.01
2597	1778.4	Mycobacterium gordonae strain 1275229.4	Bacteria	0.01
2598	1381091	Streptococcus equi subsp. zooepidemicus SzAM60	Bacteria	0.01
2599	1302.3	Streptococcus gordonii strain Channon	Bacteria	0.01
2600	143387.17	Fusobacterium necrophorum subsp. funduliforme strain F1250	Bacteria	0.01
2601	1196034.1	Klebsiella sp. 10982	Bacteria	0.01
2602	1739315.1	Globicatella sp. HMSC072A10	Bacteria	0.01
2603	185893	Human rhinovirus A13	Viruses	0.01
2604	37662.1	Brettanomyces anomalus	Eukaryota	0.01
2605	933356	Enterococcus faecium E4452	Bacteria	0.01
2606	883066	Actinobaculum massiliense ACS-171-V-Col2	Bacteria	0.01
2607	1400137	Citrobacter freundii UCI 32	Bacteria	0.01
2608	1122171	Leptotrichia hofstadii DSM 21651	Bacteria	0.01
2609	1675607.1	Klebsiella phage Matisse	Viruses	0.01
2610	315405.1	Streptococcus gallolyticus strain ICDDRB-NRC-S1	Bacteria	0.01
2611	562.1112	Escherichia coli strain upec-274	Bacteria	0.01
2612	1051660	Lactobacillus casei UW1	Bacteria	0.01
2613	425279.3	Rehmannia mosaic virus isolate Shanxi	Viruses	0.01
2614	28037.19	Streptococcus mitis strain SVGS_061	Bacteria	0.01
2615	28037.15	Streptococcus mitis strain SK642	Bacteria	0.01
2616	28037.16	Streptococcus mitis strain SK1126	Bacteria	0.01
2617	1358413	Lactobacillus plantarum AY01	Bacteria	0.01
2618	131083.2	Turnip yellows virus isolate WA-1	Viruses	0.01
2619	523844	Methanosarcina thermophila TM-1	Archaea	0.01
2620	334413	Finegoldia magna ATCC 29328	Bacteria	0.01
2621	638302	Selenomonas flueggei ATCC 43531	Bacteria	0.01
2622	1509.18	Clostridium sporogenes strain CDC23284	Bacteria	0.01
2623	1870933.1	Enterobacter cloacae complex sp. 20432	Bacteria	0.01
2624	752790	Escherichia coli CUMT8	Bacteria	0.01
2625	1914861.1	Enterobacter sp. Sa187	Bacteria	0.01
2626	1849383.1	Psychrobacter sp. SHUES1	Bacteria	0.01
2627	1932006.3	Chicken associated smacovirus strain RS/BR/2015/1	Viruses	0.01
2628	1123306	Streptococcus marimammalium DSM 18627	Bacteria	0.01
2629	1423828	Lactobacillus kefiranofaciens subsp. kefirgranum DSM 10550 = JCM 8572	Bacteria	0.01
2630	12165.2	Chrysanthemum virus B isolate Uttarakhand	Viruses	0.01
2631	253702.1	Opuntia virus X isolate nopal	Viruses	0.01
2632	1096935.1	Veillonella sp. OK1	Bacteria	0.01

2633	1888167.1	Enterobacter sp. ku-bf2	Bacteria	0.01
2634	391774	Desulfovibrio vulgaris DP4	Bacteria	0.01
2635	1325933.1	Clostridium polynesiense sp. MS1	Bacteria	0.01
2636	682148	Gardnerella vaginalis 5-1	Bacteria	0.01
2637	1930302.1	Chicken stool-associated circular virus strain RS/BR/2015	Viruses	0.01
2638	90371.59	Salmonella enterica subsp. enterica serovar Typhimurium strain CFSAN033859	Bacteria	0.01
2639	443906	Clavibacter michiganensis subsp. michiganensis NCPPB 382	Bacteria	0.01
2640	1496.62	Clostridioides difficile strain CD105KSE9	Bacteria	0.01
2641	1613.1	Lactobacillus fermentum strain 90	Bacteria	0.01
2642	445334	Clostridium perfringens C str. JGS1495	Bacteria	0.01
2643	91353.1	Campylobacter hyointestinalis subsp. lawsonii strain LMG	Bacteria	0.01
2644	1739611.1	Lactobacillus phage iLp1308	Viruses	0.01
2645	879297	Lactobacillus iners LactinV 01V1-a	Bacteria	0.01
2646	1776109.1	Goose dicistrovirus isolate UW1	Viruses	0.01
2647	562.705	Escherichia coli strain upec-282	Bacteria	0.01
2648	1907766.1	Pseudomonas sp. BS-2016 strain AU14541	Bacteria	0.01
2649	796943	Oribacterium parvum ACB1	Bacteria	0.01
2650	1211480.1	Persimmon virus A	Viruses	0.01
2651	425010.1	Botryotinia fuckeliana partitivirus 1	Viruses	0.01
2652	1385940	Bifidobacterium breve JCM 7019	Bacteria	0.01
2653	1123721	Weissella koreensis KCTC 3621	Bacteria	0.01
2654	1246.4	Leuconostoc lactis strain WiKim40	Bacteria	0.01
2655	888810	Streptococcus sanguinis SK115	Bacteria	0.01
2656	28037.1	Streptococcus mitis strain SK608	Bacteria	0.01
2657	1118963.2	Arthrobacter sp. Rue61a	Bacteria	0.01
2658	33010.7	Cutibacterium avidum strain DPC	Bacteria	0.01
2659	1840644.1	Psophocarpus tetragonolobus endornavirus	Viruses	0.01
2660	35841.3	Bacillus thermoamylovorans strain B4064	Bacteria	0.01
2661	5412.1	Cystofilobasidium capitatum	Eukaryota	0.01
2662	469621	Fusobacterium periodonticum 1_1_41FAA	Bacteria	0.01
2663	1141136.1	Cronobacter phage vB_CsaM_GAP32	Viruses	0.01
2664	1640536.1	Arthrospira sp. TJSD091	Bacteria	0.01
2665	1169350.1	Citrobacter sp. KTE32	Bacteria	0.01
2666	571.41	Klebsiella oxytoca strain 2880STDY5682436	Bacteria	0.01
2667	35281.2	Paprika mild mottle virus	Viruses	0.01
2668	46076.3	Artichoke latent virus isolate FR37	Viruses	0.01
2669	1280.449	Staphylococcus aureus strain C2304	Bacteria	0.01
2670	28038.7	Lactobacillus curvatus strain WiKim52	Bacteria	0.01
2671	1050903.3	Pepper cryptic virus 2 isolate HW-01	Viruses	0.01
2672	1581133.1	Actinomyces sp. HMSC08A09	Bacteria	0.01
2673	147712.1	Rhinovirus B strain HRV-B06_p011_sT0384_2007	Viruses	0.01
2674	1305.7	Streptococcus sanguinis strain 2908	Bacteria	0.01
2675	1305.6	Streptococcus sanguinis strain 216_SSAN	Bacteria	0.01

2676	861360	Glutamicibacter arilaitensis Re117	Bacteria	0.01
2677	1768759.1	Bradyrhizobium sp. CCH4-A6	Bacteria	0.01
2678	1423792	Lactobacillus perolens DSM 12744	Bacteria	0.01
2679	936563.1	Fusobacterium sp. CM22	Bacteria	0.01
2680	185928	Human rhinovirus A73	Viruses	0.01
2681	1411141	Serratia ficaria NBRC 102596	Bacteria	0.01
2682	1923133.1	Hubei picorna-like virus 51 strain QTM27291	Viruses	0.01
2683	12348.1	Lactobacillus phage LL-H	Viruses	0.01
2684	1200547.1	Prevotella sp. RM4	Bacteria	0.01
2685	1121268	Campylobacter curvus DSM 6644	Bacteria	0.01
2686	272633	Mycoplasma penetrans HF-2	Bacteria	0.01
2687	1441735	Fusobacterium necrophorum DAB	Bacteria	0.01
2688	910314	Dialister microaerophilus UPII 345-E	Bacteria	0.01
2689	1386970	Clostridium perfringens JJC	Bacteria	0.01
2690	1599.4	Lactobacillus sakei strain RI-394	Bacteria	0.01
2691	12305.35	Cucumber mosaic virus isolate Rom	Viruses	0.01
2692	883067	Actinotignum schaalii FB123-CNA-2	Bacteria	0.01
2693	562.803	Escherichia coli strain G5	Bacteria	0.01
2694	1401064	Corynebacterium tuscaniense DNF00037	Bacteria	0.01
2695	1151428	Clostridioides difficile P59	Bacteria	0.01
2696	1686394.1	Enterobacter sp. MGH128	Bacteria	0.01
2697	525271	Enterococcus faecalis ATCC 29200	Bacteria	0.01
2698	562.937	Escherichia coli strain upec-34	Bacteria	0.01
2699	883119	Klebsiella oxytoca 10-5244	Bacteria	0.01
2700	883114	Helcococcus kunzii ATCC 51366	Bacteria	0.01
2701	132477.1	Kalanchoe latent virus	Viruses	0.01
2702	633.1	Yersinia pseudotuberculosis strain CEB14_0017	Bacteria	0.01
2703	888811	Streptococcus sanguinis SK150	Bacteria	0.01
2704	888813	Streptococcus sanguinis SK330	Bacteria	0.01
2705	1406147	Norovirus Hu/GI.2/Jingzhou/2013401/CHN	Viruses	0.01
2706	1549858.2	Sphingomonas taxi strain 30a	Bacteria	0.01
2707	582.2	Morganella morganii strain MRSN22709	Bacteria	0.01
2708	1923594.2	Wenzhou picorna-like virus 10 strain WZRBX43164	Viruses	0.01
2709	1161421	Streptococcus oralis SK304	Bacteria	0.01
2710	246432.4	Staphylococcus equorum strain 738_7	Bacteria	0.01
2711	656083.1	Barley yellow striate mosaic virus strain Hebei	Viruses	0.01
2712	1134805	Enterococcus faecium 504	Bacteria	0.01
2713	12402.1	Streptococcus phage EJ-1	Viruses	0.01
2714	1280703	Butyrivibrio fibrisolvens MD2001	Bacteria	0.01
2715	1280701	Bifidobacterium pseudolongum AGR2145	Bacteria	0.01
2716	1365959	Bifidobacterium animalis subsp. animalis ATCC 27672	Bacteria	0.01
2717	575604	Lactobacillus gasseri SV-16A-US	Bacteria	0.01
2718	544580.4	Actinomyces oris strain R23275	Bacteria	0.01
2719	544580.7	Actinomyces oris strain A19A-1	Bacteria	0.01
2720	1770265.1	Alfalfa enamovirus-1 isolate Manfredi	Viruses	0.01
2721	1307832	Tannerella forsythia 3313	Bacteria	0.01

2722	681573	Streptococcus equi subsp. zooepidemicus BHS5	Bacteria	0.01
2723	294.4	Pseudomonas fluorescens strain ATCC	Bacteria	0.01

Table S3: All species identified in 10,000 human stool samples

	Taxonomy ID	Species name	SuperKingdom	Prevalence in 10,000 samples, %
1	821	Bacteroides vulgatus	Bacteria	97.13
2	470	Acinetobacter baumannii	Bacteria	96.82
3	853	Faecalibacterium prausnitzii	Bacteria	96.37
4	820	Bacteroides uniformis	Bacteria	95.38
5	84112	Eggerthella lenta	Bacteria	91.76
6	39488	[Eubacterium] hallii	Bacteria	91.51
7	169435	Anaerotruncus colihominis	Bacteria	91.42
8	1650661	Clostridium phoceensis	Bacteria	90.84
9	39778	Veillonella dispar	Bacteria	88.99
10	1150298	Fusicatenibacter saccharivorans	Bacteria	87.26
11	823	Parabacteroides distasonis	Bacteria	87.07
12	817	Bacteroides fragilis	Bacteria	85.93
13	214856	Alistipes finegoldii	Bacteria	83.35
14	301301	Roseburia hominis	Bacteria	83.35
15	40520	Blautia obeum	Bacteria	80.81
16	28116	Bacteroides ovatus	Bacteria	80.13
17	28117	Alistipes putredinis	Bacteria	79.90
18	33039	[Ruminococcus] torques	Bacteria	79.50
19	818	Bacteroides thetaiotaomicron	Bacteria	79.32
20	1737424	Blautia massiliensis	Bacteria	78.86
21	35833	Bilophila wadsworthia	Bacteria	78.74
22	28118	Odoribacter splanchnicus	Bacteria	78.07
23	1715004	Clostridiales bacterium KLE1615	Bacteria	76.47
24	328814	Alistipes shahii	Bacteria	76.45
25	39485	[Eubacterium] eligens	Bacteria	76.01
26	88431	Dorea longicatena	Bacteria	75.73
27	46503	Parabacteroides merdae	Bacteria	75.09
28	39491	[Eubacterium] rectale	Bacteria	74.80
29	357276	Bacteroides dorei	Bacteria	74.59
30	410072	Coprococcus comes	Bacteria	74.49
31	166486	Roseburia intestinalis	Bacteria	74.28
32	1519439	Oscillibacter sp. ER4	Bacteria	73.94
33	1522	[Clostridium] innocuum	Bacteria	73.77
34	360807	Roseburia inulinivorans	Bacteria	73.50
35	214851	Subdoligranulum variabile	Bacteria	72.43
36	446660	Adlercreutzia equolifaciens	Bacteria	70.41
37	1535	[Clostridium] leptum	Bacteria	70.30
38	47678	Bacteroides caccae	Bacteria	68.21
39	301302	Roseburia faecis	Bacteria	67.03
40	1297617	Intestinimonas butyriciproducens	Bacteria	66.95
41	471189	Gordonibacter pamelaeae	Bacteria	65.09
42	418240	Blautia wexlerae	Bacteria	64.05
43	1697794	Clostridia bacterium UC5.1-1D1	Bacteria	63.33
44	74426	Collinsella aerofaciens	Bacteria	63.25
45	39490	Eubacterium ramulus	Bacteria	63.11

46	12239	Pepper mild mottle virus	Viruses	62.57
47	1118061	Alistipes obesi	Bacteria	62.41
48	1871035	Ruminococcus sp. Marseille-P3213	Bacteria	61.59
49	1352	Enterococcus faecium	Bacteria	59.67
50	46506	Bacteroides stercoris	Bacteria	59.62
51	1288121	Alistipes senegalensis	Bacteria	59.38
52	328813	Alistipes onderdonkii	Bacteria	57.51
53	544645	Butyricimonas virosa	Bacteria	57.45
54	39486	Dorea formicigenerans	Bacteria	56.78
55	204516	Bacteroides massiliensis	Bacteria	56.41
56	1917876	Blautia sp. Marseille-P3087	Bacteria	54.65
57	1673721	Intestinimonas massiliensis	Bacteria	54.48
58	246787	Bacteroides cellulosilyticus	Bacteria	54.15
59	649756	Anaerostipes hadrus	Bacteria	54.09
60	39492	[Eubacterium] siraeum	Bacteria	53.84
61	552398	Ruminococcaceae bacterium D16	Bacteria	53.81
62	1841867	Phocea massiliensis	Bacteria	53.60
63	46228	Ruminococcus lactaris	Bacteria	52.95
64	626932	Alistipes indistinctus	Bacteria	52.71
65	1232459	Oscillospiraceae bacterium VE202-24	Bacteria	52.69
66	1160721	Ruminococcus bicirculans	Bacteria	52.47
67	239935	Akkermansia muciniphila	Bacteria	51.28
68	487174	Barnesiella intestinihominis	Bacteria	50.92
69	1432052	Eisenbergiella tayi	Bacteria	50.61
70	338188	Bacteroides finegoldii	Bacteria	50.31
71	329854	Bacteroides intestinalis	Bacteria	50.25
72	469610	Burkholderiales bacterium 1_1_47	Bacteria	49.56
73	29348	[Clostridium] spiroforme	Bacteria	49.14
74	28052	Lachnospira pectinoschiza	Bacteria	48.84
75	84135	Gemella sanguinis	Bacteria	48.02
76	39496	Eubacterium ventriosum	Bacteria	48.00
77	1095771	Ruminococcus sp. JC304	Bacteria	47.35
78	53443	Blautia hydrogenotrophica	Bacteria	43.97
79	665956	Subdoligranulum sp. 4_3_54A2FAA	Bacteria	43.55
80	487175	Parasutterella excrementihominis	Bacteria	43.55
81	1470347	Alistipes ihumii	Bacteria	43.30
82	1703332	Lachnospiraceae bacterium TF01-11	Bacteria	43.21
83	1504823	bacterium LF-3	Bacteria	42.51
84	908612	Alistipes sp. HGB5	Bacteria	42.51
85	292800	Flavonifractor plautii	Bacteria	42.43
86	28111	Bacteroides eggerthii	Bacteria	42.17
87	1496	Clostridioides difficile	Bacteria	41.84
88	371601	Bacteroides xylanisolvens	Bacteria	41.60
89	665949	Tannerella sp. 6_1_58FAA_CT1	Bacteria	41.22
90	106588	Pseudoflavonifractor capillosus	Bacteria	41.19
91	450746	Coprobacillus sp. 8_1_38FAA	Bacteria	40.56
92	658087	Lachnospiraceae bacterium 7_1_58FAA	Bacteria	39.20
93	387661	Parabacteroides johnsonii	Bacteria	38.96

94	40545	Sutterella wadsworthensis	Bacteria	38.55
95	61171	Holdemania filiformis	Bacteria	38.48
96	154046	Hungatella hathewayi	Bacteria	38.27
97	1720200	Anaerotruncus rubiinfantis	Bacteria	38.19
98	411486	Clostridium sp. M62/1	Bacteria	37.78
99	33043	Coprococcus eutactus	Bacteria	37.47
100	1499682	Alistipes sp. AL-1	Bacteria	37.15
101	585543	Bacteroides sp. D20	Bacteria	37.00
102	1232439	Clostridiales bacterium VE202-03	Bacteria	36.73
103	1720194	Clostridium sp. AT4	Bacteria	35.95
104	33038	[Ruminococcus] gnavus	Bacteria	35.90
105	1739298	Bacteroides sp. HMSC067B03	Bacteria	33.55
106	1750560	Parabacteroides sp. SN4	Bacteria	33.50
107	1232453	Clostridiales bacterium VE202-21	Bacteria	33.30
108	1232438	Clostridiales bacterium VE202-01	Bacteria	33.14
109	649724	Clostridium sp. ATCC BAA-442	Bacteria	32.80
110	154288	Turicibacter sanguinis	Bacteria	32.57
111	1550024	Ruthenibacterium lactatiformans	Bacteria	32.08
112	626929	Bacteroides clarus	Bacteria	31.85
113	1697793	Clostridia bacterium UC5.1-1E11	Bacteria	31.74
114	742722	Collinsella sp. 4_8_47FAA	Bacteria	31.43
115	291645	Bacteroides nordii	Bacteria	30.62
116	457412	Ruminococcus sp. 5_1_39BFAA	Bacteria	30.13
117	454154	Paraprevotella clara	Bacteria	29.87
118	1871020	Clostridium sp. Marseille-P3244	Bacteria	28.76
119	1871018	Angelakisella massiliensis	Bacteria	28.62
120	1852384	Ruminococcaceae bacterium Marseille-P2963	Bacteria	28.40
121	1531	[Clostridium] clostridioforme	Bacteria	28.28
122	1308	Streptococcus thermophilus	Bacteria	28.16
123	55565	Actinomyces graevenitzii	Bacteria	27.79
124	454155	Paraprevotella xylaniphila	Bacteria	27.73
125	208479	[Clostridium] bolteae	Bacteria	27.73
126	1776382	Neglecta timonensis	Bacteria	27.46
127	562	Escherichia coli	Bacteria	27.40
128	328812	Parabacteroides goldsteinii	Bacteria	27.15
129	33035	Blautia producta	Bacteria	27.11
130	40519	Ruminococcus callidus	Bacteria	27.08
131	1211417	uncultured phage crAssphage	Viruses	26.67
132	1870991	Massilioclostridium coli	Bacteria	26.41
133	1776384	Emergencia timonensis	Bacteria	26.10
134	1470345	Bacteroides timonensis	Bacteria	25.96
135	214853	Anaerofustis stercorihominis	Bacteria	25.78
136	457389	Bacteroides sp. 3_1_13	Bacteria	25.52
137	1816676	Alistipes sp. Marseille-P2431	Bacteria	25.21
138	1627893	Ruminococcaceae bacterium cv2	Bacteria	25.08
139	1163670	Bacteroides sp. 14(A)	Bacteria	25.00
140	310298	Bacteroides coprocola	Bacteria	24.97
141	469592	Bacteroides sp. 3_1_19	Bacteria	24.83

142	291644	Bacteroides salyersiae	Bacteria	24.49
143	165179	Prevotella copri	Bacteria	24.39
144	39484	Butyricicoccus desmolans	Bacteria	24.38
145	658662	Parabacteroides sp. D26	Bacteria	24.36
146	563193	Parabacteroides sp. D13	Bacteria	23.93
147	29347	[Clostridium] scindens	Bacteria	23.91
148	45851	Butyrivibrio crossotus	Bacteria	23.91
149	1852366	Holdemania sp. Marseille-P2844	Bacteria	23.81
150	1697787	Clostridia bacterium UC5.1-1D10	Bacteria	23.63
151	626940	Phascolarctobacterium succinatutens	Bacteria	23.61
152	12241	Tobacco mild green mosaic virus	Viruses	23.52
153	160404	[Clostridium] lactatifermentans	Bacteria	23.31
154	544644	Butyricimonas synergistica	Bacteria	22.96
155	341220	Lactonifactor longoviformis	Bacteria	22.74
156	100886	Catenibacterium mitsuokai	Bacteria	22.05
157	1735	Holdemanella biformis	Bacteria	21.82
158	12253	Tomato mosaic virus	Viruses	21.63
159	556259	Bacteroides sp. D2	Bacteria	21.38
160	2173	Methanobrevibacter smithii	Archaea	21.15
161	936548	Actinomyces sp. ICM47	Bacteria	20.98
162	1226324	Blautia sp. KLE 1732	Bacteria	20.52
163	457393	Bacteroides sp. 4_1_36	Bacteria	20.25
164	218538	Dialister invisus	Bacteria	20.17
165	1473216	Senegalimassilia anaerobia	Bacteria	19.57
166	457395	Bacteroides sp. 9_1_42FAA	Bacteria	19.06
167	1468449	Holdemania massiliensis	Bacteria	18.92
168	411489	Clostridium sp. L2-50	Bacteria	18.88
169	1465754	Alistipes timonensis	Bacteria	18.85
170	1720300	Ruminococcus sp. AT10	Bacteria	18.39
171	457394	Bacteroides sp. 4_3_47FAA	Bacteria	18.17
172	501571	Butyricicoccus pullicaecorum	Bacteria	17.83
173	847	Oxalobacter formigenes	Bacteria	17.78
174	693988	Bilophila sp. 4_1_30	Bacteria	17.76
175	1226325	Clostridium sp. KLE 1755	Bacteria	17.72
176	84026	[Clostridium] methylpentosum	Bacteria	17.29
177	1574262	Sutterella sp. KLE1602	Bacteria	17.25
178	376806	Bacteroides gallinarum	Bacteria	17.21
179	1870993	Tyzzerella sp. Marseille-P3062	Bacteria	17.05
180	1739319	Bacteroides sp. HMSC068A09	Bacteria	16.66
181	438033	Ruminococcus gauvreauii	Bacteria	16.59
182	1280669	Dorea sp. AGR2135	Bacteria	16.46
183	1078089	Bacteroides sp. HPS0048	Bacteria	16.45
184	216816	Bifidobacterium longum	Bacteria	16.01
185	457390	Bacteroides sp. 3_1_23	Bacteria	15.74
186	270498	Catabacter hongkongensis	Bacteria	15.70
187	1232443	Clostridiales bacterium VE202-13	Bacteria	15.56
188	1232457	Clostridiales bacterium VE202-27	Bacteria	15.48
189	1653435	Clostridium sp. BR31	Bacteria	15.43

190	387090	Bacteroides coprophilus	Bacteria	15.29
191	457391	Bacteroides sp. 3_1_33FAA	Bacteria	15.06
192	29361	Tyzzerella nexilis	Bacteria	14.64
193	112229	Pepino mosaic virus	Viruses	14.57
194	658655	Lachnospiraceae bacterium 1_4_56FAA	Bacteria	14.54
195	469590	Bacteroides sp. 2_2_4	Bacteria	14.31
196	310297	Bacteroides plebeius	Bacteria	14.21
197	105841	Anaerostipes caccae	Bacteria	14.20
198	674529	Bacteroides faecis	Bacteria	14.13
199	384638	[Bacteroides] pectinophilus	Bacteria	14.02
200	457415	Synergistes sp. 3_1_syn1	Bacteria	13.94
201	910311	Eggerthella sp. HGA1	Bacteria	13.94
202	1650663	Fournierella massiliensis	Bacteria	13.45
203	363265	Prevotella stercorea	Bacteria	13.19
204	871324	Bacteroides stercorirosoris	Bacteria	13.17
205	34073	Variovorax paradoxus	Bacteria	13.08
206	592978	Ruminococcus faecis	Bacteria	12.99
207	1841855	Bacteroides sp. Marseille-P2653	Bacteria	12.97
208	1745713	Bariatricus massiliensis	Bacteria	12.65
209	1574263	Candidatus Stoquefichus sp. KLE1796	Bacteria	12.63
210	1099853	Coprobacter fastidiosus	Bacteria	12.40
211	469589	Bacteroides sp. 2_1_33B	Bacteria	12.40
212	1512	[Clostridium] symbiosum	Bacteria	12.26
213	1680	Bifidobacterium adolescentis	Bacteria	12.25
214	1907659	Blautia sp. Marseille-P3201T	Bacteria	11.90
215	1805476	Blautia sp. Marseille-P2398	Bacteria	11.75
216	1034346	Dielma fastidiosa	Bacteria	11.73
217	1852361	Actinomyces sp. Marseille-P2825	Bacteria	11.71
218	28901	Salmonella enterica	Bacteria	11.71
219	1796613	Bacteroides caecimuris	Bacteria	11.59
220	901	Desulfovibrio piger	Bacteria	11.48
221	333367	[Clostridium] asparagiforme	Bacteria	11.29
222	1681	Bifidobacterium bifidum	Bacteria	11.28
223	35281	Paprika mild mottle virus	Viruses	11.23
224	469586	Bacteroides sp. 1_1_6	Bacteria	10.86
225	712888	Actinobaculum sp. oral taxon 183	Bacteria	10.86
226	658659	Erysipelotrichaceae bacterium 3_1_53	Bacteria	10.66
227	12172	Shallot latent virus	Viruses	10.54
228	341225	[Clostridium] saccharogumia	Bacteria	10.49
229	1161942	Ruminococcus champanellensis	Bacteria	10.48
230	147207	Collinsella intestinalis	Bacteria	10.36
231	626937	Christensenella minuta	Bacteria	10.21
232	469614	Erysipelotrichaceae bacterium 6_1_45	Bacteria	10.21
233	469593	Bacteroides sp. 3_1_40A	Bacteria	10.17
234	1309	Streptococcus mutans	Bacteria	9.92
235	261299	Intestinibacter bartlettii	Bacteria	9.88
236	412467	Entamoeba nuttalli	Eukaryota	9.87
237	457387	Bacteroides sp. 1_1_30	Bacteria	9.64

238	469591	Parabacteroides sp. 20_3	Bacteria	9.45
239	28025	Bifidobacterium animalis	Bacteria	9.36
240	626930	Bacteroides fluxus	Bacteria	9.23
241	1188792	Phaseolus vulgaris endornavirus 1	Viruses	8.90
242	1852370	Prevotellamassilia timonensis	Bacteria	8.84
243	1776379	Prevotella sp. KHD1	Bacteria	8.72
244	1871006	Bacteroides sp. Marseille-P3132	Bacteria	8.71
245	168384	Marvinbryantia formatexigens	Bacteria	8.54
246	89153	[Clostridium] hylemonae	Bacteria	8.43
247	658089	Lachnospiraceae bacterium 5_1_63FAA	Bacteria	8.33
248	1871021	Lachnoclostridium phocaeense	Bacteria	8.30
249	1260	Finegoldia magna	Bacteria	8.29
250	37733	Prunus necrotic ringspot virus	Viruses	8.22
251	876	Desulfovibrio desulfuricans	Bacteria	7.97
252	1506471	Sutterellaceae bacterium ND3	Bacteria	7.92
253	143393	[Eubacterium] sulci	Bacteria	7.84
254	658086	Lachnospiraceae bacterium 3_1_57FAA_CT1	Bacteria	7.81
255	1841856	Bacteroides mediterraneensis	Bacteria	7.77
256	12235	Cucumber green mottle mosaic virus	Viruses	7.73
257	626931	Bacteroides oleiciplenus	Bacteria	7.73
258	1232444	Clostridiales bacterium VE202-15	Bacteria	7.67
259	574930	Parabacteroides gordonii	Bacteria	7.56
260	31971	[Eubacterium] dolichum	Bacteria	7.55
261	1318	Streptococcus parasanguinis	Bacteria	7.54
262	665950	Lachnospiraceae bacterium 3_1_46FAA	Bacteria	7.50
263	36834	Clostridium celatum	Bacteria	7.49
264	1852381	Sutterellaceae bacterium Marseille-P2968	Bacteria	7.48
265	457421	Clostridiales bacterium 1_7_47FAA	Bacteria	7.42
266	1245	Leuconostoc mesenteroides	Bacteria	7.30
267	1903263	Traorella massiliensis	Bacteria	7.29
268	1917883	Bacteroides sp. Marseille-P3166	Bacteria	7.16
269	85831	Bacteroides acidifaciens	Bacteria	7.04
270	626934	Slackia piriformis	Bacteria	6.83
271	28026	Bifidobacterium pseudocatenulatum	Bacteria	6.83
272	1834205	Burkholderiales bacterium YL45	Bacteria	6.81
273	84024	Clostridium disporicum	Bacteria	6.68
274	1547597	Sanguibacteroides justesenii	Bacteria	6.61
275	938289	Levyella massiliensis	Bacteria	6.59
276	457397	Clostridium sp. 1_1_41A1FAA	Bacteria	6.54
277	1871013	Parabacteroides sp. Marseille-P3236	Bacteria	6.51
278	457402	Eubacterium sp. 3_1_31	Bacteria	6.49
279	1115692	Cannabis cryptic virus	Viruses	6.43
280	626933	Odoribacter laneus	Bacteria	6.39
281	12968	Blastocystis hominis	Eukaryota	6.38
282	1676614	Prevotella sp. 109	Bacteria	6.35
283	102148	Solobacterium moorei	Bacteria	6.32
284	342942	[Clostridium] glycyrrhizinilyticum	Bacteria	6.25
285	871325	Bacteroides faecichinchillae	Bacteria	6.24

286	47900	Garlic common latent virus	Viruses	6.21
287	585544	Bacteroides sp. D22	Bacteria	6.19
288	1602172	Prevotella sp. P5-125	Bacteria	6.14
289	1697795	Clostridia bacterium UC5.1-2H11	Bacteria	6.14
290	186772	Saccharomyces 20S RNA narnavirus	Viruses	6.10
291	1499681	Collinsella sp. MS5	Bacteria	6.10
292	76517	Campylobacter hominis	Bacteria	5.90
293	1284708	Tissierellia bacterium S7-1-4	Bacteria	5.88
294	1579343	Streptococcus sp. 263_SSPC	Bacteria	5.67
295	362693	Oryza sativa endornavirus	Viruses	5.65
296	1736	Eubacterium limosum	Bacteria	5.62
297	1078087	Parabacteroides sp. HGS0025	Bacteria	5.58
298	39483	Faecalitalea cylindroides	Bacteria	5.54
299	358743	[Clostridium] citroniae	Bacteria	5.44
300	1816678	Christensenella timonensis	Bacteria	5.40
301	658085	Lachnospiraceae bacterium 5_1_57FAA	Bacteria	5.36
302	665940	Clostridium sp. 7_3_54FAA	Bacteria	5.24
303	1406512	Candidatus Methanomassiliicoccus intestinalis	Archaea	5.16
304	944170	Blastocystis sp. subtype 4	Eukaryota	5.08
305	28125	Prevotella bivia	Bacteria	4.93
306	1778	Mycobacterium gordonae	Bacteria	4.90
307	1658112	Eubacterium sp. SB2	Bacteria	4.85
308	487173	Dialister succinatiphilus	Bacteria	4.70
309	1625	Lactobacillus sanfranciscensis	Bacteria	4.62
310	290052	Acetivibrio ethanolgignens	Bacteria	4.54
311	1304	Streptococcus salivarius	Bacteria	4.49
312	1261	Peptostreptococcus anaerobius	Bacteria	4.44
313	1391702	Tomato mottle mosaic virus	Viruses	4.43
314	33964	Leuconostoc citreum	Bacteria	4.38
315	43997	Catonella morbi	Bacteria	4.32
316	1519438	Eubacterium sp. ER2	Bacteria	4.30
317	272548	Actinomyces dentalis	Bacteria	4.23
318	28128	Prevotella corporis	Bacteria	4.18
319	327387	Tropical soda apple mosaic virus	Viruses	4.16
320	1761477	Tomato brown rugose fruit virus	Viruses	4.09
321	658656	Lachnospiraceae bacterium 6_1_37FAA	Bacteria	4.08
322	12175	Apple chlorotic leaf spot virus	Viruses	4.07
323	1841865	Mediterranea massiliensis	Bacteria	4.05
324	28123	Porphyromonas asaccharolytica	Bacteria	4.04
325	1871003	Tidjanibacter massiliensis	Bacteria	4.02
326	1671366	Ruminococcus sp. DSM 100440	Bacteria	4.00
327	80879	Curvibacter delicatus	Bacteria	3.97
328	936595	Lachnoanaerobaculum sp. OBRC5-5	Bacteria	3.97
329	12305	Cucumber mosaic virus	Viruses	3.95
330	658657	Erysipelotrichaceae bacterium 21_3	Bacteria	3.92
331	12242	Tobacco mosaic virus	Viruses	3.92
332	376805	Bacteroides salanitronis	Bacteria	3.91
333	1723384	Fenollaria timonensis	Bacteria	3.88

334	1871030	Merdibacter massiliensis	Bacteria	3.87
335	4932	Saccharomyces cerevisiae	Eukaryota	3.87
336	626935	Collinsella tanakaei	Bacteria	3.78
337	1501392	Coprobacter secundus	Bacteria	3.67
338	1255	Pediococcus pentosaceus	Bacteria	3.67
339	747056	Blueberry shock virus	Viruses	3.66
340	729	Haemophilus parainfluenzae	Bacteria	3.58
341	51330	Cucurbit yellow stunting disorder virus	Viruses	3.55
342	712411	Olsenella sp. oral taxon 807	Bacteria	3.54
343	1816694	Clostridium sp. Marseille-P2538	Bacteria	3.47
344	33763	Peanut mottle virus	Viruses	3.46
345	397865	Barnesiella viscericola	Bacteria	3.45
346	1197717	Cloacibacillus porcorum	Bacteria	3.44
347	1602168	Prevotella sp. P4-65	Bacteria	3.43
348	1261635	Roseburia sp. 831b	Bacteria	3.28
349	368735	Bell pepper mottle virus	Viruses	3.27
350	376804	Bacteroides barnesiae	Bacteria	3.27
351	1660	Actinomyces odontolyticus	Bacteria	3.27
352	1609975	Clostridium sp. FS41	Bacteria	3.26
353	580026	Enterorhabdus mucosicola	Bacteria	3.25
354	1686296	Gabonia massiliensis	Bacteria	3.25
355	1903262	Bacteroides sp. Marseille-P3108	Bacteria	3.25
356	1261634	Roseburia sp. 499	Bacteria	3.24
357	47246	[Clostridium] viride	Bacteria	3.20
358	28130	Prevotella disiens	Bacteria	3.18
359	386414	Prevotella timonensis	Bacteria	3.18
360	44742	Desulfovibrio fairfieldensis	Bacteria	3.16
361	1265	Ruminococcus flavefaciens	Bacteria	3.10
362	1744	Propionibacterium freudenreichii	Bacteria	3.07
363	1584	Lactobacillus delbrueckii	Bacteria	3.07
364	157777	Pea streak virus	Viruses	3.07
365	1491	Clostridium botulinum	Bacteria	3.06
366	1322	Blautia hansenii	Bacteria	3.03
367	1907658	Bacteroides sp. Marseille-P3208T	Bacteria	2.97
368	480391	Pediococcus argentinicus	Bacteria	2.97
369	1232460	Clostridiales bacterium VE202-28	Bacteria	2.95
370	311413	Lettuce big-vein associated virus	Viruses	2.95
371	712124	Actinomyces sp. oral taxon 448	Bacteria	2.91
372	39482	[Eubacterium] contortum	Bacteria	2.89
373	665938	Bacteroides sp. 2_1_56FAA	Bacteria	2.89
374	665937	Anaerostipes sp. 3_2_56FAA	Bacteria	2.87
375	1583	Weissella confusa	Bacteria	2.87
376	1232447	Clostridiales bacterium VE202-09	Bacteria	2.86
377	2317	Methanosphaera stadtmanae	Archaea	2.79
378	28139	Rikenella microfusus	Bacteria	2.78
379	508460	Cloacibacillus evryensis	Bacteria	2.76
380	1870994	Urmitella timonensis	Bacteria	2.75
381	457422	Erysipelotrichaceae bacterium 2_2_44A	Bacteria	2.73

382	147206	Collinsella stercoris	Bacteria	2.71
383	1232442	Clostridiales bacterium VE202-06	Bacteria	2.71
384	658083	Lachnospiraceae bacterium 6_1_63FAA	Bacteria	2.71
385	281920	Porphyromonas uenonis	Bacteria	2.68
386	469597	Coprobacillus sp. 8_2_54BFAA	Bacteria	2.67
387	1816677	Butyricimonas sp. Marseille-P2440	Bacteria	2.60
388	658088	Lachnospiraceae bacterium 9_1_43BFAA	Bacteria	2.53
389	35787	Lactobacillus pontis	Bacteria	2.52
390	28347	Apple stem grooving virus	Viruses	2.52
391	665941	Coprobacillus sp. 3_3_56FAA	Bacteria	2.50
392	944168	Blastocystis sp. subtype 3	Eukaryota	2.49
393	12319	Apple mosaic virus	Viruses	2.46
394	491921	Megamonas rupellensis	Bacteria	2.43
395	1046403	Brassica yellows virus	Viruses	2.41
396	1472761	Enorma massiliensis	Bacteria	2.39
397	1574264	Akkermansia sp. KLE1797	Bacteria	2.37
398	1602171	Prevotella sp. P5-119	Bacteria	2.37
399	1235797	Oscillibacter sp. 1-3	Bacteria	2.29
400	1623	Lactobacillus ruminis	Bacteria	2.28
401	1852383	Ruminococcaceae bacterium Marseille-P2935	Bacteria	2.28
402	11987	Melon necrotic spot virus	Viruses	2.28
403	52227	Prevotella dentalis	Bacteria	2.26
404	1599	Lactobacillus sakei	Bacteria	2.26
405	501496	Porphyromonas bennonis	Bacteria	2.22
406	290053	Bacteroides helcogenes	Bacteria	2.15
407	2702	Gardnerella vaginalis	Bacteria	2.13
408	1739394	Porphyromonas sp. HMSC065F10	Bacteria	2.13
409	944036	Blastocystis sp. subtype 1	Eukaryota	2.12
410	28127	Prevotella buccalis	Bacteria	2.10
411	1796616	Blautia sp. YL58	Bacteria	2.07
412	187327	Acidaminococcus intestini	Bacteria	2.04
413	469587	Bacteroides sp. 2_1_16	Bacteria	2.04
414	461393	Actinomyces massiliensis	Bacteria	2.03
415	12169	Potato virus S	Viruses	2.02
416	91753	Cucurbit aphid-borne yellows virus	Viruses	2.02
417	502558	Eggerthella sp. YY7918	Bacteria	2.02
418	671266	Enterorhabdus caecimuris	Bacteria	2.01
419	1230734	Clostridiales bacterium S5-A14a	Bacteria	2.01
420	1658109	Candidatus Stoquefichus sp. SB1	Bacteria	2.01
421	5722	Trichomonas vaginalis	Eukaryota	2.00
422	419015	Alloscardovia omnicolens	Bacteria	1.99
423	665942	Desulfovibrio sp. 6_1_46AFAA	Bacteria	1.98
424	442302	Porcine picobirnavirus	Viruses	1.96
425	1585974	Beduini massiliensis	Bacteria	1.96
426	35350	Apple stem pitting virus	Viruses	1.95
427	310300	Bacteroides pyogenes	Bacteria	1.94
428	1590	Lactobacillus plantarum	Bacteria	1.92
429	131111	Actinomyces turicensis	Bacteria	1.91

430	1502	Clostridium perfringens	Bacteria	1.90
431	187326	Megasphaera micronuciformis	Bacteria	1.89
432	665943	Eggerthella sp. 1_3_56FAA	Bacteria	1.88
433	243563	Strawberry necrotic shock virus	Viruses	1.84
434	827	Campylobacter ureolyticus	Bacteria	1.83
435	1603888	Megasphaera sp. MJR8396C	Bacteria	1.83
436	1564113	Sphingomonas sp. Ant H11	Bacteria	1.82
437	1323	Faecalicoccus pleomorphus	Bacteria	1.82
438	1903261	Desulfovibrio sp. Marseille-P3199	Bacteria	1.79
439	1611875	RNA	Viruses	1.79
440	509923	Beet cryptic virus 1	Viruses	1.79
441	1602169	Prevotella sp. P4-76	Bacteria	1.78
442	1632013	Drancourtella massiliensis	Bacteria	1.78
443	1871016	Collinsella sp. Marseille-P3245	Bacteria	1.77
444	187979	Mitsuokella jalaludinii	Bacteria	1.77
445	252598	Saccharomyces sp. 'boulardii'	Eukaryota	1.76
446	706435	Capnocytophaga sp. oral taxon 329	Bacteria	1.75
447	1078090	Coprococcus sp. HPP0074	Bacteria	1.74
448	1907654	Collinsella sp. Marseille-P3296T	Bacteria	1.67
449	1871022	Libanicoccus massiliensis	Bacteria	1.67
450	1720313	Bittarella massiliensis	Bacteria	1.66
451	1685	Bifidobacterium breve	Bacteria	1.66
452	220618	Cucumber Bulgarian virus	Viruses	1.63
453	39950	Dialister pneumosintes	Bacteria	1.62
454	378833	Sowbane mosaic virus	Viruses	1.58
455	1232452	Clostridiales bacterium VE202-14	Bacteria	1.57
456	742723	Lachnospiraceae bacterium 2_1_46FAA	Bacteria	1.57
457	1244	Leuconostoc gelidum	Bacteria	1.57
458	518643	Bifidobacterium mongoliense	Bacteria	1.56
459	28135	Prevotella oris	Bacteria	1.56
460	1834196	Lachnoclostridium sp. YL32	Bacteria	1.54
461	1323529	Dill cryptic virus 2	Viruses	1.54
462	1499684	Clostridium sp. CL-2	Bacteria	1.54
463	28038	Lactobacillus curvatus	Bacteria	1.53
464	1778580	Nectarine virus M	Viruses	1.52
465	755172	Peptoniphilus coxii	Bacteria	1.52
466	33760	Prune dwarf virus	Viruses	1.52
467	658665	Dorea sp. D27	Bacteria	1.52
468	552396	Erysipelotrichaceae bacterium 5_2_54FAA	Bacteria	1.51
469	354328	Bell pepper endornavirus	Viruses	1.50
470	1602170	Prevotella sp. P5-60	Bacteria	1.49
471	1714570	Blueberry shoestring virus	Viruses	1.48
472	29466	Veillonella parvula	Bacteria	1.47
473	1111120	Acidaminococcus sp. BV3L6	Bacteria	1.47
474	165432	Cucumber leaf spot virus	Viruses	1.46
475	1598	Lactobacillus reuteri	Bacteria	1.42
476	1841857	Culturomica massiliensis	Bacteria	1.40
477	47770	Lactobacillus crispatus	Bacteria	1.39

478	76122	Alloprevotella tannerae	Bacteria	1.38
479	12321	Alfalfa mosaic virus	Viruses	1.37
480	437897	Megamonas funiformis	Bacteria	1.35
481	666	Vibrio cholerae	Bacteria	1.31
482	1613	Lactobacillus fermentum	Bacteria	1.29
483	1739517	Bacteroides sp. HMSC073E02	Bacteria	1.29
484	1105029	Actinomyces sp. ICM39	Bacteria	1.29
485	1917878	Prevotella ihumii	Bacteria	1.28
486	12302	Brome mosaic virus	Viruses	1.28
487	67761	Cowpea mild mottle virus	Viruses	1.27
488	1284775	Prevotella sp. S7-1-8	Bacteria	1.27
489	1470354	Candidatus Soleaferrea massiliensis	Bacteria	1.26
490	31741	Wheat streak mosaic virus	Viruses	1.26
491	1581179	Clostridium sp. HMSC19A11	Bacteria	1.26
492	1078091	Coprococcus sp. HPP0048	Bacteria	1.25
493	1739408	Streptococcus sp. HMSC072G04	Bacteria	1.24
494	105612	Lactobacillus algidus	Bacteria	1.24
495	45634	Streptococcus cristatus	Bacteria	1.24
496	1338	Streptococcus intermedius	Bacteria	1.24
497	544581	Actinomyces johnsonii	Bacteria	1.23
498	341694	Peptostreptococcus stomatis	Bacteria	1.21
499	1042156	Clostridium sp. SY8519	Bacteria	1.21
500	850	Fusobacterium mortiferum	Bacteria	1.20
501	861	Fusobacterium ulcerans	Bacteria	1.20
502	658082	Lachnospiraceae bacterium 2_1_58FAA	Bacteria	1.20
503	1739304	Anaerosphaera sp. HMSC064C01	Bacteria	1.19
504	1232428	Megasphaera massiliensis	Bacteria	1.19
505	1870997	Mogibacterium sp. Marseille-P3115	Bacteria	1.19
506	1264	Ruminococcus albus	Bacteria	1.18
507	52769	Actinomyces gerencseriae	Bacteria	1.17
508	1697788	Clostridia bacterium UC5.1-2H6	Bacteria	1.17
509	1261636	Anaerostipes sp. 494a	Bacteria	1.16
510	28132	Prevotella melaninogenica	Bacteria	1.15
511	28126	Prevotella buccae	Bacteria	1.15
512	147802	Lactobacillus iners	Bacteria	1.15
513	322095	Porphyromonas somerae	Bacteria	1.14
514	1351	Enterococcus faecalis	Bacteria	1.13
515	1398	Bacillus coagulans	Bacteria	1.10
516	52226	Mitsuokella multacida	Bacteria	1.10
517	230143	Scardovia wiggsiae	Bacteria	1.10
518	1851429	Christensenella sp. AF73-05CM02	Bacteria	1.09
519	1305	Streptococcus sanguinis	Bacteria	1.07
520	31722	Blueberry scorch virus	Viruses	1.07
521	242750	Prevotella bergensis	Bacteria	1.07
522	137838	Clostridium neonatale	Bacteria	1.06
523	12470	Lucerne transient streak virus	Viruses	1.06
524	1933298	Tomato spotted wilt tospovirus	Viruses	1.05
525	180164	Blautia schinkii	Bacteria	1.04

526	1408895	Dill cryptic virus 1	Viruses	1.04
527	1549	[Clostridium] sporosphaeroides	Bacteria	1.03
528	77095	Prevotella bryantii	Bacteria	1.02
529	55951	Grapevine leafroll-associated virus 3	Viruses	1.00
530	198589	Beet western yellows ST9 associated virus	Viruses	1.00
531	1624	Lactobacillus salivarius	Bacteria	0.99
532	1280685	Butyrivibrio sp. NC3005	Bacteria	0.99
533	1364	Lactococcus piscium	Bacteria	0.98
534	1834207	Erysipelotrichaceae bacterium I46	Bacteria	0.97
535	457398	Desulfovibrio sp. 3_1_syn3	Bacteria	0.97
536	29391	Gemella morbillorum	Bacteria	0.96
537	1307	Streptococcus suis	Bacteria	0.95
538	1414720	Clostridium saudiense	Bacteria	0.95
539	29363	Clostridium paraputrificum	Bacteria	0.94
540	884684	Mageeibacillus indolicus	Bacteria	0.94
541	556261	Clostridium sp. D5	Bacteria	0.93
542	907	Megasphaera elsdenii	Bacteria	0.91
543	1750	Pseudopropionibacterium propionicum	Bacteria	0.89
544	1329795	Clostridiaceae bacterium MS3	Bacteria	0.89
545	228604	Prevotella salivae	Bacteria	0.89
546	287	Pseudomonas aeruginosa	Bacteria	0.89
547	329	Ralstonia pickettii	Bacteria	0.88
548	309120	Dialister micraerophilus	Bacteria	0.86
549	1528099	Lawsonella clevelandensis	Bacteria	0.86
550	146500	Watermelon mosaic virus	Viruses	0.86
551	1111134	Peptoniphilus sp. BV3C26	Bacteria	0.85
552	82135	Atopobium vaginae	Bacteria	0.84
553	1267	Clostridium ventriculi	Bacteria	0.83
554	1235792	Lachnospiraceae bacterium M18-1	Bacteria	0.82
555	78345	Bifidobacterium merycicum	Bacteria	0.82
556	196400	Grapevine rupestris stem pitting-associated virus	Viruses	0.82
557	11983	Norwalk virus	Viruses	0.80
558	837	Porphyromonas gingivalis	Bacteria	0.79
559	905	Acidaminococcus fermentans	Bacteria	0.78
560	133926	Olsenella uli	Bacteria	0.78
561	999425	Streptococcus sp. F0442	Bacteria	0.77
562	1579	Lactobacillus acidophilus	Bacteria	0.76
563	1504822	bacterium OL-1	Bacteria	0.76
564	37734	Enterococcus casseliflavus	Bacteria	0.76
565	1797112	Olsenella sp. kh2p3	Bacteria	0.76
566	12162	Citrus tristeza virus	Viruses	0.76
567	12042	Beet western yellows virus	Viruses	0.75
568	1254	Pediococcus acidilactici	Bacteria	0.75
569	1378168	Firmicutes bacterium ASF500	Bacteria	0.75
570	46681	Entamoeba dispar	Eukaryota	0.74
571	877411	Ruminococcus sp. NK3A76	Bacteria	0.74
572	1232448	Clostridiales bacterium VE202-07	Bacteria	0.73
573	70177	Grapevine leafroll-associated virus 4	Viruses	0.72

574	1564903	Strawberry polerovirus 1	Viruses	0.72
575	12282	Tobacco ringspot virus	Viruses	0.72
576	425279	Rehmannia mosaic virus	Viruses	0.71
577	199	Campylobacter concisus	Bacteria	0.70
578	156456	Anaeroglobus geminatus	Bacteria	0.70
579	1078480	Haemophilus sputorum	Bacteria	0.70
580	732242	Bacteroides paurosaccharolyticus	Bacteria	0.70
581	437898	Sutterella parvirubra	Bacteria	0.69
582	46125	Abiotrophia defectiva	Bacteria	0.69
583	42680	Spinach latent virus	Viruses	0.69
584	53442	Eubacterium callanderi	Bacteria	0.69
585	1110546	Veillonella tobetsuensis	Bacteria	0.68
586	12451	Raspberry bushy dwarf virus	Viruses	0.66
587	1776081	Megasphaera sp. DISK 18	Bacteria	0.65
588	1310	Streptococcus sobrinus	Bacteria	0.65
589	2748	Carnobacterium divergens	Bacteria	0.65
590	1673717	Anaeromassilibacillus senegalensis	Bacteria	0.65
591	556499	Propionibacterium acidifaciens	Bacteria	0.65
592	1203555	Acidaminococcus sp. HPA0509	Bacteria	0.64
593	12844	Sweet potato feathery mottle virus	Viruses	0.64
594	515414	Prevotella falsenii	Bacteria	0.64
595	67754	Tomato chlorosis virus	Viruses	0.63
596	1588755	Parvimonas sp. KA00067	Bacteria	0.63
597	60920	Sanguibacter keddieii	Bacteria	0.62
598	51123	[Eubacterium] saphenum	Bacteria	0.62
599	228578	Youcai mosaic virus	Viruses	0.62
600	1655	Actinomyces naeslundii	Bacteria	0.62
601	419005	Prevotella amnii	Bacteria	0.61
602	12280	Tomato ringspot virus	Viruses	0.60
603	187101	Sneathia amnii	Bacteria	0.60
604	184870	Varibaculum cambriense	Bacteria	0.59
605	39029	Megasphaera cerevisiae	Bacteria	0.59
606	1577792	Terrisporobacter othiniensis	Bacteria	0.59
607	1173061	Geotrichum candidum	Eukaryota	0.59
608	155892	Caulobacter vibrioides	Bacteria	0.58
609	1261637	Anaerostipes sp. 992a	Bacteria	0.58
610	12317	Tobacco streak virus	Viruses	0.57
611	61592	Corynebacterium durum	Bacteria	0.57
612	712117	Actinomyces sp. oral taxon 170	Bacteria	0.57
613	89152	[Clostridium] hiranonis	Bacteria	0.56
614	1871336	Criibacterium bergeronii	Bacteria	0.56
615	72149	Aichivirus A	Viruses	0.55
616	12433	Garlic virus A	Viruses	0.55
617	12187	Strawberry mild yellow edge virus	Viruses	0.55
618	1263547	Oscillibacter ruminantium	Bacteria	0.55
619	477666	Bacteroides graminisolvens	Bacteria	0.55
620	712118	Actinomyces sp. oral taxon 172	Bacteria	0.55
621	1720195	Gabonibacter massiliensis	Bacteria	0.54

622	1605	Lactobacillus animalis	Bacteria	0.53
623	33032	Anaerococcus lactolyticus	Bacteria	0.53
624	12216	Potato virus Y	Viruses	0.52
625	1105030	Actinomyces sp. ICM58	Bacteria	0.52
626	1393034	Atopobium deltae	Bacteria	0.51
627	1532180	Penicillium roqueforti ssRNA mycovirus 1	Viruses	0.50
628	1697790	Clostridia bacterium UC5.1-1C12	Bacteria	0.50
629	39441	Methanobrevibacter arboriphilus	Archaea	0.50
630	1051631	Streptococcus phage YMC-2011	Viruses	0.50
631	1805478	Olsenella sp. Marseille-P2300	Bacteria	0.49
632	1852386	Olsenella sp. Marseille-P2912	Bacteria	0.49
633	1111135	Coriobacteriaceae bacterium BV3Ac1	Bacteria	0.49
634	112227	Cactus virus X	Viruses	0.48
635	556270	Coprobacillus sp. D7	Bacteria	0.48
636	877414	Clostridiales bacterium NK3B98	Bacteria	0.48
637	1697792	Clostridia bacterium UC5.1-2F7	Bacteria	0.48
638	1937817	Spinach cryptic virus 1	Viruses	0.48
639	35518	[Eubacterium] nodatum	Bacteria	0.48
640	1739529	Porphyromonas sp. HMSC077F02	Bacteria	0.48
641	1561	Clostridium baratii	Bacteria	0.48
642	1232446	Clostridiales bacterium VE202-18	Bacteria	0.47
643	1531429	Coriobacteriaceae bacterium 68-1-3	Bacteria	0.47
644	237576	Oribacterium sinus	Bacteria	0.47
645	642478	Lettuce chlorosis virus	Viruses	0.47
646	1852374	Ezakiella massiliensis	Bacteria	0.46
647	181487	Actinomyces cardiffensis	Bacteria	0.46
648	824	Campylobacter gracilis	Bacteria	0.46
649	66200	Carrot red leaf virus	Viruses	0.46
650	39777	Veillonella atypica	Bacteria	0.45
651	12263	Squash mosaic virus	Viruses	0.45
652	1673723	Murdochiella massiliensis	Bacteria	0.45
653	580254	Raphanus sativus cryptic virus 3	Viruses	0.45
654	1630	Kandleria vitulina	Bacteria	0.45
655	12205	Papaya ringspot virus	Viruses	0.44
656	290055	[Eubacterium] fissicatena	Bacteria	0.44
657	1392494	Lachnospiraceae bacterium AC2012	Bacteria	0.43
658	1470350	Candidatus Stoquefichus massiliensis	Bacteria	0.42
659	54005	Peptoniphilus harei	Bacteria	0.42
660	431317	Triticum mosaic virus	Viruses	0.42
661	1226323	Oscillibacter sp. KLE 1745	Bacteria	0.42
662	544580	Actinomyces oris	Bacteria	0.42
663	1392836	Lachnospiraceae bacterium TWA4	Bacteria	0.42
664	727	Haemophilus influenzae	Bacteria	0.42
665	1232445	Clostridiales bacterium VE202-16	Bacteria	0.41
666	1601	Lactobacillus agilis	Bacteria	0.41
667	1595998	Human smacovirus 1	Viruses	0.41
668	28037	Streptococcus mitis	Bacteria	0.41
669	1489836	Atlantic salmon calicivirus	Viruses	0.40

670	52229	[Hallella] seregens	Bacteria	0.40
671	712991	Lachnospiraceae bacterium oral taxon 500	Bacteria	0.40
672	10641	Cauliflower mosaic virus	Viruses	0.40
673	1852375	Acidaminococcus massiliensis	Bacteria	0.39
674	1465757	Peptoniphilus senegalensis	Bacteria	0.39
675	5007	Brettanomyces bruxellensis	Eukaryota	0.39
676	1479	Bacillus smithii	Bacteria	0.39
677	76875	Broad bean wilt virus 2	Viruses	0.39
678	1689	Bifidobacterium dentium	Bacteria	0.39
679	29322	[Eubacterium] cellulosolvens	Bacteria	0.39
680	1776177	Cucumis melo endornavirus	Viruses	0.39
681	322505	Sharpea azabuensis	Bacteria	0.38
682	736	Haemophilus paraphrohaemolyticus	Bacteria	0.38
683	457396	Clostridium sp. 7_2_43FAA	Bacteria	0.37
684	1683	Bifidobacterium angulatum	Bacteria	0.37
685	103722	Grapevine fleck virus	Viruses	0.37
686	1382	Atopobium parvulum	Bacteria	0.36
687	1581061	Abiotrophia sp. HMSC24B09	Bacteria	0.36
688	739	Aggregatibacter segnis	Bacteria	0.36
689	467210	Lachnoanaerobaculum saburreum	Bacteria	0.35
690	859	Fusobacterium necrophorum	Bacteria	0.35
691	95342	Sapporo virus	Viruses	0.35
692	210	Helicobacter pylori	Bacteria	0.35
693	397544	Squash vein yellowing virus	Viruses	0.35
694	56774	[Eubacterium] infirmum	Bacteria	0.35
695	84163	Cryptobacterium curtum	Bacteria	0.35
696	1499683	Clostridium sp. CL-6	Bacteria	0.35
697	1676617	Ralstonia sp. MD27	Bacteria	0.35
698	310514	Prevotella multisaccharivorax	Bacteria	0.34
699	12274	Grapevine fanleaf virus	Viruses	0.34
700	908340	Clostridium sp. HGF2	Bacteria	0.34
701	35517	[Eubacterium] brachy	Bacteria	0.34
702	638849	Pyramidobacter piscolens	Bacteria	0.33
703	28051	Lachnospira multipara	Bacteria	0.33
704	145856	Human picobirnavirus	Viruses	0.33
705	113107	Streptococcus australis	Bacteria	0.33
706	29360	Cellulosilyticum lentocellum	Bacteria	0.32
707	47671	Lautropia mirabilis	Bacteria	0.32
708	45254	Dysgonomonas capnocytophagoides	Bacteria	0.31
709	12056	Tobacco necrosis virus D	Viruses	0.31
710	1219626	Peptostreptococcus sp. MV1	Bacteria	0.31
711	1284680	Actinomyces sp. S6-Spd3	Bacteria	0.31
712	39046	Cassava common mosaic virus	Viruses	0.31
713	209529	Aphid lethal paralysis virus	Viruses	0.31
714	138595	Olsenella profusa	Bacteria	0.31
715	137591	Weissella cibaria	Bacteria	0.31
716	33034	Anaerococcus prevotii	Bacteria	0.31
717	12615	Cherry leaf roll virus	Viruses	0.31

718	796942	Stomatobaculum longum	Bacteria	0.31
719	1366	Lactococcus raffinolactis	Bacteria	0.31
720	1776390	Peptoniphilus sp. KHD5	Bacteria	0.30
721	163665	Dysgonomonas mossii	Bacteria	0.30
722	97478	Lactobacillus mucosae	Bacteria	0.30
723	12196	Bean common mosaic virus	Viruses	0.30
724	231049	Lactobacillus rossiae	Bacteria	0.30
725	78258	Parascardovia denticolens	Bacteria	0.29
726	626938	Succinatimonas hippei	Bacteria	0.29
727	1871012	Mobilibacterium timonense	Bacteria	0.29
728	32625	Mushroom bacilliform virus	Viruses	0.29
729	1686	Bifidobacterium catenulatum	Bacteria	0.29
730	193118	Lucerne transient streak virus satellite RNA	Viruses	0.29
731	1739305	Peptoniphilus sp. HMSC062D09	Bacteria	0.28
732	43768	Corynebacterium matruchotii	Bacteria	0.28
733	558690	Cucurbit chlorotic yellows virus	Viruses	0.28
734	1252	Leuconostoc carnosum	Bacteria	0.28
735	1689303	Lagierella massiliensis	Bacteria	0.28
736	1379	Gemella haemolysans	Bacteria	0.27
737	563031	Prevotella sp. C561	Bacteria	0.27
738	1588754	Veillonellaceae bacterium DNF00626	Bacteria	0.27
739	12436	Cucumber mosaic virus satellite RNA	Viruses	0.27
740	82688	Lactobacillus nagelii	Bacteria	0.27
741	930881	Sweet potato virus C	Viruses	0.27
742	36841	Terrisporobacter glycolicus	Bacteria	0.26
743	67962	Carrot red leaf luteovirus associated RNA	Viruses	0.26
744	42004	Leek yellow stripe virus	Viruses	0.26
745	131083	Turnip yellows virus	Viruses	0.26
746	1871017	Peptoniphilus urinimassiliensis	Bacteria	0.26
747	1596	Lactobacillus gasseri	Bacteria	0.26
748	54062	Pediococcus parvulus	Bacteria	0.26
749	198599	Saccharomyces 23S RNA narnavirus	Viruses	0.26
750	42478	Saccharomyces cerevisiae virus L-BC (La)	Viruses	0.26
751	40543	Sneathia sanguinegens	Bacteria	0.25
752	1449897	Uncultured phage WW-nAnB strain 3	Viruses	0.25
753	1852387	Streptococcus timonensis	Bacteria	0.25
754	78343	Bifidobacterium boum	Bacteria	0.25
755	1169032	Wasabi mottle virus	Viruses	0.25
756	29833	Hanseniaspora uvarum	Eukaryota	0.25
757	1302	Streptococcus gordonii	Bacteria	0.25
758	158847	Megamonas hypermegale	Bacteria	0.25
759	1659	Actinomyces israelii	Bacteria	0.25
760	1301220	Citrus vein enation virus	Viruses	0.25
761	150284	Garlic virus X	Viruses	0.25
762	851	Fusobacterium nucleatum	Bacteria	0.25
763	1502943	Veillonella seminalis	Bacteria	0.25
764	42882	Cherry virus A	Viruses	0.25
765	450749	Veillonella sp. 6_1_27	Bacteria	0.25

766	12295	Tobacco rattle virus	Viruses	0.25
767	180332	Robinsoniella peoriensis	Bacteria	0.25
768	244362	Ruminococcus sp. YE71	Bacteria	0.25
769	1805477	Clostridium sp. Marseille-P299	Bacteria	0.24
770	12232	Zucchini yellow mosaic virus	Viruses	0.24
771	109790	Lactobacillus jensenii	Bacteria	0.24
772	12041	Bean leafroll virus	Viruses	0.24
773	180903	Pineapple mealybug wilt-associated virus 1	Viruses	0.24
774	12145	Tomato bushy stunt virus	Viruses	0.24
775	1633	Lactobacillus vaginalis	Bacteria	0.24
776	45661	Banana bract mosaic virus	Viruses	0.23
777	83771	Succinivibrio dextrinosolvens	Bacteria	0.23
778	167161	Strawberry mottle virus	Viruses	0.23
779	114652	Streptococcus orisratti	Bacteria	0.23
780	598660	Corynebacterium pyruviciproducens	Bacteria	0.23
781	1415630	Pseudomonas sp. TKP	Bacteria	0.23
782	253700	Schlumbergera virus X	Viruses	0.22
783	1463935	Streptomyces sp. NRRL WC-3744	Bacteria	0.22
784	165096	Weissella koreensis	Bacteria	0.22
785	537288	Megasphaera sp. DJF_B143	Bacteria	0.22
786	735	Haemophilus parahaemolyticus	Bacteria	0.22
787	397288	Lachnospiraceae bacterium 3-1	Bacteria	0.22
788	1074044	uncultured phage WW-nAnB	Viruses	0.22
789	646413	Streptococcus phage 5093	Viruses	0.21
790	1501391	Alistipes inops	Bacteria	0.21
791	1492	Clostridium butyricum	Bacteria	0.21
792	11008	Saccharomyces cerevisiae virus L-A	Viruses	0.21
793	849	Fusobacterium gonidiaformans	Bacteria	0.21
794	1739251	Fusobacterium sp. HMSC073F01	Bacteria	0.21
795	1251	Leuconostoc fallax	Bacteria	0.21
796	563191	Acidaminococcus sp. D21	Bacteria	0.21
797	1852372	Varibaculum sp. Marseille-P2802	Bacteria	0.20
798	1776381	Olsenella sp. KHD7	Bacteria	0.20
799	2051	Mobiluncus curtisii	Bacteria	0.20
800	1653434	Sellimonas intestinalis	Bacteria	0.20
801	483	Neisseria cinerea	Bacteria	0.20
802	428712	Jonquetella anthropi	Bacteria	0.20
803	675077	Plum bark necrosis stem pitting-associated virus	Viruses	0.20
804	2130	Ureaplasma urealyticum	Bacteria	0.20
805	938288	Fenollaria massiliensis	Bacteria	0.20
806	416586	Selenomonas bovis	Bacteria	0.20
807	304207	Lactobacillus harbinensis	Bacteria	0.20
808	1216932	Clostridium bornimense	Bacteria	0.20
809	196375	Beet black scorch virus	Viruses	0.19
810	1206566	Blueberry virus A	Viruses	0.19
811	28136	Prevotella oulorum	Bacteria	0.19
812	1658108	Niameybacter massiliensis	Bacteria	0.19
813	1720204	Collinsella ihuae	Bacteria	0.19

814	712122	Actinomyces sp. oral taxon 414	Bacteria	0.18
815	1505	Paeniclostridium sordellii	Bacteria	0.18
816	1497955	Clostridiales bacterium KA00274	Bacteria	0.18
817	78448	Bifidobacterium pullorum	Bacteria	0.18
818	1291540	Candidatus Methanomethylophilus alvus	Archaea	0.18
819	1871014	Arcanobacterium urinimassiliense	Bacteria	0.18
820	1280674	Prevotella sp. AGR2160	Bacteria	0.18
821	1580	Lactobacillus brevis	Bacteria	0.18
822	1871002	Acidaminococcus timonensis	Bacteria	0.18
823	174709	Allobaculum stercoricanis	Bacteria	0.18
824	1347366	Clostridium sp. ND2	Bacteria	0.18
825	1303	Streptococcus oralis	Bacteria	0.18
826	1720317	Porphyromonadaceae bacterium FC4	Bacteria	0.18
827	936588	Veillonella sp. ACP1	Bacteria	0.18
828	1739406	Actinomyces sp. HMSC035G02	Bacteria	0.17
829	296	Pseudomonas fragi	Bacteria	0.17
830	1852362	Bacteroides ihuae	Bacteria	0.17
831	64003	Grapevine leafroll-associated virus 2	Viruses	0.17
832	1871033	Olsenella sp. Marseille-P3197	Bacteria	0.17
833	157688	Leptotrichia hofstadii	Bacteria	0.17
834	1659298	Nectarine stem pitting-associated virus	Viruses	0.17
835	1715051	Streptococcus sp. HMSC068F04	Bacteria	0.17
836	29272	Turnip vein-clearing virus	Viruses	0.17
837	1805470	Clostridium sp. Marseille-P2414	Bacteria	0.16
838	1655645	Parabacteroides phage YZ-2015b	Viruses	0.16
839	305	Ralstonia solanacearum	Bacteria	0.16
840	66228	Actinomyces europaeus	Bacteria	0.16
841	139208	Isoptericola variabilis	Bacteria	0.16
842	1497953	Bacteroidales bacterium KA00251	Bacteria	0.16
843	156974	Dysgonomonas gadei	Bacteria	0.16
844	47903	Strawberry vein banding virus	Viruses	0.16
845	852	Fusobacterium perfoetens	Bacteria	0.16
846	5741	Giardia intestinalis	Eukaryota	0.16
847	28133	Prevotella nigrescens	Bacteria	0.16
848	46619	Sweet potato virus G	Viruses	0.16
849	103724	Grapevine asteroid mosaic-associated virus	Viruses	0.16
850	1111454	Megasphaera sp. BV3C16-1	Bacteria	0.15
851	1472765	Peptoniphilus obesi	Bacteria	0.15
852	104263	Hop latent virus	Viruses	0.15
853	204	Campylobacter showae	Bacteria	0.15
854	1032506	Prevotella sp. MSX73	Bacteria	0.15
855	1334	Streptococcus dysgalactiae	Bacteria	0.15
856	1838287	Gammaproteobacteria bacterium 2W06	Bacteria	0.15
857	1720315	Eggerthellaceae bacterium AT8	Bacteria	0.15
858	1588753	Coriobacteriales bacterium DNF00809	Bacteria	0.15
859	1046402	Potato virus H	Viruses	0.15
860	457416	Veillonella sp. 3_1_44	Bacteria	0.15
861	1739522	Haemophilus sp. HMSC068C11	Bacteria	0.15

862	87541	Aerococcus christensenii	Bacteria	0.15
863	1343	Streptococcus vestibularis	Bacteria	0.15
864	1581080	Streptococcus sp. HMSC10E12	Bacteria	0.15
865	652706	Oribacterium sp. oral taxon 078	Bacteria	0.15
866	1203593	Veillonella sp. HPA0037	Bacteria	0.15
867	143361	Filifactor alocis	Bacteria	0.15
868	1679444	Akkermansia glycaniphila	Bacteria	0.15
869	1871023	Rikenella sp. Marseille-P3215	Bacteria	0.15
870	2098	Mycoplasma hominis	Bacteria	0.15
871	44008	Enterococcus cecorum	Bacteria	0.15
872	197614	Streptococcus pasteurianus	Bacteria	0.15
873	1410621	Lachnospiraceae bacterium AD3010	Bacteria	0.15
874	867080	Paenibacillus sp. IHB B 3415	Bacteria	0.15
875	1311	Streptococcus agalactiae	Bacteria	0.15
876	12430	Garlic virus D	Viruses	0.15
877	548908	Fig fleck-associated virus	Viruses	0.15
878	732	Aggregatibacter aphrophilus	Bacteria	0.15
879	12154	Turnip yellow mosaic virus	Viruses	0.15
880	164393	Lactobacillus fuchuensis	Bacteria	0.15
881	856	Fusobacterium varium	Bacteria	0.14
882	2096	Mycoplasma gallisepticum	Bacteria	0.14
883	267818	Lactobacillus kefiranofaciens	Bacteria	0.14
884	43769	Corynebacterium propinquum	Bacteria	0.14
885	5341	Agaricus bisporus	Eukaryota	0.14
886	66219	Lachnoclostridium phytofermentans	Bacteria	0.14
887	315405	Streptococcus gallolyticus	Bacteria	0.14
888	1739525	Peptoniphilus sp. HMSC075B08	Bacteria	0.14
889	1161409	Bifidobacterium sp. MSTE12	Bacteria	0.14
890	114922	Carrot thin leaf virus	Viruses	0.14
891	404196	Blackberry yellow vein-associated virus	Viruses	0.13
892	1943580	Pyramidobacter sp. C12-8	Bacteria	0.13
893	1449896	Uncultured phage WW-nAnB strain 2	Viruses	0.13
894	589436	Prevotella fusca	Bacteria	0.13
895	28080	Campylobacter upsaliensis	Bacteria	0.13
896	1501329	Oribacterium parvum	Bacteria	0.13
897	168135	Apium virus Y	Viruses	0.13
898	213	Helicobacter cinaedi	Bacteria	0.13
899	469588	Bacteroides sp. 2_1_22	Bacteria	0.13
900	180311	Hespellia stercorisuis	Bacteria	0.13
901	193121	Pea enation mosaic virus-1	Viruses	0.13
902	33036	Anaerococcus tetradius	Bacteria	0.13
903	863372	Herbaspirillum huttiense	Bacteria	0.13
904	1579342	Streptococcus sp. 343_SSPC	Bacteria	0.13
905	267135	Porphyrobacter donghaensis	Bacteria	0.13
906	1123754	Rattail cactus necrosis-associated virus	Viruses	0.12
907	1547	Erysipelatoclostridium ramosum	Bacteria	0.12
908	81424	[Clostridium] methoxybenzovorans	Bacteria	0.12
909	1495144	methanogenic archaeon ISO4-H5	Archaea	0.12

910	502393	Gemella asaccharolytica	Bacteria	0.12
911	147711	Rhinovirus A	Viruses	0.12
912	936375	Mogibacterium sp. CM50	Bacteria	0.12
913	367121	Grapevine leafroll-associated virus 10	Viruses	0.12
914	31504	Tobacco ringspot virus satellite RNA	Viruses	0.12
915	104955	Lactobacillus frumenti	Bacteria	0.12
916	83526	Lactobacillus paralimentarius	Bacteria	0.12
917	1335	Streptococcus equinus	Bacteria	0.12
918	425254	Cellulosilyticum ruminicola	Bacteria	0.12
919	397290	Lachnospiraceae bacterium A2	Bacteria	0.12
920	1632	Lactobacillus oris	Bacteria	0.12
921	1288391	Actinomyces timonensis	Bacteria	0.11
922	1768874	Sinapis alba cryptic virus 1	Viruses	0.11
923	77768	Prevotella albensis	Bacteria	0.11
924	1852379	Veillonellaceae bacterium Marseille-P2911	Bacteria	0.11
925	1190620	Atopobium sp. ICM42b	Bacteria	0.11
926	546	Citrobacter freundii	Bacteria	0.11
927	1211388	Apple green crinkle associated virus	Viruses	0.11
928	1280686	Butyrivibrio sp. MC2013	Bacteria	0.11
929	1105031	Clostridium sp. MSTE9	Bacteria	0.11
930	59505	Actinotignum schaalii	Bacteria	0.11
931	188913	Cetobacterium somerae	Bacteria	0.11
932	65467	Cherry green ring mottle virus	Viruses	0.11
933	1105171	Bacteroides phage B124-14	Viruses	0.11
934	28129	Prevotella denticola	Bacteria	0.11
935	218923	Turnip rosette virus	Viruses	0.11
936	37128	Potato mop-top virus	Viruses	0.11
937	1348	Streptococcus parauberis	Bacteria	0.11
938	1232449	Clostridiales bacterium VE202-08	Bacteria	0.11
939	1712675	Turicibacter sp. H121	Bacteria	0.11
940	439703	Prevotella maculosa	Bacteria	0.10
941	1629	Weissella viridescens	Bacteria	0.10
942	1922434	Beihai mollusks virus 1	Viruses	0.10
943	42817	Corynebacterium argentoratense	Bacteria	0.10
944	52584	Brachyspira pilosicoli	Bacteria	0.10
945	47493	Lactobacillus panis	Bacteria	0.10
946	33945	Enterococcus avium	Bacteria	0.10
947	649739	Actinomyces sp. oral taxon 848	Bacteria	0.10
948	113287	Pseudoramibacter alactolyticus	Bacteria	0.10
949	53363	Brevibacterium mcbrellneri	Bacteria	0.10
950	68033	Carrot mottle virus	Viruses	0.10
951	28131	Prevotella intermedia	Bacteria	0.10
952	12431	Garlic virus C	Viruses	0.10
953	228603	Prevotella shahii	Bacteria	0.10
954	318464	Clostridium sulfidigenes	Bacteria	0.10
955	88233	Lactobacillus manihotivorans	Bacteria	0.10
956	881	Desulfovibrio vulgaris	Bacteria	0.10
957	47715	Lactobacillus rhamnosus	Bacteria	0.10

958	137732	Granulicatella elegans	Bacteria	0.10
959	1249	Weissella paramesenteroides	Bacteria	0.10
960	936591	Veillonella sp. ICM51a	Bacteria	0.10
961	167634	Grapevine rootstock stem lesion associated virus	Viruses	0.10
962	39681	Asparagus virus 2	Viruses	0.10
963	71032	Grapevine leafroll-associated virus 5	Viruses	0.10
964	259059	Lactobacillus satsumensis	Bacteria	0.09
965	178001	Leuconostoc inhae	Bacteria	0.09
966	712148	Aggregatibacter sp. oral taxon 458	Bacteria	0.09
967	12045	Potato leafroll virus	Viruses	0.09
968	1328	Streptococcus anginosus	Bacteria	0.09
969	56879	Oat blue dwarf virus	Viruses	0.09
970	99179	Bacteroides phage B40-8	Viruses	0.09
971	1501332	Oribacterium asaccharolyticum	Bacteria	0.09
972	12040	Barley yellow dwarf virus-PAV	Viruses	0.09
973	1054217	Thermoplasmatales archaeon BRNA1	Archaea	0.09
974	1511840	Primula malacoides virus 1	Viruses	0.09
975	2751	Carnobacterium maltaromaticum	Bacteria	0.09
976	90410	Streptococcus phage DT1	Viruses	0.09
977	1247	Oenococcus oeni	Bacteria	0.09
978	246618	Bifidobacterium thermacidophilum	Bacteria	0.09
979	33029	Anaerococcus hydrogenalis	Bacteria	0.09
980	348151	Lactobacillus siliginis	Bacteria	0.09
981	1588751	Tissierellia bacterium KA00581	Bacteria	0.09
982	33037	Anaerococcus vaginalis	Bacteria	0.09
983	714	Aggregatibacter actinomycetemcomitans	Bacteria	0.09
984	288000	Bradyrhizobium sp. BTAi1	Bacteria	0.09
985	582	Morganella morganii	Bacteria	0.09
986	68892	Streptococcus infantis	Bacteria	0.09
987	52280	Chilli veinal mottle virus	Viruses	0.09
988	4903	Cyberlindnera jadinii	Eukaryota	0.09
989	1933294	Impatiens necrotic spot tospovirus	Viruses	0.09
990	203168	Grapevine leafroll-associated virus 6	Viruses	0.09
991	1581114	Enterococcus sp. HMSC05C03	Bacteria	0.09
992	208086	Moroccan pepper virus	Viruses	0.08
993	12138	Maize chlorotic mottle virus	Viruses	0.08
994	1755753	Penicillium aurantiogriseum foetidus-like virus	Viruses	0.08
995	5476	Candida albicans	Eukaryota	0.08
996	1840217	Candidatus Arthromitus sp. SFB-turkey	Bacteria	0.08
997	190721	Ralstonia insidiosa	Bacteria	0.08
998	529	Ochrobactrum anthropi	Bacteria	0.08
999	97253	Eubacterium plexicaudatum	Bacteria	0.08
1000	1673726	Clostridiales bacterium SIT11	Bacteria	0.08
1001	273677	Microbacterium oleivorans	Bacteria	0.08
1002	29419	Helicobacter canis	Bacteria	0.08
1003	89463	Sacbrood virus	Viruses	0.08
1004	1347393	Bacteroides neonati	Bacteria	0.08
1005	860	Fusobacterium periodonticum	Bacteria	0.08

1006	1203556	Actinomyces sp. HPA0247	Bacteria	0.08
1007	348449	Raphanus sativus cryptic virus 1	Viruses	0.08
1008	1235835	Anaerotruncus sp. G3(2012)	Bacteria	0.08
1009	1661	Trueperella pyogenes	Bacteria	0.08
1010	1246	Leuconostoc lactis	Bacteria	0.08
1011	516703	Pontibacillus litoralis	Bacteria	0.08
1012	1111133	Peptoniphilus sp. BV3AC2	Bacteria	0.08
1013	1345	Streptococcus ferus	Bacteria	0.08
1014	485724	Melon severe mosaic tospovirus	Viruses	0.08
1015	1176736	Pitaya virus X	Viruses	0.08
1016	1324352	Chryseobacterium gallinarum	Bacteria	0.08
1017	37923	Kocuria kristinae	Bacteria	0.08
1018	1472764	Kallipyga massiliensis	Bacteria	0.07
1019	202566	Cherry rasp leaf virus	Viruses	0.07
1020	1852378	Veillonellaceae bacterium Marseille-P2974	Bacteria	0.07
1021	43995	Johnsonella ignava	Bacteria	0.07
1022	430710	Blueberry latent virus	Viruses	0.07
1023	119219	Cupriavidus metallidurans	Bacteria	0.07
1024	1922435	Beihai mollusks virus 2	Viruses	0.07
1025	12211	Plum pox virus	Viruses	0.07
1026	1232454	Clostridiales bacterium VE202-26	Bacteria	0.07
1027	253701	Zygocactus virus X	Viruses	0.07
1028	12202	Lettuce mosaic virus	Viruses	0.07
1029	1561964	Methanosphaera sp. WGK6	Archaea	0.07
1030	78257	Bifidobacterium saeculare	Bacteria	0.07
1031	839	Prevotella ruminicola	Bacteria	0.07
1032	5482	Candida tropicalis	Eukaryota	0.07
1033	1280670	Butyrivibrio sp. AD3002	Bacteria	0.07
1034	118748	Bulleidia extructa	Bacteria	0.07
1035	33962	Lactobacillus kefiri	Bacteria	0.07
1036	1697797	Actinobacteria bacterium UC5.1-1B11	Bacteria	0.07
1037	936549	Actinomyces sp. ICM54	Bacteria	0.07
1038	1333651	Bifidobacterium moukalabense	Bacteria	0.07
1039	4959	Debaryomyces hansenii	Eukaryota	0.07
1040	591166	Southern tomato virus	Viruses	0.07
1041	1694	Bifidobacterium pseudolongum	Bacteria	0.07
1042	1468413	Bacillus massilioanorexius	Bacteria	0.07
1043	35288	Grapevine virus A	Viruses	0.07
1044	33031	Peptoniphilus lacrimalis	Bacteria	0.07
1045	712430	Peptoniphilus sp. oral taxon 375	Bacteria	0.07
1046	326941	Raspberry leaf mottle virus	Viruses	0.07
1047	328061	Radish mosaic virus	Viruses	0.07
1048	12143	Cucumber necrosis virus	Viruses	0.07
1049	936574	Shuttleworthia sp. MSX8B	Bacteria	0.07
1050	253239	Ethanoligenens harbinense	Bacteria	0.07
1051	134358	Westerdykella cylindrica	Eukaryota	0.07
1052	1365	Lactococcus plantarum	Bacteria	0.07
1053	51288	Kluyvera ascorbata	Bacteria	0.06

1054	1080349	Saccharomyces eubayanus	Eukaryota	0.06
1055	712121	Actinomyces sp. oral taxon 181	Bacteria	0.06
1056	208084	Grapevine Algerian latent virus	Viruses	0.06
1057	1118058	Actinomyces sp. ph3	Bacteria	0.06
1058	1280	Staphylococcus aureus	Bacteria	0.06
1059	712156	Atopobium sp. oral taxon 199	Bacteria	0.06
1060	1235790	Eubacterium sp. 14-2	Bacteria	0.06
1061	1235798	Dorea sp. 5-2	Bacteria	0.06
1062	12222	Soybean mosaic virus	Viruses	0.06
1063	1299998	Olsenella scatoligenes	Bacteria	0.06
1064	1410616	Pseudobutyrivibrio sp. MD2005	Bacteria	0.06
1065	1739371	Streptococcus sp. HMSC064H09	Bacteria	0.06
1066	590403	Red clover vein mosaic virus	Viruses	0.06
1067	147712	Rhinovirus B	Viruses	0.06
1068	1276	Kytococcus sedentarius	Bacteria	0.06
1069	31713	Lettuce infectious yellows virus	Viruses	0.06
1070	118562	Arthrospira platensis	Bacteria	0.06
1071	1080712	Methanomassiliicoccus luminyensis	Archaea	0.06
1072	1720298	Peptoniphilus phoceensis	Bacteria	0.06
1073	172220	Blueberry red ringspot virus	Viruses	0.06
1074	614	Serratia liquefaciens	Bacteria	0.06
1075	1230730	Tissierellia bacterium S5-A11	Bacteria	0.06
1076	1050903	Pepper cryptic virus 2	Viruses	0.06
1077	54067	Xylophilus ampelinus	Bacteria	0.06
1078	35519	Mogibacterium timidum	Bacteria	0.06
1079	1529	Clostridium cadaveris	Bacteria	0.06
1080	453050	Sweet potato virus 2	Viruses	0.06
1081	1739537	Anaerococcus sp. HMSC075B03	Bacteria	0.06
1082	1161412	Prevotella sp. ICM33	Bacteria	0.06
1083	392416	Lactobacillus crustorum	Bacteria	0.06
1084	1358418	Sinorhizobium sp. GL28	Bacteria	0.06
1085	1774276	Hordeum vulgare endornavirus	Viruses	0.06
1086	936589	Veillonella sp. AS16	Bacteria	0.06
1087	1564	Desulfotomaculum ruminis	Bacteria	0.06
1088	1852373	Murdochiella sp. Marseille-P2341	Bacteria	0.05
1089	194326	Lactobacillus versmoldensis	Bacteria	0.05
1090	171523	Lactobacillus pantheris	Bacteria	0.05
1091	1281	Staphylococcus carnosus	Bacteria	0.05
1092	12139	Southern bean mosaic virus	Viruses	0.05
1093	1777865	Weissella sp. DD23	Bacteria	0.05
1094	1610	Lactobacillus coryniformis	Bacteria	0.05
1095	37372	Helicobacter bilis	Bacteria	0.05
1096	1686287	Kallipyga gabonensis	Bacteria	0.05
1097	1497954	Bacteroidales bacterium KA00344	Bacteria	0.05
1098	131082	Beet chlorosis virus	Viruses	0.05
1099	1297865	Bradyrhizobium sp. OHSU_III	Bacteria	0.05
1100	1261640	Eubacterium sp. 68-3-10	Bacteria	0.05
1101	1587	Lactobacillus helveticus	Bacteria	0.05

1102	1597	Lactobacillus paracasei	Bacteria	0.05
1103	1715211	Haemophilus sp. HMSC061E01	Bacteria	0.05
1104	507750	Peptoniphilus duerdenii	Bacteria	0.05
1105	31537	Lactococcus phage c2	Viruses	0.05
1106	92395	Black queen cell virus	Viruses	0.05
1107	195	Campylobacter coli	Bacteria	0.05
1108	28137	Prevotella veroralis	Bacteria	0.05
1109	119910	Ryegrass mottle virus	Viruses	0.05
1110	1343493	Grapevine satellite virus	Viruses	0.05
1111	379892	Passiflora latent carlavirus	Viruses	0.05
1112	28124	Porphyromonas endodontalis	Bacteria	0.05
1113	43130	Onion yellow dwarf virus	Viruses	0.05
1114	1235800	Lachnospiraceae bacterium 10-1	Bacteria	0.05
1115	339420	Blackberry chlorotic ringspot virus	Viruses	0.05
1116	177972	Shuttleworthia satelles	Bacteria	0.05
1117	1525173	Human circovirus VS6600022	Viruses	0.05
1118	12183	Potato virus X	Viruses	0.05
1119	11986	Carnation mottle virus	Viruses	0.05
1120	138950	Enterovirus C	Viruses	0.05
1121	137730	Facklamia ignava	Bacteria	0.05
1122	198112	Deformed wing virus	Viruses	0.05
1123	1579341	Streptococcus sp. 400_SSPC	Bacteria	0.05
1124	671230	Parvimonas sp. oral taxon 110	Bacteria	0.05
1125	1933295	Iris yellow spot tospovirus	Viruses	0.05
1126	1353	Enterococcus gallinarum	Bacteria	0.05
1127	1354	Enterococcus hirae	Bacteria	0.05
1128	1932006	Chicken associated smacovirus	Viruses	0.05
1129	392504	Turnip ringspot virus	Viruses	0.05
1130	134605	Fusobacterium equinum	Bacteria	0.05
1131	1000568	Megasphaera sp. UPII 199-6	Bacteria	0.05
1132	587	Providencia rettgeri	Bacteria	0.05
1133	46867	Clostridium chauvoei	Bacteria	0.05
1134	471858	Helicobacter magdeburgensis	Bacteria	0.05
1135	12238	Odontoglossum ringspot virus	Viruses	0.05
1136	876478	Tepidiphilus thermophilus	Bacteria	0.05
1137	28985	Kluyveromyces lactis	Eukaryota	0.05
1138	82347	Facklamia languida	Bacteria	0.05
1139	1080072	Streptococcus dentasini	Bacteria	0.05
1140	1764	Mycobacterium avium	Bacteria	0.05
1141	1840518	Gardnerella sp. 30-4	Bacteria	0.05
1142	167	Treponema succinifaciens	Bacteria	0.05
1143	936550	Atopobium sp. BS2	Bacteria	0.05
1144	1423347	Sclerotinia sclerotiorum hypovirus 2	Viruses	0.05
1145	516956	Grapevine virus E	Viruses	0.05
1146	1739422	Streptococcus sp. HMSC065C01	Bacteria	0.05
1147	5478	[Candida] glabrata	Eukaryota	0.05
1148	28348	Sweet clover necrotic mosaic virus	Viruses	0.05
1149	1384078	Prevotella sp. DNF00663	Bacteria	0.05

1150	1330491	Cosavirus A	Viruses	0.05
1151	140626	Lachnobacterium bovis	Bacteria	0.05
1152	173976	Cycas necrotic stunt virus	Viruses	0.05
1153	29359	Asaccharospora irregularis	Bacteria	0.05
1154	257464	Potato black ringspot virus	Viruses	0.05
1155	12271	Arabis mosaic virus	Viruses	0.05
1156	1425364	Carrot torradovirus 1	Viruses	0.05
1157	195054	Human parechovirus	Viruses	0.05
1158	83559	Chlamydia suis	Bacteria	0.05
1159	1323524	Red clover cryptic virus 2	Viruses	0.05
1160	225992	Comamonas kerstersii	Bacteria	0.05
1161	64958	Parietaria mottle virus	Viruses	0.05
1162	1719140	Klebsiella phage vB_KpnM_KB57	Viruses	0.05
1163	682370	Streptococcus phage Alq132	Viruses	0.05
1164	328396	Enterococcus aquimarinus	Bacteria	0.05
1165	53444	Lactobacillus lindneri	Bacteria	0.05
1166	1711684	Hot pepper endornavirus	Viruses	0.05
1167	595895	Drosophila A virus	Viruses	0.05
1168	41997	Enterococcus saccharolyticus	Bacteria	0.05
1169	909827	Pepper vein yellows virus	Viruses	0.05
1170	457388	Parabacteroides sp. 2_1_7	Bacteria	0.05
1171	1675609	Klebsiella phage Sushi	Viruses	0.05
1172	12432	Garlic virus B	Viruses	0.05
1173	936381	Selenomonas sp. CM52	Bacteria	0.05
1174	1715086	Streptococcus sp. HMSC078H03	Bacteria	0.05
1175	193120	Pea enation mosaic virus-2	Viruses	0.05
1176	664639	Kocuria salsicia	Bacteria	0.05
1177	374840	Enterobacteria phage phiX174 sensu lato	Viruses	0.05
1178	671216	Peptoniphilus sp. oral taxon 836	Bacteria	0.05
1179	12165	Chrysanthemum virus B	Viruses	0.05
1180	12167	Potato virus M	Viruses	0.05
1181	12161	Beet yellows virus	Viruses	0.05
1182	1520332	Blueberry mosaic associated virus	Viruses	0.05
1183	589537	Prevotella dentasini	Bacteria	0.05
1184	1739286	Anaerococcus sp. HMSC068A02	Bacteria	0.05
1185	12197	Bean yellow mosaic virus	Viruses	0.05
1186	1095727	Streptococcus sp. SK643	Bacteria	0.05
1187	257758	Streptococcus pseudopneumoniae	Bacteria	0.05
1188	71030	Chayote mosaic virus	Viruses	0.05
1189	66834	Rhopalosiphum padi virus	Viruses	0.05
1190	97139	Clostridium sp. ASF502	Bacteria	0.05
1191	57732	Enterococcus asini	Bacteria	0.05
1192	396268	Lactobacillus secaliphilus	Bacteria	0.05
1193	1654927	Klebsiella phage PKP126	Viruses	0.05
1194	28197	Arcobacter butzleri	Bacteria	0.05
1195	298	Pseudomonas marginalis	Bacteria	0.04
1196	1805472	Clostridium sp. Marseille-P2434	Bacteria	0.04
1197	1480694	Spirochaeta lutea	Bacteria	0.04

1198	399370	Lactobacillus ghanensis	Bacteria	0.04
1199	1282	Staphylococcus epidermidis	Bacteria	0.04
1200	35841	Bacillus thermoamylovorans	Bacteria	0.04
1201	46124	Granulicatella adiacens	Bacteria	0.04
1202	405212	Alicyclobacillus acidocaldarius	Bacteria	0.04
1203	578361	Soybean yellow mottle mosaic virus	Viruses	0.04
1204	1235799	Lachnospiraceae bacterium 3-2	Bacteria	0.04
1205	1101373	Tepidimonas fonticaldi	Bacteria	0.04
1206	1739413	Alloscardovia sp. HMSC034E08	Bacteria	0.04
1207	545	Citrobacter koseri	Bacteria	0.04
1208	46206	Pseudobutyrivibrio ruminis	Bacteria	0.04
1209	1658779	Porphyromonadaceae bacterium H1	Bacteria	0.04
1210	31749	Obuda pepper virus	Viruses	0.04
1211	79604	Denitrobacterium detoxificans	Bacteria	0.04
1212	39397	Candida sake	Eukaryota	0.04
1213	1923594	Wenzhou picorna-like virus 10	Viruses	0.04
1214	1674942	Caenibacillus caldisaponilyticus	Bacteria	0.04
1215	39497	Eubacterium xylanophilum	Bacteria	0.04
1216	1469950	Robinsoniella sp. KNHs210	Bacteria	0.04
1217	12275	Tomato black ring virus	Viruses	0.04
1218	129141	Citrus leaf blotch virus	Viruses	0.04
1219	1270	Micrococcus luteus	Bacteria	0.04
1220	29385	Staphylococcus saprophyticus	Bacteria	0.04
1221	713008	Parvimonas sp. oral taxon 393	Bacteria	0.04
1222	1433844	Prevotella sp. HJM029	Bacteria	0.04
1223	204933	Grapevine rupestris vein feathering virus	Viruses	0.04
1224	47736	Carrot mottle mimic virus	Viruses	0.04
1225	1579339	Streptococcus sp. 449_SSPC	Bacteria	0.04
1226	831	Butyrivibrio fibrisolvens	Bacteria	0.04
1227	1739452	Haemophilus sp. HMSC073C03	Bacteria	0.04
1228	1727	Corynebacterium variabile	Bacteria	0.04
1229	1323525	White clover cryptic virus 2	Viruses	0.04
1230	69823	Selenomonas sputigena	Bacteria	0.04
1231	102684	Streptococcus infantarius	Bacteria	0.04
1232	237258	Cloacibacterium normanense	Bacteria	0.04
1233	36911	Clavispora lusitaniae	Eukaryota	0.04
1234	106008	Curvibasidium cygneicollum	Eukaryota	0.04
1235	1921123	Salmonella virus 9NA	Viruses	0.04
1236	1815509	Bacillus phage AR9	Viruses	0.04
1237	49266	Fucus vesiculosus	Eukaryota	0.04
1238	1794912	Anaerosporomusa subterranea	Bacteria	0.04
1239	1581148	Clostridium sp. HMSC19A10	Bacteria	0.04
1240	152331	Lactobacillus parabuchneri	Bacteria	0.04
1241	683172	Astrovirus MLB2	Viruses	0.04
1242	264463	Anaerosporobacter mobilis	Bacteria	0.04
1243	76860	Streptococcus constellatus	Bacteria	0.04
1244	1860161	Streptococcus sp. CCUG 49591	Bacteria	0.04
1245	568715	Astrovirus MLB1	Viruses	0.04

1246	270256	Eggplant mottled crinkle virus	Viruses	0.04
1247	1739381	Streptococcus sp. HMSC072D03	Bacteria	0.04
1248	12230	Turnip mosaic virus	Viruses	0.04
1249	1930298	Chicken stool-associated gemycircularvirus	Viruses	0.04
1250	1133319	Bacteroides reticulotermitis	Bacteria	0.04
1251	1384082	Veillonellaceae bacterium DNF00751	Bacteria	0.04
1252	31721	Beet necrotic yellow vein virus	Viruses	0.04
1253	167481	Lactobacillus mindensis	Bacteria	0.04
1254	1316412	Streptococcus sp. HSISS3	Bacteria	0.04
1255	1279099	Sclerotinia sclerotiorum mitovirus 3	Viruses	0.04
1256	1161417	Streptococcus sp. SR4	Bacteria	0.04
1257	1597976	Enterococcus phage EFDG1	Viruses	0.04
1258	339	Xanthomonas campestris	Bacteria	0.04
1259	1330524	Salivirus A	Viruses	0.04
1260	28141	Cronobacter sakazakii	Bacteria	0.04
1261	10840	Beet curly top virus	Viruses	0.04
1262	138949	Enterovirus B	Viruses	0.04
1263	138948	Enterovirus A	Viruses	0.04
1264	584	Proteus mirabilis	Bacteria	0.04
1265	72750	Beet pseudoyellows virus	Viruses	0.04
1266	569	Hafnia alvei	Bacteria	0.04
1267	1165092	Lachnospiraceae bacterium JC7	Bacteria	0.04
1268	1392486	Prevotella sp. HUN102	Bacteria	0.04
1269	4909	Pichia kudriavzevii	Eukaryota	0.04
1270	1739356	Anaerococcus sp. HMSC065G05	Bacteria	0.04
1271	186189	Xylanimonas cellulosilytica	Bacteria	0.04
1272	1862960	Lactococcus phage M5938	Viruses	0.04
1273	571	Klebsiella oxytoca	Bacteria	0.04
1274	1423	Bacillus subtilis	Bacteria	0.04
1275	47985	Grapevine leafroll-associated virus 1	Viruses	0.04
1276	213633	Providence virus	Viruses	0.04
1277	1519	Clostridium tyrobutyricum	Bacteria	0.04
1278	1131702	Persea americana endornavirus 1	Viruses	0.04
1279	397287	Lachnospiraceae bacterium 28-4	Bacteria	0.04
1280	1608898	Haemophilus sp. HMSC71H05	Bacteria	0.04
1281	264634	Acholeplasma equifetale	Bacteria	0.04
1282	726	Haemophilus haemolyticus	Bacteria	0.04
1283	1748	Acidipropionibacterium acidipropionici	Bacteria	0.04
1284	12558	Sesbania mosaic virus	Viruses	0.04
1285	1592930	Yam latent virus	Viruses	0.04
1286	1930509	Husavirus sp.	Viruses	0.03
1287	85454	Alternanthera mosaic virus	Viruses	0.03
1288	294	Pseudomonas fluorescens	Bacteria	0.03
1289	218140	Bifidobacterium psychraerophilum	Bacteria	0.03
1290	290399	Arthrobacter sp. FB24	Bacteria	0.03
1291	550	Enterobacter cloacae	Bacteria	0.03
1292	333754	Alphapapillomavirus 10	Viruses	0.03
1293	1315956	Shigella phage pSf-1	Viruses	0.03

1294	69966	Macrococcus caseolyticus	Bacteria	0.03
1295	1027232	Groundnut ringspot and Tomato chlorotic spot virus reassortant	Viruses	0.03
1296	1922513	Beihai permutotetra-like virus 2	Viruses	0.03
1297	179628	Clostridium colicanis	Bacteria	0.03
1298	1702221	Faecalibaculum rodentium	Bacteria	0.03
1299	1141139	Enterobacteria phage vB_EcoP_ACG-C91	Viruses	0.03
1300	112436	Celery mosaic virus	Viruses	0.03
1301	548	Klebsiella aerogenes	Bacteria	0.03
1302	126385	Providencia alcalifaciens	Bacteria	0.03
1303	988645	Raspberry leaf blotch virus	Viruses	0.03
1304	1920861	Klebsiella virus KP36	Viruses	0.03
1305	354259	Lactococcus phage 936 sensu lato	Viruses	0.03
1306	546367	Hafnia paralvei	Bacteria	0.03
1307	13335	Anaerobiospirillum succiniciproducens	Bacteria	0.03
1308	248039	Mucispirillum schaedleri	Bacteria	0.03
1309	651609	Actinomyces sp. oral taxon 180	Bacteria	0.03
1310	12450	Saccharomyces cerevisiae killer virus M1	Viruses	0.03
1311	1723382	Peptoniphilaceae bacterium FC2	Bacteria	0.03
1312	373054	Calditerricola satsumensis	Bacteria	0.03
1313	241555	Helcococcus sueciensis	Bacteria	0.03
1314	1922835	Hubei arthropod virus 1	Viruses	0.03
1315	682382	HMO Astrovirus A	Viruses	0.03
1316	12208	Pea seed-borne mosaic virus	Viruses	0.03
1317	78259	Scardovia inopinata	Bacteria	0.03
1318	31770	Shallot virus X	Viruses	0.03
1319	12055	Tobacco necrosis virus A	Viruses	0.03
1320	111015	Actinomyces radicidentis	Bacteria	0.03
1321	1465756	Peptoniphilus grossensis	Bacteria	0.03
1322	469553	Delftia sp. JD2	Bacteria	0.03
1323	1870984	Anaerococcus mediterraneensis	Bacteria	0.03
1324	12313	Peanut stunt virus	Viruses	0.03
1325	1868658	Human astrovirus	Viruses	0.03
1326	157687	Leptotrichia wadei	Bacteria	0.03
1327	1582	Lactobacillus casei	Bacteria	0.03
1328	351091	Oscillibacter valericigenes	Bacteria	0.03
1329	35818	Helicobacter pullorum	Bacteria	0.03
1330	72539	Physalis mottle virus	Viruses	0.03
1331	1590596	Sphingobacterium sp. T2	Bacteria	0.03
1332	28112	Tannerella forsythia	Bacteria	0.03
1333	1756285	Maize associated totivirus	Viruses	0.03
1334	260742	Streptomyces sp. SS	Bacteria	0.03
1335	1111121	Atopobium sp. BV3Ac4	Bacteria	0.03
1336	1343920	Apricot vein clearing associated virus	Viruses	0.03
1337	47669	Olive latent virus 1	Viruses	0.03
1338	1567484	Lactobacillus phage LfeInf	Viruses	0.03
1339	329853	Escherichia virus BZ13	Viruses	0.03
1340	329852	Escherichia virus MS2	Viruses	0.03

1341	197	Campylobacter jejuni	Bacteria	0.03
1342	1156433	Streptococcus sp. I-P16	Bacteria	0.03
1343	1718158	Enterococcus phage IME-EFm5	Viruses	0.03
1344	1702287	Negativicoccus massiliensis	Bacteria	0.03
1345	1739309	Streptococcus sp. HMSC034E03	Bacteria	0.03
1346	158787	Bifidobacterium scardovii	Bacteria	0.03
1347	5353	Lentinula edodes	Eukaryota	0.03
1348	1859694	Haemophilus sp. CCUG 66565	Bacteria	0.03
1349	28134	Prevotella oralis	Bacteria	0.03
1350	227942	Lactobacillus gastricus	Bacteria	0.03
1351	293371	Lactobacillus oligofermentans	Bacteria	0.03
1352	298338	Lactobacillus phage LP65	Viruses	0.03
1353	1435146	Morganella sp. EGD-HP17	Bacteria	0.03
1354	471285	Lettuce yellow mottle virus	Viruses	0.03
1355	1891289	Sporanaerobacter sp. PP17-6a	Bacteria	0.03
1356	1911008	Escherichia virus K1G	Viruses	0.03
1357	1314	Streptococcus pyogenes	Bacteria	0.03
1358	630199	Grapevine Syrah virus 1	Viruses	0.03
1359	712531	Selenomonas sp. oral taxon 137	Bacteria	0.03
1360	111418	Zucchini green mottle mosaic virus	Viruses	0.03
1361	151276	Bacteroides coprosuis	Bacteria	0.03
1362	1871031	Olsenella sp. Marseille-P3256	Bacteria	0.03
1363	469594	Bifidobacterium sp. 12_1_47BFAA	Bacteria	0.03
1364	95609	Herbaspirillum sp. B39	Bacteria	0.03
1365	1134687	Klebsiella michiganensis	Bacteria	0.03
1366	51354	Maize chlorotic dwarf virus	Viruses	0.03
1367	35703	Citrobacter amalonaticus	Bacteria	0.03
1368	83231	Prevotella brevis	Bacteria	0.03
1369	388038	Cucumber mottle virus	Viruses	0.03
1370	463676	Rhinovirus C	Viruses	0.03
1371	282402	Prevotella multiformis	Bacteria	0.03
1372	57706	Citrobacter braakii	Bacteria	0.03
1373	908834	Grapevine associated narnavirus-1	Viruses	0.03
1374	43675	Rothia mucilaginosa	Bacteria	0.03
1375	134632	American plum line pattern virus	Viruses	0.03
1376	1658008	Dysgonomonas sp. BGC7	Bacteria	0.03
1377	112023	Streptococcus phage 7201	Viruses	0.03
1378	85154	Streptococcus phage O1205	Viruses	0.03
1379	2293	Desulfobacter postgatei	Bacteria	0.03
1380	67824	Citrobacter farmeri	Bacteria	0.03
1381	169292	Corynebacterium aurimucosum	Bacteria	0.03
1382	539813	Enterobacter mori	Bacteria	0.02
1383	1608882	Haemophilus sp. HMSC61B11	Bacteria	0.02
1384	277	Meiothermus ruber	Bacteria	0.02
1385	1163671	Clostridium sp. 12(A)	Bacteria	0.02
1386	1914853	Escherichia virus V5	Viruses	0.02
1387	1914855	Escherichia virus FV3	Viruses	0.02
1388	1914854	Escherichia virus JES2013	Viruses	0.02

1389	136609	Leuconostoc kimchii	Bacteria	0.02
1390	151414	Afipia birgiae	Bacteria	0.02
1391	217686	Little cherry virus 1	Viruses	0.02
1392	73098	Kluyvera georgiana	Bacteria	0.02
1393	52253	Candida sojae	Eukaryota	0.02
1394	1698360	Klebsiella phage JD18	Viruses	0.02
1395	191217	Cereal yellow dwarf virus-RPV	Viruses	0.02
1396	1301100	[Clostridium] dakarense	Bacteria	0.02
1397	1795648	Picornavirales Tottori-HG1	Viruses	0.02
1398	149016	Streptococcus urinalis	Bacteria	0.02
1399	1235793	Lachnospiraceae bacterium COE1	Bacteria	0.02
1400	1852368	Prevotellaceae bacterium Marseille-P2826	Bacteria	0.02
1401	12227	Tobacco etch virus	Viruses	0.02
1402	331278	Yersinia phage phiR1-37	Viruses	0.02
1403	1612	Lactobacillus farciminis	Bacteria	0.02
1404	948870	Enterobacteria phage phi92	Viruses	0.02
1405	114871	Zygosaccharomyces bailii virus Z	Viruses	0.02
1406	1920862	Klebsiella virus 1513	Viruses	0.02
1407	570949	Carrot mottle mimic virus satellite RNA	Viruses	0.02
1408	337048	Alphapapillomavirus 1	Viruses	0.02
1409	1913024	White clover mottle virus	Viruses	0.02
1410	71452	Enterococcus raffinosus	Bacteria	0.02
1411	1522179	Asterionellopsis glacialis RNA virus	Viruses	0.02
1412	1496722	Butyrivibrio sp. AE2005	Bacteria	0.02
1413	1408324	Lachnospiraceae bacterium MC2017	Bacteria	0.02
1414	1737425	Corynebacterium provencense	Bacteria	0.02
1415	4950	Torulaspora delbrueckii	Eukaryota	0.02
1416	1600	Lactobacillus acetotolerans	Bacteria	0.02
1417	179878	Sphingomonas elodea	Bacteria	0.02
1418	216463	Lactobacillus spicheri	Bacteria	0.02
1419	1715012	Enterococcus sp. HMSC072H05	Bacteria	0.02
1420	1288120	Anaerococcus senegalensis	Bacteria	0.02
1421	539	Eikenella corrodens	Bacteria	0.02
1422	12024	Pseudomonas phage PRR1	Viruses	0.02
1423	1195163	Amazon lily mild mottle virus	Viruses	0.02
1424	1923170	Hubei polero-like virus 2	Viruses	0.02
1425	154339	Little cherry virus 2	Viruses	0.02
1426	179636	Alicycliphilus denitrificans	Bacteria	0.02
1427	39804	Escherichia virus FI	Viruses	0.02
1428	246144	Enterococcus italicus	Bacteria	0.02
1429	685899	Papaya lethal yellowing virus	Viruses	0.02
1430	312295	Cotton leafroll dwarf virus	Viruses	0.02
1431	1739439	Corynebacterium sp. HMSC076D02	Bacteria	0.02
1432	1493	Clostridium cellulovorans	Bacteria	0.02
1433	796937	Peptoanaerobacter stomatis	Bacteria	0.02
1434	470565	Prevotella histicola	Bacteria	0.02
1435	451457	Lactococcus chungangensis	Bacteria	0.02
1436	1229753	Escherichia phage phAPEC8	Viruses	0.02

1437	1581074	Granulicatella sp. HMSC31F03	Bacteria	0.02
1438	1581071	Granulicatella sp. HMSC30F09	Bacteria	0.02
1439	425941	Prevotella nanceiensis	Bacteria	0.02
1440	464322	Veillonella magna	Bacteria	0.02
1441	425010	Botryotinia fuckeliana partitivirus 1	Viruses	0.02
1442	1470356	Clostridium ihumii	Bacteria	0.02
1443	1287488	Prevotella sp. S7 MS 2	Bacteria	0.02
1444	443746	Asparagus virus 1	Viruses	0.02
1445	157691	Leptotrichia shahii	Bacteria	0.02
1446	444193	Botrytis cinerea mitovirus 1	Viruses	0.02
1447	29388	Staphylococcus capitis	Bacteria	0.02
1448	1385385	Streptococcus phage TP-778L	Viruses	0.02
1449	1354300	Peptoniphilus sp. ChDC B134	Bacteria	0.02
1450	200	Campylobacter curvus	Bacteria	0.02
1451	203	Campylobacter rectus	Bacteria	0.02
1452	12049	Soybean dwarf virus	Viruses	0.02
1453	1923116	Hubei picorna-like virus 36	Viruses	0.02
1454	10829	Squash leaf curl virus	Viruses	0.02
1455	1857568	Macellibacteroides sp. HH-ZS	Bacteria	0.02
1456	36343	Lactococcus phage bIL67	Viruses	0.02
1457	359987	Rhizosolenia setigera RNA virus 01	Viruses	0.02
1458	1206545	Klebsiella phage 0507-KN2-1	Viruses	0.02
1459	1868652	High Plains wheat mosaic virus	Viruses	0.02
1460	1739450	Actinomyces sp. HMSC062G12	Bacteria	0.02
1461	1547495	Salivirus FHB	Viruses	0.02
1462	59241	Streptococcus phage Dp-1	Viruses	0.02
1463	156976	Corynebacterium riegelii	Bacteria	0.02
1464	1589	Lactobacillus pentosus	Bacteria	0.02
1465	1517899	Kytococcus sp. CUA-901	Bacteria	0.02
1466	1280675	Bifidobacterium sp. AGR2158	Bacteria	0.02
1467	1759399	Streptococcus sp. A12	Bacteria	0.02
1468	1563222	Citrobacter pasteurii	Bacteria	0.02
1469	181675	Lactobacillus coleohominis	Bacteria	0.02
1470	357278	Lactobacillus parabrevis	Bacteria	0.02
1471	1692238	Enterobacter sp. FY-07	Bacteria	0.02
1472	81931	Sweet potato chlorotic stunt virus	Viruses	0.02
1473	1739465	Enterococcus sp. HMSC076E04	Bacteria	0.02
1474	179838	Lactobacillus diolivorans	Bacteria	0.02
1475	1410625	Lachnospiraceae bacterium MD2004	Bacteria	0.02
1476	218667	Oyster mushroom spherical virus	Viruses	0.02
1477	39443	Carnation Italian ringspot virus	Viruses	0.02
1478	111105	Porphyromonas gulae	Bacteria	0.02
1479	12209	Pepper mottle virus	Viruses	0.02
1480	50948	Enterobacteria phage RB49	Viruses	0.02
1481	381742	Lactobacillus camelliae	Bacteria	0.02
1482	485	Neisseria gonorrhoeae	Bacteria	0.02
1483	1647408	Klebsiella phage KLPN1	Viruses	0.02
1484	693582	Pseudomonas phage phi-2	Viruses	0.02

1485	1693	Bifidobacterium minimum	Bacteria	0.02
1486	1272	Kocuria varians	Bacteria	0.02
1487	1739491	Streptococcus sp. HMSC067H01	Bacteria	0.02
1488	1268254	Peptoniphilus timonensis	Bacteria	0.02
1489	1445858	Enterococcus phage IME-EFm1	Viruses	0.02
1490	641148	Neisseria sp. oral taxon 014	Bacteria	0.02
1491	36427	Rotavirus C	Viruses	0.02
1492	1183241	Persimmon cryptic virus	Viruses	0.02
1493	1852625	Klebsiella phage vB_KpnM_KpV477	Viruses	0.02
1494	202789	Actinobaculum massiliense	Bacteria	0.02
1495	1485952	Enterobacter massiliensis	Bacteria	0.02
1496	1965376	Escherichia virus EC6	Viruses	0.02
1497	36015	Pichia kluyveri	Eukaryota	0.02
1498	134821	Ureaplasma parvum	Bacteria	0.02
1499	61435	Dehalococcoides mccartyi	Bacteria	0.02
1500	375175	Lactobacillus backii	Bacteria	0.02
1501	632112	Lactobacillus phage Lb338-1	Viruses	0.02
1502	43131	Tissierella praeacuta	Bacteria	0.02
1503	424716	Salmonella phage Vi II-E1	Viruses	0.02
1504	150055	Streptococcus lutetiensis	Bacteria	0.02
1505	1908263	Rodentibacter trehalosifermentans	Bacteria	0.02
1506	105219	Ralstonia mannitolilytica	Bacteria	0.02
1507	33934	Anoxybacillus flavithermus	Bacteria	0.02
1508	435910	Franconibacter pulveris	Bacteria	0.02
1509	12179	Foxtail mosaic virus	Viruses	0.02
1510	689781	Oribacterium sp. NK2B42	Bacteria	0.02
1511	1922725	Beihai tombus-like virus 4	Viruses	0.02
1512	344022	Escherichia virus K1E	Viruses	0.02
1513	33905	Bifidobacterium thermophilum	Bacteria	0.02
1514	1654357	La Jolla virus	Viruses	0.02
1515	1654356	Thika virus	Viruses	0.02
1516	481720	Lactobacillus otakiensis	Bacteria	0.02
1517	1720495	Escherichia phage slur16	Viruses	0.02
1518	5412	Cystofilobasidium capitatum	Eukaryota	0.02
1519	129875	Human mastadenovirus A	Viruses	0.02
1520	1756832	Phasey bean mild yellows virus	Viruses	0.02
1521	1906334	Corynebacterium sp. NML140438	Bacteria	0.02
1522	753670	Pea necrotic yellow dwarf virus	Viruses	0.02
1523	53655	Pichia fermentans	Eukaryota	0.02
1524	1032457	Passion fruit mosaic virus	Viruses	0.02
1525	585	Proteus vulgaris	Bacteria	0.02
1526	580	Kluyvera cryocrescens	Bacteria	0.02
1527	1177630	American hop latent virus	Viruses	0.02
1528	1111137	Slackia sp. CM382	Bacteria	0.02
1529	1349	Streptococcus uberis	Bacteria	0.02
1530	1509	Clostridium sporogenes	Bacteria	0.02
1531	351495	Raphanus sativus cryptic virus 2	Viruses	0.02
1532	52768	Actinomyces georgiae	Bacteria	0.02

1533	373058	Tomato bushy stunt virus satellite RNA	Viruses	0.02
1534	227507	Strawberry pallidosis-associated virus	Viruses	0.02
1535	1475062	Porcine stool-associated circular virus 4	Viruses	0.02
1536	54291	Raoultella ornithinolytica	Bacteria	0.02
1537	1889813	Anaerolineaceae bacterium oral taxon 439	Bacteria	0.02
1538	1287640	Anaerococcus obesiensis	Bacteria	0.02
1539	671224	Selenomonas artemidis	Bacteria	0.02
1540	1785087	Candidatus Protochlamydia sp. W-9	Bacteria	0.02
1541	1517	Thermoanaerobacterium thermosaccharolyticum	Bacteria	0.02
1542	936577	Streptococcus sp. AS14	Bacteria	0.02
1543	1537165	Porcine stool-associated circular virus 6	Viruses	0.02
1544	1923554	Wenzhou bivalvia virus 2	Viruses	0.02
1545	1631	Fructobacillus fructosus	Bacteria	0.02
1546	229549	Streptococcus minor	Bacteria	0.02
1547	27288	Naumovozyma castellii	Eukaryota	0.01
1548	1211480	Persimmon virus A	Viruses	0.01
1549	439016	Marine RNA virus JP-B	Viruses	0.01
1550	407975	Prevotella pleuritidis	Bacteria	0.01
1551	563038	Streptococcus sp. M334	Bacteria	0.01
1552	1408894	Red clover cryptic virus 1	Viruses	0.01
1553	60133	Prevotella pallens	Bacteria	0.01
1554	448384	Enterobacteria phage Phi1	Viruses	0.01
1555	1781	Mycobacterium marinum	Bacteria	0.01
1556	113574	Hyphomicrobium sp. GJ21	Bacteria	0.01
1557	1656	Actinomyces viscosus	Bacteria	0.01
1558	656024	Frankia symbiont of Datisca glomerata	Bacteria	0.01
1559	1805471	Clostridium sp. Marseille-P2415	Bacteria	0.01
1560	313439	Streptococcus massiliensis	Bacteria	0.01
1561	938293	Anaerococcus provenciensis	Bacteria	0.01
1562	554	Pectobacterium carotovorum	Bacteria	0.01
1563	1739400	Corynebacterium sp. HMSC069E04	Bacteria	0.01
1564	1631871	Weissella jogaejeotgali	Bacteria	0.01
1565	1581133	Actinomyces sp. HMSC08A09	Bacteria	0.01
1566	178214	Facklamia hominis	Bacteria	0.01
1567	336988	Oenococcus kitaharae	Bacteria	0.01
1568	1914856	Escherichia virus FFH2	Viruses	0.01
1569	1283	Staphylococcus haemolyticus	Bacteria	0.01
1570	1814960	Streptococcus virus 9874	Viruses	0.01
1571	43263	Pseudomonas alcaligenes	Bacteria	0.01
1572	1745712	Anaerococcus sp. Marseille-P2143	Bacteria	0.01
1573	185008	Butyrivibrio hungatei	Bacteria	0.01
1574	656083	Barley yellow striate mosaic virus	Viruses	0.01
1575	1923133	Hubei picorna-like virus 51	Viruses	0.01
1576	2274	Desulfurococcus mobilis	Archaea	0.01
1577	53346	Enterococcus mundtii	Bacteria	0.01
1578	1504	Clostridium septicum	Bacteria	0.01
1579	12224	Sugarcane mosaic virus	Viruses	0.01
1580	1433126	Mucinivorans hirudinis	Bacteria	0.01

1581	29379	Staphylococcus auricularis	Bacteria	0.01
1582	1568973	Botrytis cinerea RNA virus 1	Viruses	0.01
1583	435842	Streptococcus sp. C150	Bacteria	0.01
1584	33936	Aeribacillus pallidus	Bacteria	0.01
1585	1965385	Escherichia virus wV8	Viruses	0.01
1586	1965384	Erwinia virus Ea214	Viruses	0.01
1587	936563	Fusobacterium sp. CM22	Bacteria	0.01
1588	129951	Human mastadenovirus C	Viruses	0.01
1589	713030	Selenomonas sp. oral taxon 136	Bacteria	0.01
1590	59310	Streptococcus macedonicus	Bacteria	0.01
1591	1823756	Actinomycetaceae bacterium BA112	Bacteria	0.01
1592	28227	Mycoplasma penetrans	Bacteria	0.01
1593	1383	Atopobium rimae	Bacteria	0.01
1594	228582	Cereal yellow dwarf virus-RPS	Viruses	0.01
1595	40091	Helcococcus kunzii	Bacteria	0.01
1596	1715007	Rothia sp. HMSC071B01	Bacteria	0.01
1597	1169350	Citrobacter sp. KTE32	Bacteria	0.01
1598	150285	Garlic virus E	Viruses	0.01
1599	1219585	Arcanobacterium sp. S3PF19	Bacteria	0.01
1600	31973	Eggerthia catenaformis	Bacteria	0.01
1601	77635	Bifidobacterium subtile	Bacteria	0.01
1602	1608993	Pseudomonas sp. DSM 28140	Bacteria	0.01
1603	2424	Fervidobacterium nodosum	Bacteria	0.01
1604	1675607	Klebsiella phage Matisse	Viruses	0.01
1605	94009	Thermicanus aegyptius	Bacteria	0.01
1606	204042	Dickeya zeae	Bacteria	0.01
1607	1922438	Beihai narna-like virus 11	Viruses	0.01
1608	12215	Potato virus A	Viruses	0.01
1609	936554	Campylobacter sp. FOBRC14	Bacteria	0.01
1610	1602	Lactobacillus alimentarius	Bacteria	0.01
1611	1607	Lactobacillus bifermentans	Bacteria	0.01
1612	1739310	Turicella sp. HMSC076G08	Bacteria	0.01
1613	1739315	Globicatella sp. HMSC072A10	Bacteria	0.01
1614	1566990	Streptococcus phage SpSL1	Viruses	0.01
1615	388452	Lactococcus phage KSY1	Viruses	0.01
1616	1888167	Enterobacter sp. ku-bf2	Bacteria	0.01
1617	37206	Helicoverpa armigera stunt virus	Viruses	0.01
1618	1551	Clostridium aurantibutyricum	Bacteria	0.01
1619	1914894	Escherichia virus 4MG	Viruses	0.01
1620	1870933	Enterobacter cloacae complex sp. 20432	Bacteria	0.01
1621	368736	Maracuja mosaic virus	Viruses	0.01
1622	132477	Kalanchoe latent virus	Viruses	0.01
1623	1715019	Enterococcus sp. HMSC064A12	Bacteria	0.01
1624	1542743	Caribou feces-associated gemycircularvirus	Viruses	0.01
1625	130309	Human mastadenovirus F	Viruses	0.01
1626	253702	Opuntia virus X	Viruses	0.01
1627	1519399	Sewage-associated gemycircularvirus 4	Viruses	0.01
1628	46076	Artichoke latent virus	Viruses	0.01

1629	307486	Tepidimonas taiwanensis	Bacteria	0.01
1630	328430	Chickpea chlorotic stunt virus	Viruses	0.01
1631	246432	Staphylococcus equorum	Bacteria	0.01
1632	1567453	Lactobacillus phage LfeSau	Viruses	0.01
1633	81947	Vagococcus lutrae	Bacteria	0.01
1634	12203	Maize dwarf mosaic virus	Viruses	0.01
1635	1329838	Enterobacter sp. BIDMC 26	Bacteria	0.01
1636	148604	Lactobacillus ingluviei	Bacteria	0.01
1637	470166	Tomato marchitez virus	Viruses	0.01
1638	1739430	Streptococcus sp. HMSC078D09	Bacteria	0.01
1639	1495	Clostridium cylindrosporum	Bacteria	0.01
1640	129395	Botrytis virus F	Viruses	0.01
1641	2094	Mycoplasma arginini	Bacteria	0.01
1642	208962	Escherichia albertii	Bacteria	0.01
1643	157228	Jeotgalicoccus psychrophilus	Bacteria	0.01
1644	123841	Helicobacter canadensis	Bacteria	0.01
1645	37961	Atkinsonella hypoxylon virus	Viruses	0.01
1646	1335616	Lactobacillus wasatchensis	Bacteria	0.01
1647	74381	Undaria pinnatifida	Eukaryota	0.01
1648	1581075	Neisseria sp. HMSC31F04	Bacteria	0.01
1649	1581072	Corynebacterium sp. HMSC30G07	Bacteria	0.01
1650	1930302	Chicken stool-associated circular virus	Viruses	0.01
1651	1871034	Propionimicrobium sp. Marseille-P3275	Bacteria	0.01
1652	160454	Enterococcus pallens	Bacteria	0.01
1653	688701	Chiltepin yellow mosaic virus	Viruses	0.01
1654	256701	Glutamicibacter arilaitensis	Bacteria	0.01
1655	111970	Kyuri green mottle mosaic virus	Viruses	0.01
1656	641487	Lactococcus phage P087	Viruses	0.01
1657	1232427	Corynebacterium ihumii	Bacteria	0.01
1658	747	Pasteurella multocida	Bacteria	0.01
1659	1540094	Citrobacter phage Moogle	Viruses	0.01
1660	43305	Butyrivibrio proteoclasticus	Bacteria	0.01
1661	440518	Sphingobium lucknowense	Bacteria	0.01
1662	10726	Escherichia virus T5	Viruses	0.01
1663	1923725	Wuhan insect virus 21	Viruses	0.01
1664	2718	Cardiobacterium hominis	Bacteria	0.01
1665	712976	Lachnospiraceae bacterium oral taxon 082	Bacteria	0.01
1666	1033736	Brevibacterium senegalense	Bacteria	0.01
1667	936578	Streptococcus sp. AS20	Bacteria	0.01
1668	36809	Mycobacterium abscessus	Bacteria	0.01
1669	936572	Selenomonas sp. FOBRC6	Bacteria	0.01
1670	1386092	Cellulomonas carbonis	Bacteria	0.01
1671	108486	Corynebacterium falsenii	Bacteria	0.01
1672	1581089	Corynebacterium sp. HMSC11E11	Bacteria	0.01
1673	327277	Bifidobacterium crudilactis	Bacteria	0.01
1674	53422	Thermobrachium celere	Bacteria	0.01
1675	456999	Rhizoctonia solani	Eukaryota	0.01
1676	1770265	Alfalfa enamovirus-1	Viruses	0.01

1677	1930269	Bermuda grass latent virus	Viruses	0.01
1678	129143	Cherry necrotic rusty mottle virus	Viruses	0.01
1679	709323	Fructobacillus tropaeoli	Bacteria	0.01
1680	56689	Mycobacterium mucogenicum	Bacteria	0.01
1681	1640536	Arthrospira sp. TJSD091	Bacteria	0.01
1682	295090	Olive mild mosaic virus	Viruses	0.01
1683	1581067	Kytococcus sp. HMSC28H12	Bacteria	0.01
1684	1581069	Corynebacterium sp. HMSC29G08	Bacteria	0.01
1685	622	Shigella dysenteriae	Bacteria	0.01
1686	1871025	Ndongobacter massiliensis	Bacteria	0.01
1687	1381007	Grapevine red-blotch associated virus	Viruses	0.01
1688	147709	Carnobacterium inhibens	Bacteria	0.01
1689	53413	Xanthomonas axonopodis	Bacteria	0.01
1690	61651	Serratia ficaria	Bacteria	0.01
1691	72520	Nannochloropsis gaditana	Eukaryota	0.01
1692	1545701	Lactobacillus sp. wkB10	Bacteria	0.01
1693	1923266	Hubei tombus-like virus 2	Viruses	0.01
1694	1119528	Methyloversatilis discipulorum	Bacteria	0.01
1695	154621	Enterococcus phoeniculicola	Bacteria	0.01
1696	1303256	Sphingobium sp. DC-2	Bacteria	0.01
1697	201	Campylobacter lari	Bacteria	0.01
1698	1879023	Mycobacterium sp. djl-10	Bacteria	0.01
1699	1923604	Wenzhou picorna-like virus 2	Viruses	0.01
1700	135080	Selenomonas flueggei	Bacteria	0.01
1701	12188	White clover mosaic virus	Viruses	0.01
1702	364298	Janibacter hoylei	Bacteria	0.01
1703	1527519	Escherichia phage Av-05	Viruses	0.01
1704	2210	Methanosarcina thermophila	Archaea	0.01
1705	1686381	Citrobacter sp. MGH106	Bacteria	0.01
1706	936562	Fusobacterium sp. CM21	Bacteria	0.01
1707	12268	Carnation ringspot virus	Viruses	0.01
1708	10752	Escherichia phage N4	Viruses	0.01
1709	12267	Red clover necrotic mosaic virus	Viruses	0.01
1710	44562	Pothos latent virus	Viruses	0.01
1711	4929	Meyerozyma guilliermondii	Eukaryota	0.01
1712	303541	Lactobacillus apis	Bacteria	0.01
1713	269666	Streptococcus marimammalium	Bacteria	0.01
1714	78344	Bifidobacterium gallinarum	Bacteria	0.01
1715	83554	Chlamydia psittaci	Bacteria	0.01
1716	156978	Corynebacterium imitans	Bacteria	0.01
1717	1776109	Goose dicistrovirus	Viruses	0.01
1718	29394	Dolosigranulum pigrum	Bacteria	0.01
1719	633	Yersinia pseudotuberculosis	Bacteria	0.01
1720	1090134	Salmonella phage SPN3US	Viruses	0.01
1721	1549858	Sphingomonas taxi	Bacteria	0.01
1722	1505227	Aeromonas phage pAh6-C	Viruses	0.01
1723	1229751	Lactococcus phage BM13	Viruses	0.01
1724	1100043	Apis mellifera filamentous virus	Viruses	0.01

1725	12465	Barley yellow mosaic virus	Viruses	0.01
1726	61648	Kluyvera intermedia	Bacteria	0.01
1727	1193095	Lactobacillus hokkaidonensis	Bacteria	0.01
1728	1920759	Escherichia virus IME11	Viruses	0.01
1729	1795832	Eikenella sp. NML130454	Bacteria	0.01
1730	55211	Erwinia persicina	Bacteria	0.01
1731	55507	Schwartzia succinivorans	Bacteria	0.01
1732	712119	Actinomyces sp. oral taxon 175	Bacteria	0.01
1733	4896	Schizosaccharomyces pombe	Eukaryota	0.01
1734	1686394	Enterobacter sp. MGH128	Bacteria	0.01
1735	94625	Ochrobactrum intermedium	Bacteria	0.01
1736	1235479	Pelosinus sp. HCF1	Bacteria	0.01
1737	1912598	Cherry associated luteovirus	Viruses	0.01
1738	400946	Wohlfahrtiimonas chitiniclastica	Bacteria	0.01
1739	1768771	Streptococcus sp. CCH8-G7	Bacteria	0.01
1740	1739264	Corynebacterium sp. HMSC065D07	Bacteria	0.01
1741	752	Pasteurella bettyae	Bacteria	0.01
1742	750	Gallibacterium anatis	Bacteria	0.01
1743	45972	Staphylococcus pasteuri	Bacteria	0.01
1744	1770210	Micrococcus sp. CH7	Bacteria	0.01
1745	267364	Lactobacillus acidifarinae	Bacteria	0.01
1746	56407	Hanseniaspora occidentalis	Eukaryota	0.01
1747	646010	Suakwa aphid-borne yellows virus	Viruses	0.01
1748	1462681	Pepo aphid-borne yellows virus	Viruses	0.01
1749	1462682	Luffa aphid-borne yellows virus	Viruses	0.01
1750	32629	Indian peanut clump virus	Viruses	0.01
1751	43771	Corynebacterium urealyticum	Bacteria	0.01
1752	28264	Arcanobacterium haemolyticum	Bacteria	0.01
1753	198	Campylobacter hyointestinalis	Bacteria	0.01
1754	1907766	Pseudomonas sp. BS-2016	Bacteria	0.01
1755	1156431	Streptococcus sp. I-G2	Bacteria	0.01
1756	46436	Beet soil-borne virus	Viruses	0.01
1757	1220025	Pokeweed mosaic virus	Viruses	0.01
1758	1325933	Clostridium polynesiense	Bacteria	0.01
1759	438780	Lactobacillus phage phiPYB5	Viruses	0.01
1760	2162	Methanobacterium formicicum	Archaea	0.01
1761	1933261	Groundnut bud necrosis tospovirus	Viruses	0.01
1762	1898961	Kluyvera intestini	Bacteria	0.01
1763	53343	Desulfotomaculum aeronauticum	Bacteria	0.01
1764	1739479	Actinomyces sp. HMSC065F12	Bacteria	0.01
1765	1768764	Streptococcus sp. CCH5-D3	Bacteria	0.01
1766	59803	Sphingomonas echinoides	Bacteria	0.01
1767	71237	Staphylococcus vitulinus	Bacteria	0.01
1768	78541	Streptococcus phage Sfi11	Viruses	0.01
1769	1714265	Klebsiella sp. KGM-IMP216	Bacteria	0.01
1770	264076	Horseradish latent virus	Viruses	0.01
1771	1661745	Haemophilus sp. C1	Bacteria	0.01
1772	28447	Clavibacter michiganensis	Bacteria	0.01

1773	1222338	Enterobacteria phage GEC-3S	Viruses	0.01
1774	1739496	Prevotella sp. HMSC069G02	Bacteria	0.01
1775	1581143	Arthrobacter sp. HMSC08H08	Bacteria	0.01
1776	1280676	Butyrivibrio sp. WCD3002	Bacteria	0.01
1777	28042	Saccharopolyspora rectivirgula	Bacteria	0.01
1778	1622070	Paenibacillus sp. GM2	Bacteria	0.01
1779	12402	Streptococcus phage EJ-1	Viruses	0.01
1780	186538	Zaire ebolavirus	Viruses	0.01
1781	51680	Ribgrass mosaic virus	Viruses	0.01
1782	1541211	Cripavirus NB-1/2011/HUN	Viruses	0.01
1783	61647	Pluralibacter gergoviae	Bacteria	0.01
1784	1055192	Comamonas sp. B-9	Bacteria	0.01
1785	1873985	Salmonella phage IME207	Viruses	0.01
1786	1923094	Hubei picorna-like virus 15	Viruses	0.01
1787	537874	Streptococcus phage PH15	Viruses	0.01
1788	1920779	Klebsiella virus SU552A	Viruses	0.01
1789	1514105	Erysipelothrix larvae	Bacteria	0.01
1790	1914861	Enterobacter sp. Sa187	Bacteria	0.01
1791	88132	Eremococcus coleocola	Bacteria	0.01
1792	1522060	Pantoea sp. 3.5.1	Bacteria	0.01
1793	1825924	Barley virus G	Viruses	0.01
1794	2371	Xylella fastidiosa	Bacteria	0.01
1795	1195085	Cronobacter phage CR5	Viruses	0.01
1796	762210	Bifidobacterium saguini	Bacteria	0.01
1797	665550	Dietzia alimentaria	Bacteria	0.01
1798	12348	Lactobacillus phage LL-H	Viruses	0.01
1799	694003	Betacoronavirus 1	Viruses	0.01
1800	317010	Enterococcus canintestini	Bacteria	0.01
1801	59749	Maize rayado fino virus	Viruses	0.01
1802	53345	Enterococcus durans	Bacteria	0.01
1803	1206110	Lactobacillus phage phiAQ113	Viruses	0.01
1804	1161906	Weissella phage phiYS61	Viruses	0.01
1805	1229204	alpha proteobacterium L41A	Bacteria	0.01
1806	114090	Pediococcus inopinatus	Bacteria	0.01
1807	1675603	Citrobacter phage Michonne	Viruses	0.01
1808	1233383	Human cosavirus	Viruses	0.01
1809	255238	Fragaria chiloensis latent virus	Viruses	0.01
1810	1849383	Psychrobacter sp. SHUES1	Bacteria	0.01
1811	264483	Phaffia rhodozyma	Eukaryota	0.01
1812	158836	Enterobacter hormaechei	Bacteria	0.01
1813	100468	Lactobacillus perolens	Bacteria	0.01
1814	712535	Selenomonas sp. oral taxon 149	Bacteria	0.01
1815	11203	Human parainfluenza virus 4	Viruses	0.01
1816	35289	Grapevine virus B	Viruses	0.01
1817	1873990	Escherichia phage vB_EcoM_Alf5	Viruses	0.01
1818	93466	Fervidobacterium pennivorans	Bacteria	0.01
1819	563037	Streptococcus sp. M143	Bacteria	0.01
1820	1499685	Bacillus andreraoultii	Bacteria	0.01

1821	393921	Porphyromonas crevioricanis	Bacteria	0.01
1822	81857	Lactobacillus selangorensis	Bacteria	0.01
1823	176291	Lactobacillus vaccinostercus	Bacteria	0.01
1824	1129192	Bacillus phage BCP8-2	Viruses	0.01
1825	481719	Lactobacillus sunkii	Bacteria	0.01
1826	1768743	Blastomonas sp. CCH8-A3	Bacteria	0.01
1827	5207	Cryptococcus neoformans	Eukaryota	0.01
1828	1647391	Streptococcus phage APCM01	Viruses	0.01
1829	1856642	Maize yellow mosaic virus	Viruses	0.01
1830	1141136	Cronobacter phage vB_CsaM_GAP32	Viruses	0.01
1831	1552735	Lactobacillus phage Ldl1	Viruses	0.01
1832	71451	Enterococcus malodoratus	Bacteria	0.01
1833	1481465	Tomato necrotic dwarf virus	Viruses	0.01
1834	1954380	Sodalis virus SO1	Viruses	0.01
1835	1911010	Escherichia virus K1H	Viruses	0.01
1836	1965306	Lagenaria siceraria endornavirus-Hubei	Viruses	0.01
1837	193122	Peanut stunt virus satellite RNA	Viruses	0.01
1838	1736702	Enterobacter sp. K66-74	Bacteria	0.01
1839	1384081	Veillonella sp. DNF00869	Bacteria	0.01
1840	38305	Corynebacterium vitaeruminis	Bacteria	0.01
1841	712414	Oribacterium sp. oral taxon 108	Bacteria	0.01
1842	73422	Streptococcus phage TP-J34	Viruses	0.01
1843	338473	Actinomyces virus Av1	Viruses	0.01
1844	1588750	Clostridiales bacterium KA00134	Bacteria	0.01
1845	12294	Pea early-browning virus	Viruses	0.01
1846	1379702	Methanobacterium sp. MB1	Archaea	0.01
1847	1414721	Clostridium jeddahense	Bacteria	0.01
1848	187978	Peru tomato mosaic virus	Viruses	0.01
1849	99480	Tetrasphaera australiensis	Bacteria	0.01
1850	11988	Turnip crinkle virus	Viruses	0.01
1851	596085	Prevotella aurantiaca	Bacteria	0.01
1852	48296	Acinetobacter pittii	Bacteria	0.01
1853	746033	Piscicoccus intestinalis	Bacteria	0.01
1854	1118963	Arthrobacter sp. Rue61a	Bacteria	0.01
1855	1681197	Arthrobacter sp. RIT-PI-e	Bacteria	0.01
1856	1293441	Lysinibacillus contaminans	Bacteria	0.01
1857	1196034	Klebsiella sp. 10982	Bacteria	0.01
1858	1739536	Corynebacterium sp. HMSC073D01	Bacteria	0.01
1859	54289	Vicia cryptic virus	Viruses	0.01
1860	1439319	Citrobacter sp. MGH 55	Bacteria	0.01
1861	326202	Vanilla distortion mosaic virus	Viruses	0.01
1862	83655	Leclercia adecarboxylata	Bacteria	0.01
1863	1336	Streptococcus equi	Bacteria	0.01
1864	1520	Clostridium beijerinckii	Bacteria	0.01
1865	1673719	Anaerococcus sp. SB3	Bacteria	0.01
1866	1051676	Erwinia phage vB_EamM-Y2	Viruses	0.01
1867	1416026	Yellow tailflower mild mottle virus	Viruses	0.01
1868	244366	Klebsiella variicola	Bacteria	0.01

1869	576789	Enterobacteria phage JSE	Viruses	0.01
1870	5082	Penicillium roqueforti	Eukaryota	0.01
1871	277944	Human coronavirus NL63	Viruses	0.01
1872	142843	Hop mosaic virus	Viruses	0.01
1873	1477000	Peptoniphilus sp. DNF00840	Bacteria	0.01
1874	489828	Enterobacteria phage WA13 sensu lato	Viruses	0.01
1875	1235640	Enterobacteria phage M	Viruses	0.01
1876	5755	Acanthamoeba castellanii	Eukaryota	0.01
1877	134533	Acinetobacter parvus	Bacteria	0.01
1878	12178	Cymbidium mosaic virus	Viruses	0.01
1879	633135	Streptococcus phage Abc2	Viruses	0.01
1880	1182762	Enterococcus sp. C1	Bacteria	0.01
1881	1813769	Salmonella phage 64795_sal3	Viruses	0.01
1882	871203	Caballeronia zhejiangensis	Bacteria	0.01
1883	37636	Thermus scotoductus	Bacteria	0.01
1884	712538	Selenomonas sp. oral taxon 478	Bacteria	0.01
1885	1203573	Propionibacterium sp. KPL1844	Bacteria	0.01
1886	62059	Shallot yellow stripe virus	Viruses	0.01
1887	72000	Kocuria rhizophila	Bacteria	0.01
1888	1341	Streptococcus ratti	Bacteria	0.01
1889	1768770	Caulobacter sp. CCH5-E12	Bacteria	0.01
1890	759620	Weissella ceti	Bacteria	0.01
1891	81464	Anaeromusa acidaminophila	Bacteria	0.01
1892	1218493	Lactobacillus kullabergensis	Bacteria	0.01
1893	1840644	Psophocarpus tetragonolobus endornavirus	Viruses	0.01
1894	566	Escherichia vulneris	Bacteria	0.01
1895	370833	Tomato torrado virus	Viruses	0.01
1896	51663	Pediococcus damnosus	Bacteria	0.01
1897	51664	Lactobacillus dextrinicus	Bacteria	0.01
1898	1923308	Hubei toti-like virus 2	Viruses	0.01
1899	302449	Corynebacterium tuscaniense	Bacteria	0.01
1900	936594	Lachnoanaerobaculum sp. ICM7	Bacteria	0.01
1901	1883202	Escherichia phage Gluttony	Viruses	0.01
1902	1739351	Corynebacterium sp. HMSC074C03	Bacteria	0.01
1903	33010	Cutibacterium avidum	Bacteria	0.01
1904	33011	Cutibacterium granulosum	Bacteria	0.01
1905	37662	Brettanomyces anomalus	Eukaryota	0.01
1906	528209	Lactobacillus kimchicus	Bacteria	0.01
1907	1817674	Geobacillus sp. 8	Bacteria	0.01
1908	1200547	Prevotella sp. RM4	Bacteria	0.01
1909	1381464	Blackberry vein banding associated virus	Viruses	0.01
1910	577	Raoultella terrigena	Bacteria	0.01
1911	187764	Escherichia virus K1-5	Viruses	0.01
1912	645687	Astrovirus VA1	Viruses	0.01
1913	1581113	Corynebacterium sp. HMSC05C01	Bacteria	0.01
1914	267633	Lactobacillus hammesii	Bacteria	0.01
1915	92444	Acute bee paralysis virus	Viruses	0.01
1916	979982	Leuconostoc sp. C2	Bacteria	0.01

1917	1513	Clostridium tetani	Bacteria	0.01
1918	1715164	Streptococcus sp. HMSC074F05	Bacteria	0.01
1919	222805	Mycobacterium chimaera	Bacteria	0.01
1920	1462608	Pseudomonas phage KPP25	Viruses	0.01
1921	1169321	Escherichia sp. KTE114	Bacteria	0.01
1922	430606	Polygonum ringspot tospovirus	Viruses	0.01
1923	77775	Salmonella phage FelixO1	Viruses	0.01
1924	1293	Staphylococcus gallinarum	Bacteria	0.01
1925	1768759	Bradyrhizobium sp. CCH4-A6	Bacteria	0.01
1926	1922578	Beihai picorna-like virus 35	Viruses	0.01
1927	84136	Gemella bergeri	Bacteria	0.01
1928	1406816	Zucchini tigre mosaic virus	Viruses	0.01
1929	49118	Candidatus Arthromitus sp. SFB-mouse	Bacteria	0.01
1930	1739611	Lactobacillus phage iLp1308	Viruses	0.01
1931	473784	Opium poppy mosaic virus	Viruses	0.01
1932	255066	Pepper veinal mottle virus	Viruses	0.01
1933	412383	Solibacillus isronensis	Bacteria	0.01
1934	1639	Listeria monocytogenes	Bacteria	0.01
1935	255248	Leuconostoc garlicum	Bacteria	0.01
1936	29484	Yersinia frederiksenii	Bacteria	0.01
1937	564	Escherichia fergusonii	Bacteria	0.01
1938	168471	Laribacter hongkongensis	Bacteria	0.01
1939	1749	Acidipropionibacterium jensenii	Bacteria	0.01
1940	157268	Helicobacter winghamensis	Bacteria	0.01
1941	331679	Pediococcus stilesii	Bacteria	0.01
1942	28375	Soil-borne wheat mosaic virus	Viruses	0.01
1943	468911	Lactobacillus hordei	Bacteria	0.01
1944	151043	Tulare apple mosaic virus	Viruses	0.01
1945	390842	Lactobacillus parafarraginis	Bacteria	0.01
1946	1768792	Erythrobacter sp. CCH5-A1	Bacteria	0.01

Table S4: All genera identified in 10,000 human stool samples

	Taxonomy ID	Genus name	SuperKingdom	Prevalence in 10,000 samples, %
1	1485	Clostridium	Bacteria	99.72
2	816	Bacteroides	Bacteria	99.57
3	572511	Blautia	Bacteria	97.62
4	469	Acinetobacter	Bacteria	97.45
5	1730	Eubacterium	Bacteria	97.16
6	375288	Parabacteroides	Bacteria	96.89
7	1357	Lactococcus	Bacteria	96.88
8	216851	Faecalibacterium	Bacteria	96.39
9	841	Roseburia	Bacteria	95.72
10	239759	Alistipes	Bacteria	95.05
11	1263	Ruminococcus	Bacteria	94.67
12	244127	Anaerotruncus	Bacteria	93.04
13	459786	Oscillibacter	Bacteria	90.88
14	1301	Streptococcus	Bacteria	90.47
15	189330	Dorea	Bacteria	88.56
16	1407607	Fusicatenibacter	Bacteria	87.22
17	561	Escherichia	Bacteria	85.92
18	1508657	Ruminiclostridium	Bacteria	84.26
19	1505663	Erysipelatoclostridium	Bacteria	83.51
20	33042	Coprococcus	Bacteria	80.66
21	292632	Subdoligranulum	Bacteria	80.59
22	29465	Veillonella	Bacteria	78.62
23	283168	Odoribacter	Bacteria	78.45
24	61170	Holdemania	Bacteria	75.80
25	1392389	Intestinimonas	Bacteria	75.67
26	102106	Collinsella	Bacteria	75.50
27	35832	Bilophila	Bacteria	74.75
28	12234	Tobamovirus	Viruses	71.58
29	447020	Adlercreutzia	Bacteria	70.39
30	1654	Actinomyces	Bacteria	69.37
31	577310	Parasutterella	Bacteria	66.65
32	1506553	Lachnoclostridium	Bacteria	65.97
33	644652	Gordonibacter	Bacteria	65.09
34	1501226	Romboutsia	Bacteria	63.54
35	84111	Eggerthella	Bacteria	61.12
36	239934	Akkermansia	Bacteria	60.64
37	207244	Anaerostipes	Bacteria	59.70
38	574697	Butyricimonas	Bacteria	59.21
39	195950	Tannerella	Bacteria	57.23
40	1350	Enterococcus	Bacteria	55.78
41	1678	Bifidobacterium	Bacteria	55.28
42	1378	Gemella	Bacteria	55.05
43	100883	Coprobacillus	Bacteria	53.50
44	613	Serratia	Bacteria	53.13
45	1926663	Phocea	Bacteria	52.64

46	397864	Barnesiella	Bacteria	52.30
47	1432051	Eisenbergiella	Bacteria	50.55
48	28050	Lachnospira	Bacteria	49.17
49	191303	Turicibacter	Bacteria	47.21
50	838	Prevotella	Bacteria	46.45
51	946234	Flavonifractor	Bacteria	42.39
52	1506577	Tyzzerella	Bacteria	42.03
53	1870884	Clostridioides	Bacteria	41.79
54	1017280	Pseudoflavonifractor	Bacteria	41.18
55	40544	Sutterella	Bacteria	40.64
56	1649459	Hungatella	Bacteria	38.28
57	580596	Butyricicoccus	Bacteria	36.67
58	1578	Lactobacillus	Bacteria	36.48
59	1905344	Ruthenibacterium	Bacteria	32.05
60	872	Desulfovibrio	Bacteria	31.93
61	577309	Paraprevotella	Bacteria	30.37
62	1935176	Angelakisella	Bacteria	28.63
63	212742	Morococcus	Bacteria	28.29
64	1924105	Neglecta	Bacteria	27.48
65	39948	Dialister	Bacteria	27.40
66	86331	Mogibacterium	Bacteria	27.03
67	1935927	Massilioclostridium	Bacteria	26.41
68	1926556	Emergencia	Bacteria	26.08
69	264995	Anaerofustis	Bacteria	25.78
70	2172	Methanobrevibacter	Archaea	25.70
71	4930	Saccharomyces	Eukaryota	25.44
72	830	Butyrivibrio	Bacteria	24.25
73	33024	Phascolarctobacterium	Bacteria	23.62
74	420345	Lactonifactor	Bacteria	22.77
75	135858	Catenibacterium	Bacteria	22.04
76	1573535	Holdemanella	Bacteria	21.87
77	12163	Carlavirus	Viruses	20.89
78	1473205	Senegalimassilia	Bacteria	19.55
79	1380	Atopobium	Bacteria	19.32
80	724	Haemophilus	Bacteria	17.80
81	846	Oxalobacter	Bacteria	17.77
82	848	Fusobacterium	Bacteria	16.98
83	990721	Christensenella	Bacteria	16.65
84	2753	Synergistes	Bacteria	16.55
85	12316	Ilarvirus	Viruses	16.47
86	1470349	Candidatus Stoquefichus	Bacteria	16.10
87	12176	Potexvirus	Viruses	15.91
88	1243	Leuconostoc	Bacteria	15.85
89	270497	Catabacter	Bacteria	15.67
90	590	Salmonella	Bacteria	15.61
91	34072	Variovorax	Bacteria	15.51
92	1348911	Coprobacter	Bacteria	15.41
93	310748	Endornavirus	Viruses	15.19

94	1940255	Fournierella	Bacteria	15.03
95	836	Porphyromonas	Bacteria	13.98
96	1924081	Bariatricus	Bacteria	12.65
97	904	Acidaminococcus	Bacteria	11.77
98	1472649	Dielma	Bacteria	11.73
99	194	Campylobacter	Bacteria	11.45
100	12967	Blastocystis	Eukaryota	11.37
101	76833	Actinobaculum	Bacteria	10.88
102	1505657	Intestinibacter	Bacteria	9.92
103	84108	Slackia	Bacteria	9.78
104	162289	Peptoniphilus	Bacteria	9.54
105	119164	Polerovirus	Viruses	9.33
106	1926672	Prevotellamassilia	Bacteria	8.81
107	248744	Marvinbryantia	Bacteria	8.55
108	906	Megasphaera	Bacteria	8.50
109	150022	Finegoldia	Bacteria	8.31
110	12195	Potyvirus	Viruses	8.15
111	1511809	Betapartitivirus	Viruses	8.14
112	1253	Pediococcus	Bacteria	7.61
113	1929045	Traorella	Bacteria	7.29
114	133925	Olsenella	Bacteria	7.07
115	1635148	Sanguibacteroides	Bacteria	6.59
116	1715798	Levyella	Bacteria	6.59
117	158846	Megamonas	Bacteria	6.52
118	165779	Anaerococcus	Bacteria	6.51
119	580024	Enterorhabdus	Bacteria	6.47
120	123375	Solobacterium	Bacteria	6.41
121	186767	Narnavirus	Viruses	6.19
122	1257	Peptostreptococcus	Bacteria	6.10
123	508459	Cloacibacillus	Bacteria	5.55
124	1573534	Faecalitalea	Bacteria	5.54
125	1080709	Methanomassiliicoccus	Archaea	5.25
126	46255	Weissella	Bacteria	5.13
127	1763	Mycobacterium	Bacteria	5.09
128	67753	Crinivirus	Viruses	4.87
129	35829	Acetivibrio	Bacteria	4.55
130	2316	Methanosphaera	Archaea	4.34
131	43996	Catonella	Bacteria	4.32
132	40276	Trichovirus	Viruses	4.32
133	12137	Sobemovirus	Viruses	4.29
134	286	Pseudomonas	Bacteria	4.28
135	1686313	Fenollaria	Bacteria	4.26
136	281915	Curvibacter	Bacteria	4.06
137	1929083	Tidjanibacter	Bacteria	4.05
138	1926659	Mediterranea	Bacteria	4.03
139	12304	Cucumovirus	Viruses	3.93
140	1935200	Merdibacter	Bacteria	3.87
141	1743	Propionibacterium	Bacteria	3.77

142	129725	Foveavirus	Viruses	3.51
143	1164882	Lachnoanaerobaculum	Bacteria	3.45
144	543311	Parvimonas	Bacteria	3.41
145	1923867	Gabonia	Bacteria	3.26
146	140295	Varicosavirus	Viruses	2.96
147	28138	Rikenella	Bacteria	2.82
148	46123	Abiotrophia	Bacteria	2.76
149	12174	Capillovirus	Viruses	2.75
150	1965226	Urmitella	Bacteria	2.73
151	1716	Corynebacterium	Bacteria	2.66
152	52225	Mitsuokella	Bacteria	2.53
153	1472762	Enorma	Bacteria	2.41
154	570	Klebsiella	Bacteria	2.38
155	12141	Tombusvirus	Viruses	2.37
156	104394	Picobirnavirus	Viruses	2.37
157	2701	Gardnerella	Bacteria	2.35
158	419014	Alloscardovia	Bacteria	2.35
159	1911601	Gammacarmovirus	Viruses	2.31
160	1386	Bacillus	Bacteria	2.16
161	217160	Ampelovirus	Viruses	2.08
162	620	Shigella	Bacteria	2.08
163	12270	Nepovirus	Viruses	2.08
164	13687	Sphingomonas	Bacteria	2.05
165	5721	Trichomonas	Eukaryota	2.00
166	265975	Oribacterium	Bacteria	1.97
167	1922299	Beduini	Bacteria	1.96
168	642	Aeromonas	Bacteria	1.89
169	1511808	Alphapartitivirus	Viruses	1.86
170	1573536	Faecalicoccus	Bacteria	1.82
171	1903506	Drancourtella	Bacteria	1.79
172	1929297	Bittarella	Bacteria	1.68
173	1935188	Libanicoccus	Bacteria	1.66
174	186457	Aureusvirus	Viruses	1.63
175	48736	Ralstonia	Bacteria	1.58
176	1283313	Alloprevotella	Bacteria	1.46
177	1926651	Culturomica	Bacteria	1.40
178	11611	Tospovirus	Viruses	1.39
179	12320	Alfamovirus	Viruses	1.37
180	32207	Rothia	Bacteria	1.32
181	12300	Bromovirus	Viruses	1.29
182	156207	Tritimovirus	Viruses	1.28
183	1470353	Candidatus Soleaferrea	Bacteria	1.26
184	184869	Varibaculum	Bacteria	1.22
185	209	Helicobacter	Bacteria	1.16
186	12160	Closterovirus	Viruses	1.14
187	196081	Scardovia	Bacteria	1.11
188	1273095	Anaerosphaera	Bacteria	1.09
189	140568	Allexivirus	Viruses	1.04

190	1637257	Mageeibacillus	Bacteria	0.94
191	963	Herbaspirillum	Bacteria	0.92
192	1912217	Pseudopropionibacterium	Bacteria	0.89
193	1505652	Terrisporobacter	Bacteria	0.87
194	1847725	Lawsonella	Bacteria	0.86
195	142786	Norovirus	Viruses	0.82
196	416916	Aggregatibacter	Bacteria	0.81
197	5758	Entamoeba	Eukaryota	0.79
198	2747	Carnobacterium	Bacteria	0.79
199	662	Vibrio	Bacteria	0.75
200	168808	Sneathia	Bacteria	0.75
201	13366	Brettanomyces	Eukaryota	0.74
202	156454	Anaeroglobus	Bacteria	0.69
203	2129	Ureaplasma	Bacteria	0.69
204	34104	Streptobacillus	Bacteria	0.69
205	75	Caulobacter	Bacteria	0.68
206	482	Neisseria	Bacteria	0.67
207	39760	Idaeovirus	Viruses	0.65
208	1924093	Anaeromassilibacillus	Bacteria	0.65
209	156973	Dysgonomonas	Bacteria	0.61
210	12036	Luteovirus	Viruses	0.61
211	60919	Sanguibacter	Bacteria	0.59
212	43987	Geotrichum	Eukaryota	0.59
213	194960	Kobuvirus	Viruses	0.58
214	1937664	Criibacterium	Bacteria	0.56
215	12258	Comovirus	Viruses	0.56
216	588605	Robinsoniella	Bacteria	0.55
217	1299311	Betanecrovirus	Viruses	0.55
218	117563	Granulicatella	Bacteria	0.54
219	1911312	Gabonibacter	Bacteria	0.54
220	544	Citrobacter	Bacteria	0.53
221	10639	Caulimovirus	Viruses	0.52
222	1279	Staphylococcus	Bacteria	0.51
223	249185	Maculavirus	Viruses	0.51
224	698776	Cellulosilyticum	Bacteria	0.50
225	1161127	Murdochiella	Bacteria	0.48
226	1623304	Sfi21dt1virus	Viruses	0.48
227	1582879	Ezakiella	Bacteria	0.46
228	1279388	Kandleria	Bacteria	0.46
229	40323	Stenotrophomonas	Bacteria	0.45
230	29521	Brachyspira	Bacteria	0.45
231	1291539	Candidatus Methanomethylophilus	Archaea	0.45
232	638847	Pyramidobacter	Bacteria	0.43
233	1230390	Poacevirus	Viruses	0.42
234	35823	Arthrospira	Bacteria	0.42
235	12059	Enterovirus	Viruses	0.41
236	12269	Fabavirus	Viruses	0.41
237	32067	Leptotrichia	Bacteria	0.41

238	970	Selenomonas	Bacteria	0.40
239	547	Enterobacter	Bacteria	0.40
240	519427	Sharpea	Bacteria	0.38
241	11007	Totivirus	Viruses	0.37
242	2093	Mycoplasma	Bacteria	0.35
243	144051	Cripavirus	Viruses	0.35
244	95341	Sapovirus	Viruses	0.35
245	137757	Ipomovirus	Viruses	0.35
246	84162	Cryptobacterium	Bacteria	0.35
247	4919	Pichia	Eukaryota	0.34
248	1535326	Candida	Eukaryota	0.34
249	12051	Marafivirus	Viruses	0.32
250	47670	Lautropia	Bacteria	0.32
251	1213720	Stomatobaculum	Bacteria	0.30
252	39742	Barnavirus	Viruses	0.29
253	1935192	Mobilibacterium	Bacteria	0.29
254	674963	Succinatimonas	Bacteria	0.29
255	528	Ochrobactrum	Bacteria	0.28
256	129337	Geobacillus	Bacteria	0.28
257	186532	C2virus	Viruses	0.28
258	196082	Parascardovia	Bacteria	0.28
259	1945592	Lagierella	Bacteria	0.28
260	12148	Tymovirus	Viruses	0.27
261	29832	Hanseniaspora	Eukaryota	0.26
262	2050	Mobiluncus	Bacteria	0.25
263	66831	Facklamia	Bacteria	0.25
264	12293	Tobravirus	Viruses	0.24
265	83770	Succinivibrio	Bacteria	0.23
266	57493	Kocuria	Bacteria	0.23
267	1016	Capnocytophaga	Bacteria	0.22
268	39734	Umbravirus	Viruses	0.22
269	583	Proteus	Bacteria	0.20
270	1769710	Sellimonas	Bacteria	0.20
271	28263	Arcanobacterium	Bacteria	0.20
272	1472763	Kallipyga	Bacteria	0.19
273	5073	Penicillium	Eukaryota	0.19
274	581	Morganella	Bacteria	0.19
275	1849828	Paeniclostridium	Bacteria	0.19
276	1973274	Niameybacter	Bacteria	0.19
277	33882	Microbacterium	Bacteria	0.18
278	174708	Allobaculum	Bacteria	0.18
279	1111	Porphyrobacter	Bacteria	0.18
280	1375	Aerococcus	Bacteria	0.17
281	413970	Anulavirus	Viruses	0.17
282	82373	Anaerovibrio	Bacteria	0.16
283	254250	Isoptericola	Bacteria	0.16
284	5740	Giardia	Eukaryota	0.16
285	428711	Jonquetella	Bacteria	0.16

286	300275	Lachancea	Eukaryota	0.16
287	44259	Filifactor	Bacteria	0.15
288	232799	Iflavirus	Viruses	0.15
289	180162	Cetobacterium	Bacteria	0.15
290	12289	Enamovirus	Viruses	0.15
291	1069494	Trueperella	Bacteria	0.15
292	71245	Kazachstania	Eukaryota	0.15
293	5340	Agaricus	Eukaryota	0.14
294	241189	Hespellia	Bacteria	0.13
295	579	Kluyvera	Bacteria	0.13
296	1696	Brevibacterium	Bacteria	0.12
297	184751	Vitivirus	Viruses	0.12
298	4958	Debaryomyces	Eukaryota	0.12
299	186886	Pomovirus	Viruses	0.12
300	150247	Anoxybacillus	Bacteria	0.11
301	1623303	Sfi11virus	Viruses	0.11
302	46254	Oenococcus	Bacteria	0.11
303	1653174	Actinotignum	Bacteria	0.11
304	113286	Pseudoramibacter	Bacteria	0.10
305	559173	Fructobacillus	Bacteria	0.10
306	374	Bradyrhizobium	Bacteria	0.09
307	604195	Cyberlindnera	Eukaryota	0.09
308	49082	Candidatus Arthromitus	Bacteria	0.09
309	46205	Pseudobutyrivibrio	Bacteria	0.09
310	39739	Machlomovirus	Viruses	0.08
311	177971	Shuttleworthia	Bacteria	0.08
312	106589	Cupriavidus	Bacteria	0.07
313	310684	Cheravirus	Viruses	0.07
314	586418	Cosavirus	Viruses	0.07
315	4910	Kluyveromyces	Eukaryota	0.07
316	29330	Alicyclobacillus	Bacteria	0.07
317	586	Providencia	Bacteria	0.07
318	43994	Johnsonella	Bacteria	0.07
319	12266	Dianthovirus	Viruses	0.07
320	54066	Xylophilus	Bacteria	0.07
321	1198140	Felixo1virus	Viruses	0.07
322	118747	Bulleidia	Bacteria	0.07
323	57499	Kytococcus	Bacteria	0.07
324	675073	Torradovirus	Viruses	0.07
325	253238	Ethanoligenens	Bacteria	0.07
326	283	Comamonas	Bacteria	0.07
327	45153	Westerdykella	Eukaryota	0.07
328	1663	Arthrobacter	Bacteria	0.06
329	186768	Mitovirus	Viruses	0.06
330	187214	Soymovirus	Viruses	0.06
331	5748	Nannochloropsis	Eukaryota	0.06
332	1911599	Alphacarmovirus	Viruses	0.06
333	12916	Acidovorax	Bacteria	0.06

334	413496	Cronobacter	Bacteria	0.06
335	1511810	Deltapartitivirus	Viruses	0.06
336	203470	Tepidiphilus	Bacteria	0.06
337	1922209	Triatovirus	Viruses	0.06
338	1299309	Alphanecrovirus	Viruses	0.06
339	1562	Desulfotomaculum	Bacteria	0.06
340	28196	Arcobacter	Bacteria	0.06
341	5506	Fusarium	Eukaryota	0.05
342	688449	Salivirus	Viruses	0.05
343	1542744	Gemycircularvirus	Viruses	0.05
344	810	Chlamydia	Bacteria	0.05
345	289201	Pontibacillus	Bacteria	0.05
346	12008	Allolevivirus	Viruses	0.05
347	1912215	Acidipropionibacterium	Bacteria	0.05
348	542837	Sp6virus	Viruses	0.05
349	11990	Levivirus	Viruses	0.05
350	39725	Circovirus	Viruses	0.05
351	568	Hafnia	Bacteria	0.05
352	28105	Sinorhizobium	Bacteria	0.05
353	138954	Parechovirus	Viruses	0.05
354	39749	Hypovirus	Viruses	0.05
355	1910992	K1gvirus	Viruses	0.05
356	11305	Cytorhabdovirus	Viruses	0.05
357	675845	Emaravirus	Viruses	0.05
358	1505660	Asaccharospora	Bacteria	0.05
359	333750	Alphapapillomavirus	Viruses	0.05
360	1910954	Phix174microvirus	Viruses	0.05
361	88129	Ophiovirus	Viruses	0.05
362	114248	Tepidimonas	Bacteria	0.05
363	59732	Chryseobacterium	Bacteria	0.05
364	5475	Candida	Eukaryota	0.05
365	10814	Begomovirus	Viruses	0.05
366	1921117	Nonagvirus	Viruses	0.05
367	374468	Nakaseomyces	Eukaryota	0.05
368	140625	Lachnobacterium	Bacteria	0.05
369	157	Treponema	Bacteria	0.05
370	79603	Denitrobacterium	Bacteria	0.04
371	338	Xanthomonas	Bacteria	0.04
372	1794910	Anaerosporomusa	Bacteria	0.04
373	160674	Raoultella	Bacteria	0.04
374	3011	Fucus	Eukaryota	0.04
375	146	Spirochaeta	Bacteria	0.04
376	1940395	Caenibacillus	Bacteria	0.04
377	699166	Citrivirus	Viruses	0.04
378	653683	Anaerosporobacter	Bacteria	0.04
379	143901	Benyvirus	Viruses	0.04
380	28895	Thermoanaerobacterium	Bacteria	0.04
381	1269	Micrococcus	Bacteria	0.04

382	1283209	Alphacarmotetravirus	Viruses	0.04
383	2147	Acholeplasma	Bacteria	0.04
384	1920860	Kp36virus	Viruses	0.04
385	2192	Methanocorpusculum	Archaea	0.04
386	1033	Afipia	Bacteria	0.04
387	186188	Xylanimonas	Bacteria	0.04
388	501783	Cloacibacterium	Bacteria	0.04
389	10509	Mastadenovirus	Viruses	0.04
390	10813	Curtovirus	Viruses	0.04
391	674962	Bacillarnavirus	Viruses	0.04
392	1921122	Nonanavirus	Viruses	0.04
393	538	Eikenella	Bacteria	0.04
394	31983	Helcococcus	Bacteria	0.04
395	36910	Clavispora	Eukaryota	0.04
396	256806	Curvibasidium	Eukaryota	0.04
397	1729679	Faecalibaculum	Bacteria	0.03
398	270	Thermus	Bacteria	0.03
399	13334	Anaerobiospirillum	Bacteria	0.03
400	2160	Methanobacterium	Archaea	0.03
401	12050	Waikavirus	Viruses	0.03
402	227307	Gyrovirus	Viruses	0.03
403	5352	Lentinula	Eukaryota	0.03
404	53335	Pantoea	Bacteria	0.03
405	1914852	V5virus	Viruses	0.03
406	69965	Macrococcus	Bacteria	0.03
407	32008	Burkholderia	Bacteria	0.03
408	2717	Cardiobacterium	Bacteria	0.03
409	373053	Calditerricola	Bacteria	0.03
410	28453	Sphingobacterium	Bacteria	0.03
411	378210	Methyloversatilis	Bacteria	0.03
412	909928	Negativicoccus	Bacteria	0.03
413	248038	Mucispirillum	Bacteria	0.03
414	4895	Schizosaccharomyces	Eukaryota	0.03
415	1792239	Caviibacter	Bacteria	0.03
416	1914295	Prunevirus	Viruses	0.03
417	249588	Mamastrovirus	Viruses	0.03
418	165812	Sporanaerobacter	Bacteria	0.03
419	5410	Cystofilobasidium	Eukaryota	0.02
420	187218	T5virus	Viruses	0.02
421	745	Pasteurella	Bacteria	0.02
422	10663	T4virus	Viruses	0.02
423	477967	Phikmvvirus	Viruses	0.02
424	104766	Nanovirus	Viruses	0.02
425	2289	Desulfobacter	Bacteria	0.02
426	1913651	Rb49virus	Viruses	0.02
427	4948	Torulaspora	Eukaryota	0.02
428	16	Methylophilus	Bacteria	0.02
429	1573	Clavibacter	Bacteria	0.02

430	1159323	Macellibacteroides	Bacteria	0.02
431	1912216	Cutibacterium	Bacteria	0.02
432	61434	Dehalococcoides	Bacteria	0.02
433	201096	Alicycliphilus	Bacteria	0.02
434	10912	Rotavirus	Viruses	0.02
435	1513308	Velarivirus	Viruses	0.02
436	1920753	G7cvirus	Viruses	0.02
437	1913599	Peptoanaerobacter	Bacteria	0.02
438	13075	Globicatella	Bacteria	0.02
439	80865	Delftia	Bacteria	0.02
440	1827195	Caballeronia	Bacteria	0.02
441	41273	Tissierella	Bacteria	0.02
442	1922243	Sextaecvirus	Viruses	0.01
443	203133	Propionimicrobium	Bacteria	0.01
444	1611681	Mucinivorans	Bacteria	0.01
445	186536	Ebolavirus	Viruses	0.01
446	278028	Naumovozyma	Eukaryota	0.01
447	1707	Cellulomonas	Bacteria	0.01
448	1213379	Aparavirus	Viruses	0.01
449	107449	Phaffia	Eukaryota	0.01
450	83654	Leclercia	Bacteria	0.01
451	144193	Turicella	Bacteria	0.01
452	44249	Paenibacillus	Bacteria	0.01
453	1960084	Rodentibacter	Bacteria	0.01
454	400634	Lysinibacillus	Bacteria	0.01
455	1911600	Betacarmovirus	Viruses	0.01
456	227979	Jeotgalicoccus	Bacteria	0.01
457	53457	Janibacter	Bacteria	0.01
458	39744	Rubulavirus	Viruses	0.01
459	187217	T1virus	Viruses	0.01
460	110456	T7virus	Viruses	0.01
461	766728	Meyerozyma	Eukaryota	0.01
462	551	Erwinia	Bacteria	0.01
463	1914851	Se1virus	Viruses	0.01
464	171412	Eremococcus	Bacteria	0.01
465	497	Psychrobacter	Bacteria	0.01
466	675062	Mycoflexivirus	Viruses	0.01
467	1055323	Aeribacillus	Bacteria	0.01
468	1835	Saccharopolyspora	Bacteria	0.01
469	261933	Pleomorphomonas	Bacteria	0.01
470	85651	Omegatetravirus	Viruses	0.01
471	702	Plesiomonas	Bacteria	0.01
472	99479	Tetrasphaera	Bacteria	0.01
473	1920774	Kp34virus	Viruses	0.01
474	155493	Gallibacterium	Bacteria	0.01
475	186844	Panicovirus	Viruses	0.01
476	1637	Listeria	Bacteria	0.01
477	629	Yersinia	Bacteria	0.01

478	5543	Trichoderma	Eukaryota	0.01
479	36739	Dermabacter	Bacteria	0.01
480	657	Photobacterium	Bacteria	0.01
481	190323	Plantibacter	Bacteria	0.01
482	1910951	Alpha3microvirus	Viruses	0.01
483	168470	Laribacter	Bacteria	0.01
484	81463	Anaeromusa	Bacteria	0.01
485	1322061	Rhizoctonia	Eukaryota	0.01
486	55506	Schwartzia	Bacteria	0.01
487	74380	Undaria	Eukaryota	0.01
488	1041	Erythrobacter	Bacteria	0.01
489	204037	Dickeya	Bacteria	0.01
490	5754	Acanthamoeba	Eukaryota	0.01
491	862	Syntrophomonas	Bacteria	0.01
492	582472	Wohlfahrtiimonas	Bacteria	0.01
493	1742989	Glutamicibacter	Bacteria	0.01
494	985001	Piscicoccus	Bacteria	0.01
495	39731	Bymovirus	Viruses	0.01
496	693996	Alphacoronavirus	Viruses	0.01
497	694002	Betacoronavirus	Viruses	0.01
498	1911929	Bc431virus	Viruses	0.01
499	1647	Erysipelothrix	Bacteria	0.01
500	10861	Inovirus	Viruses	0.01
501	1623289	Hk578virus	Viruses	0.01
502	407	Methylobacterium	Bacteria	0.01
503	2207	Methanosarcina	Archaea	0.01
504	29393	Dolosigranulum	Bacteria	0.01
505	635	Edwardsiella	Bacteria	0.01
506	94008	Thermicanus	Bacteria	0.01
507	90243	Oligella	Bacteria	0.01
508	2737	Vagococcus	Bacteria	0.01
509	33057	Thauera	Bacteria	0.01
510	5206	Cryptococcus	Eukaryota	0.01
511	150333	Thermobrachium	Bacteria	0.01
512	1279384	Eggerthia	Bacteria	0.01
513	156208	Macluravirus	Viruses	0.01
514	1930845	Ndongobacter	Bacteria	0.01
515	5010	Taphrina	Eukaryota	0.01
516	1330546	Pluralibacter	Bacteria	0.01
517	79808	Coniochaeta	Eukaryota	0.01
518	44000	Caldicellulosiruptor	Bacteria	0.01
519	371730	N4virus	Viruses	0.01
520	82802	Trichococcus	Bacteria	0.01
521	5455	Colletotrichum	Eukaryota	0.01
522	169998	Phakopsora	Eukaryota	0.01
523	150203	Blastomonas	Bacteria	0.01
524	12291	Furovirus	Viruses	0.01
525	119492	Pecluvirus	Viruses	0.01

526	2422	Fervidobacterium	Bacteria	0.01
527	41275	Brevundimonas	Bacteria	0.01
528	489909	Nosocomiicoccus	Bacteria	0.01

Table S5: Top 100 KEGG functions in 10,000 human stool samples

	KEGG annotation	Prevalence in 10000 samples, %
1	K00936	100.00
2	K01190	99.99
3	K03046	99.99
4	K02355	99.99
5	K00540	99.99
6	K03695	99.99
7	K03296	99.99
8	K01362	99.98
9	K02358	99.98
10	K06950	99.98
11	K03737	99.98
12	K00975	99.98
13	K02469	99.97
14	K00754	99.97
15	K03070	99.97
16	K00705	99.97
17	K02519	99.97
18	K00599	99.97
19	K03043	99.97
20	K01834	99.96
21	K01448	99.96
22	K03654	99.96
23	K04043	99.96
24	K00100	99.96
25	K00134	99.96
26	K13993	99.96
27	K03686	99.96
28	K00262	99.96
29	K01784	99.96
30	K03088	99.96
31	K02343	99.96
32	K01006	99.96
33	K00962	99.95
34	K06400	99.95
35	K07114	99.95
36	K01952	99.95
37	K01624	99.95
38	K00688	99.95

39	K08303	99.95
40	K02004	99.95
41	K03530	99.95
42	K07720	99.95
43	K03086	99.95
44	K01417	99.95
45	K01238	99.95
46	K04077	99.95
47	K00615	99.95
48	K03696	99.95
49	K05808	99.95
50	K02945	99.95
51	K04079	99.95
52	K02035	99.95
53	K02529	99.95
54	K03798	99.95
55	K01840	99.94
56	K00951	99.94
57	K05349	99.94
58	K01785	99.94
59	K01610	99.94
60	K02337	99.94
61	K00845	99.94
62	K00700	99.94
63	K08884	99.93
64	K02014	99.93
65	K01951	99.93
66	K02470	99.93
67	K01689	99.93
68	K00532	99.93
69	K07335	99.92
70	K07636	99.92
71	K01262	99.92
72	K01955	99.92
73	K06147	99.92
74	K02863	99.92
75	K00680	99.92
76	K02967	99.92
77	K12373	99.92
78	K00656	99.92
79	K00945	99.92

80	K02886	99.92
81	K01810	99.91
82	K01270	99.91
83	K02982	99.91
84	K02111	99.91
85	K01897	99.91
86	K01925	99.91
87	K02027	99.91
88	K00927	99.91
89	K03545	99.91
90	K03733	99.91
91	K01187	99.91
92	K03076	99.90
93	K03555	99.90
94	K02600	99.90
95	K07133	99.90
96	K03531	99.90
97	K02888	99.90
98	K03701	99.90
99	K02112	99.90
100	K00850	99.90
